# Selective disruption of ROR**γ**t-CBF**β** interaction by IMU-935 prevents ROR**γ**t-dependent Th17 autoimmunity but not thymocyte development

**DOI:** 10.1172/JCI185942

**Published:** 2026-01-02

**Authors:** Hongmin Wu, Xiancai Zhong, Ning Ma, Zhiheng He, Guanpeng Wang, Geming Lu, Yate-Ching Yuan, Wencan Zhang, Yun Shi, Nagarajan Vaidehi, Evelyn Peelen, Tanja Wulff, Christian Gege, Hella Kohlhof, Daniel Vitt, Yousang Gwack, Ichiro Taniuchi, Hai-Hui Xue, Zuoming Sun

**Affiliations:** 1 Department of Immunology & Theranostics, Arthur Riggs Diabetes & Metabolism Research Institute,; 2 Department of Computational and Quantitative Medicine,; 3 Department of Molecular & Cellular Endocrinology, Arthur Riggs Diabetes & Metabolism Research Institute, and; 4 Translational Bioinformatics, Department of Computational Quantitative Medicine, Beckman Research Institute of the City of Hope, Duarte, California, USA.; 5 Immunic AG, Gräfelfing, Germany.; 6 Department of Physiology, David Geffen School of Medicine, UCLA, Los Angeles, California, USA.; 7 Laboratory for Transcriptional Regulation, RIKEN Center for Integrative Medical Science, Yokohama, Kanagawa, Japan.; 8 Center for Discovery and Innovation, Hackensack University Medical Center, Nutley, New Jersey, USA.

**Keywords:** Autoimmunity, Immunology, Autoimmune diseases, Drug therapy

## Abstract

RORγt is a key transcription factor regulating both Th17 differentiation and thymocyte development. Although Th17 cells drive autoimmune diseases, inhibiting RORγt to treat autoimmunity also disrupts thymocyte development and can cause lethal thymic lymphoma. We identified a previously unreported RORγt cofactor, CBFβ, and a highly selective RORγt inhibitor, IMU-935, that preferentially disrupt the RORγt-CBFβ interaction in Th17 cells but not thymocytes. This interaction is essential for RORγt function; mice with a RORγt mutant unable to bind CBFβ had impaired Th17 differentiation, were resistant to experimental autoimmune encephalomyelitis (EAE), and had defective thymocyte development. IMU-935 inhibited Th17 differentiation and reduced EAE severity without affecting thymocyte development by selectively targeting the RORγt-CBFβ interaction in Th17 cells but not in thymocytes. This differential effect arose because different concentrations of IMU-935 were required to disrupt the interaction in Th17 cells versus thymocytes, due to varying levels of RUNX1 that compete with RORγt for CBFβ binding. This study reveals an unreported mechanism for RORγt regulation and a selective RORγt inhibitor that prevents Th17-driven autoimmunity without the risk of lethal lymphoma from thymocyte disruption.

## Abstract 2

An inverse agonist IMU-935 (izumerogant) inhibits ROR<span>γ</span>t-dependent Th17 differentiation but not thymocyte development via selective disruption of Cbf<span>β</span>-RORγt interaction

## Introduction

Th17 cells produce multiple inflammatory cytokines such as IL-17A/F, IL-22, GM-CSF, and IFN-γ. Consequently, Th17 cells contribute to the pathogenesis of autoimmune diseases such as collagen-induced arthritis (1) and experimental autoimmune encephalomyelitis (EAE) in mice (2). Evidence also supports the pathogenic role of Th17 cells in multiple human autoimmune disorders, including multiple sclerosis (3), inflammatory bowel disease (4), and psoriasis (5). Given their role in autoimmune diseases, targeting Th17 cells represents a promising therapeutic strategy (6). Indeed, inhibiting the Th17 pathway with neutralizing antibodies against IL-17 or IL-23 has proven effective for treating autoimmune psoriasis, multiple sclerosis, and uveitis (7–11). However, mAb therapy, despite its high specificity, has limitations. Immunogenicity of mAbs can affect their efficiency, safety, and disposition. Additionally, mAbs are expensive to produce and typically require intravenous administration. Conversely, small molecules can better access difficult-to-reach tissues, including in the CNS, due to their smaller size. They are also more affordable to produce and can be administered orally. Therefore, there is a strong medical need for small-molecule Th17 inhibitors.

Retinoid-related orphan receptor γ t (RORγt) is a key transcription factor that directs Th17 differentiation (12–18). RORγt-deficient mice lack Th17 cells and, consequently, are resistant to Th17-mediated autoimmune diseases, including EAE (14, 16, 19–21). Additionally, RORγt is crucial for IL-17 production from ILC3s, γδT, and NKT innate lymphocytes, which contribute to autoimmunity (22). This makes RORγt an attractive drug target for managing Th17-mediated autoimmunity (20, 23). However, RORγt also plays a less known but equally important role in regulating thymic T cell development (24–30). We first reported the role of RORγt in thymocyte development using *RORγ^–/–^* mice (24, 27). Thymocyte development progresses through multiple defined stages. CD4^+^CD8^+^ double-positive (DP) cells, which account for 90% of total thymocytes, express the T cell receptor (TCR) and undergo positive and negative selection (31). RORγt is significantly upregulated in DP thymocytes to enhance their survival, being necessary for completing their thymic development. In *RORγt^–/–^* mice, DP cells undergo premature apoptosis due to reduced expression of the antiapoptotic molecule Bcl-xL (24, 32). A high frequency of *RORγt^–/–^* mice eventually develop lethal thymic lymphoma due to abnormal thymocyte development (32), suggesting that RORγt inhibitors can induce lymphoma. Indeed, an RORγt inhibitor developed by Novartis affected thymocyte development similarly to *RORγt^–/–^* mice, including accelerated thymocyte apoptosis and thymic lymphoma (33). Therefore, it is crucial to develop RORγt inhibitors that specifically target Th17 differentiation without affecting thymocyte development.

Numerous small-molecule pharmacological RORγt inhibitors have been developed for clinical application via their ability to inhibit overall RORγt-dependent transcription activation (34). However, while targeting Th17 cells, these inhibitors also disrupt RORγt function in thymocytes, impairing T cell development and potentially leading to lethal thymic lymphoma (33, 35). Our previous studies demonstrated that RORγt uses distinct mechanisms to regulate the function of Th17 cells and thymocytes (14, 16, 18). This indicates the potential to develop RORγt inhibitors that prevent Th17-mediated autoimmunity without interfering with thymocyte development, thereby avoiding the risk of lymphoma.

In this study, we identified core-binding factor β (CBFβ) as a cofactor for RORγt and showed that the RORγt inhibitor IMU-935 preferentially disrupted the RORγt-CBFβ interaction in Th17 cells but not in thymocytes, due to presence of different levels of runt-related transcription factor 1 (RUNX1), which competes with RORγt for binding to CBFβ in these 2 types of cells. Therefore, IMU-935 is a highly selective RORγt inhibitor, inhibiting Th17 cell–mediated autoimmunity without affecting RORγt-dependent thymocyte function, and thus does not induce lethal thymic lymphoma.

## Results

### RORγt inhibitor IMU-935 potently inhibits IL-17 production from PBMCs.

**Figure 1:**
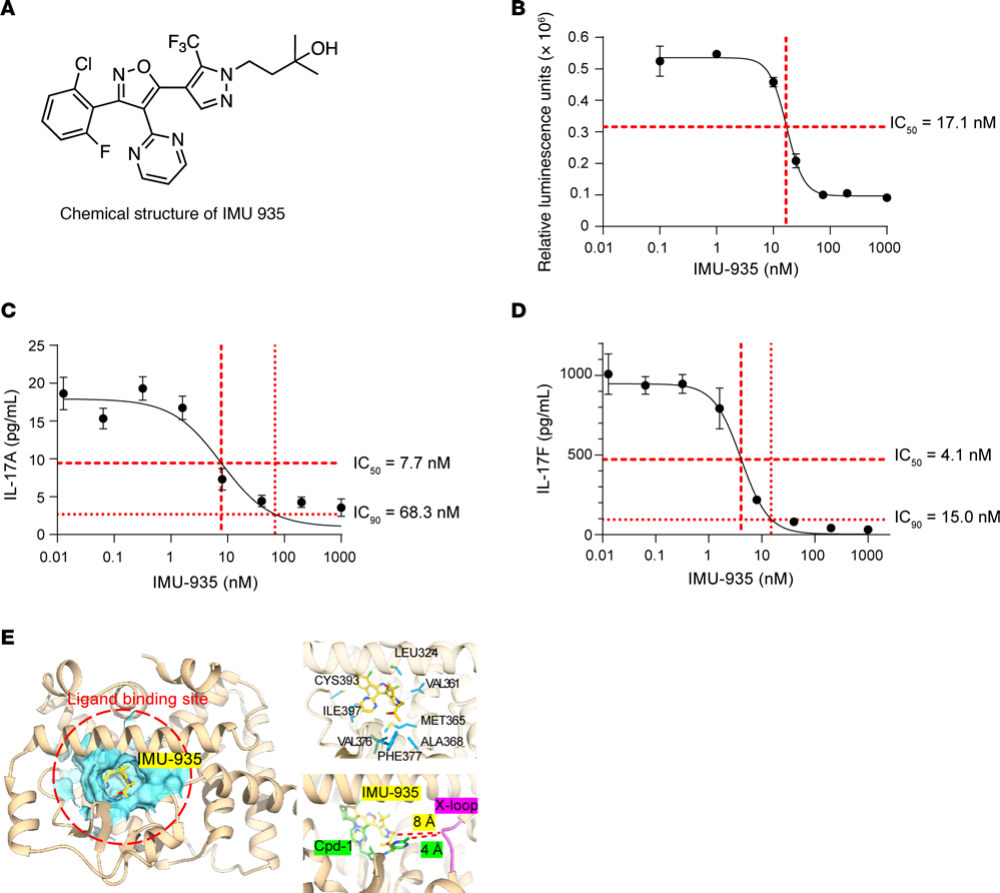
IMU-935 potently inhibits IL-17 production from human PBMCs. (**A**) Chemical structure of IMU-935. (**B**) A dose-response curve for IMU-935–inhibited RORγt luciferase reporter activity in the INDIGO assay in the presence of different concentrations of IMU-935 (*n* = 2 technical replicates per point). (**C** and **D**) A dose-response curve for IMU-935–inhibited IL-17A (**C**) or IL-17F (**D**) production from phytohaemagglutinin-stimulated human PBMCs for 2 days and measured by Luminex. Data shown are from 1 representative donor (*n* = 3 technical replicates per point). Summary data from the 4 donors are shown in Supplemental Figure 1A. (**E**) Cocrystal structure of RORγt and IMU-935. Left: A crystal structure of IMU-935 (yellow sticks) complexed with the RORγt LBD. The IMU-935 binding site is accentuated by a red circle, and the IMU-935 binding pocket’s morphological contours are delineated by a blue surface. Top right: An enhanced close-up representation reveals the spatial orientation of IMU-935 (yellow sticks) and its interactions with neighboring structures and amino acids (blue sticks). Lower right: A superimposition of IMU-935 with compound-1 (Cpd-1) is depicted in the binding pocket of RORγt. The distance between IMU-935 or Cpd-1 and the X-loop (indicated in magenta) located between H1 and H2 is illustrated by dashed lines.

IMU-935 is a lipophilic compound featuring a tricyclic central element (phenyl/isoxazole/pyrazole) with a polar hydroxy group at the periphery (Figure 1A). IMU-935 inhibited an RORγt luciferase reporter with an IC_50_ of 17.1 nM (Figure 1B) and potently inhibited IL-17A (IC_50_ = 7.7 nM) (Figure 1C), IL-17F (IC_50_ = 4.1 nM) (Figure 1D), and IFN-γ (results from individual donor PBMCs are shown in Supplemental Figure 1A; supplemental material available online with this article; https://doi.org/10.1172/JCI185942DS1), but not IL-4 and IL-22 (Table 1) secretion from phytohaemagglutinin -stimulated human PBMCs. IMU-935 does not inhibit RORα and RORβ, 2 conserved members of the ROR family of nuclear receptors (data not shown), indicating its high selectivity for RORγt (for more information about IMU-935 identification, see Supplemental Methods), toxicity (36, 37), and specificity.

To determine how IMU-935 binds to RORγt, a cocrystal structure of IMU-935 and the ligand-binding domain (LBD) of RORγt was resolved at the resolution of 2 Å (Figure 1E, left). The structure of IMU-935 in complex with RORγt encompasses 15 α-helices (H) and 2 β-sheets (S). IMU-935 binds to RORγt via a pocket formed by H4, H6, H8, and S1. The amino acids of RORγt in close contact (<4 Å) with IMU-935 are L324 from H4, V361, M365; A368 from H6, V376 and F377 from S1; and I397 and C393 from H8 (Figure 1E, top right). Interestingly, many other RORγt inhibitors, such as compound-1 (referred to here as Cpd-1) (33) (Figure 1E), PF-06747711 (38), AZD-0284 (39), Cpd-1 (an identically named but different compound that we refer to hereafter as Cpd-1-PLoS for clarity) (40), BMS-986251 (41), and S18-000003 (42) (Supplemental Figure 1B), also bind to RORγt via the same pocket that accommodates IMU-935. However, the IMU-935 binding site is unique, as indicated by its relatively long distance from the loop between H1 and H2 (X-loop) compared with Cpd-1 (8 Å for IMU-935 vs. 4 Å for Cpd-1) (Figure 1E, bottom right); PF-06747711 (3.8 Å); AZD-0284 (3.7 Å); Cpd-1-PLoS (4.5 Å); BMS-986251 (3.7 Å); and S18-000003 (3.8 Å) (Supplemental Figure 1B). Therefore, although binding to the same pocket, IMU-935 and other RORγt inhibitors interact with RORγt differentially.

### IMU-935 prevents Th17-dependent EAE via inhibiting Th17 differentiation.

RORγt regulates the differentiation and function of Th17 cells that contribute to autoimmune diseases. We thus investigated the effects of IMU-935 on RORγt-dependent Th17 function (12, 18). IMU-935 (500 nM) had no effects on general CD4^+^ T cell activation, including proliferation, apoptosis, and production of IFN-γ, TNF-α, and IL-2 (Supplemental Figure 2A). Although there were minor effects on proliferation, IMU-935 had no effects on activated CD8^+^ T cell apoptosis and production of IFN-γ, TNF-α, and IL-2 cytokines (Supplemental Figure 2B), suggesting IMU-935 does not generally affect T cell activation.

**Figure 2:**
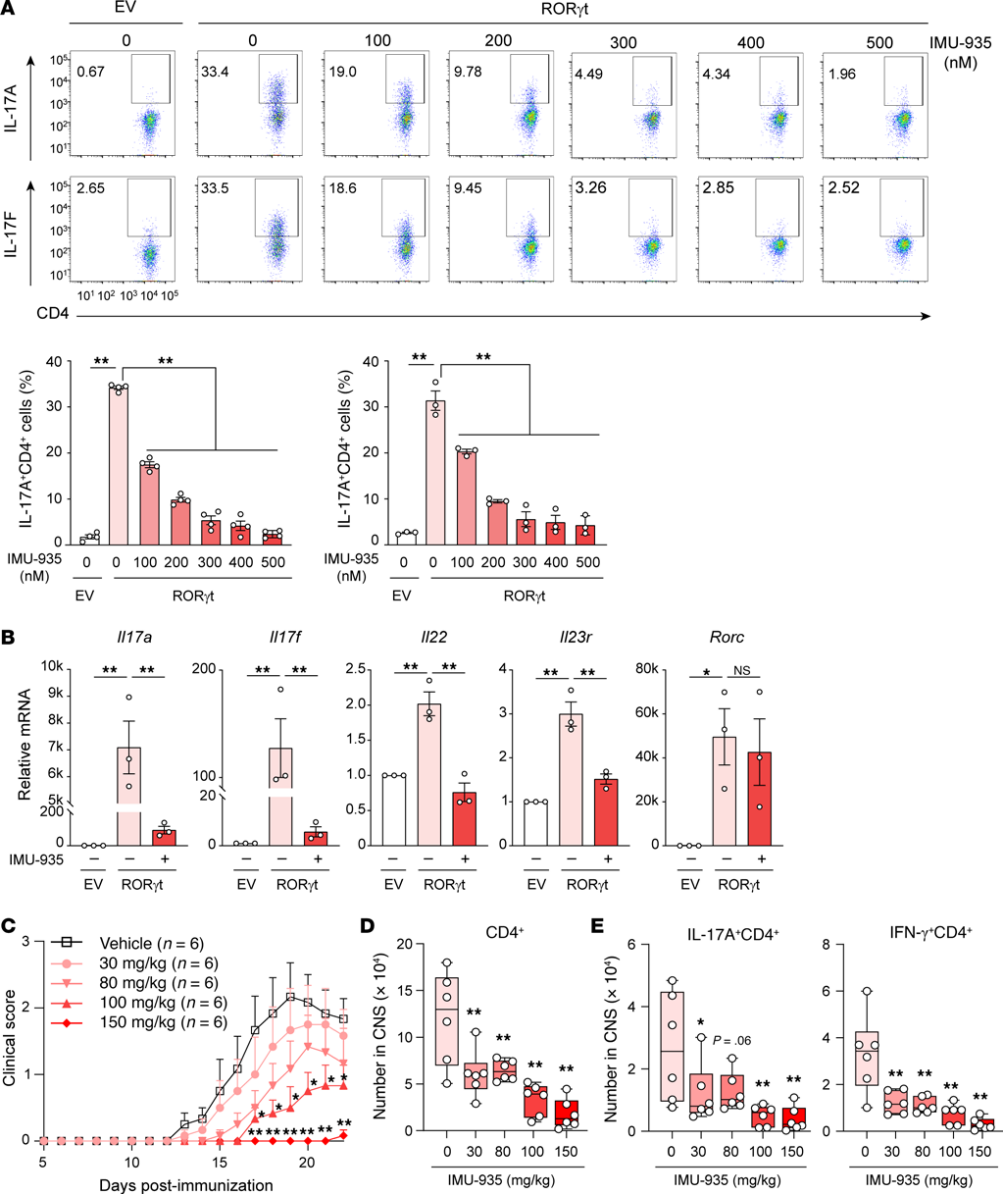
IMU-935 prevents Th17-dependent EAE via inhibiting Th17 differentiation. (**A**) Flow cytometric analysis of IL-17A^+^ or IL-17F^+^ cells among *RORγt^–/–^* CD4^+^ T cells transduced with retrovirus expressing GFP alone (EV) or with WT RORγt and polarized under Th17 conditions for 60 hours with or without IMU-935. Numbers are the percentages of cells in gated areas throughout, IL-17A^+^ (*n* = 4/group, from 4 experiments) and IL-17F^+^ (*n* = 3/group, from 3 experiments) CD4^+^ T cells. (**B**) qRT-PCR analysis of indicated Th17 signature gene expression among differentiated Th17 cells without (–; pink) or with (+; red) 500 nM IMU-935 shown in **A** (*n* = 3/group, from 3 experiments). (**C**) Clinical score of EAE among EAE-induced mice (*n* = 6/group) and treated with vehicle or indicated concentrations of IMU-935 daily for the duration of the experiment. (**D**) Absolute number of indicated lymphocytes recovered from the CNS of the mice described in **C**. (**E**) Numbers of CD4^+^ T cells producing indicated cytokines in the CNS of the mice described in **C**. Data were assessed by 1-way ANOVA with Dunnett’s post hoc test (**A**, **B**, **D** and **E**) or 2-tailed Student’s *t* test (**C**). *P < 0.05; **P < 0.01.

Because RORγt directs Th17 differentiation (12–14), we examined Th17 differentiation in the presence of IMU-935. *RORγt^–/–^* CD4^+^ T cells were defective in Th17 differentiation unless exogenous RORγt was provided via retroviral expression (Figure 2A and see Supplemental Figure 2C for gating strategy), confirming the requirement of RORγt for Th17 differentiation. IMU-935 inhibited Th17 differentiation, including the production of both IL-17A (Figure 2A, top) and IL-17F (Figure 2A, bottom), in a concentration-dependent manner. Similar results were obtained using WT CD4^+^ T cells, in which Th17 differentiation, measured by the frequency of IL-17^+^CD4^+^ T cells, showed a dose-dependent reduction with an IC_50_ of 138.9 nM (Supplemental Figure 2D). Th17 differentiation was almost abrogated in the presence of 300 nM IMU-935, which was not due to induction of CD4^+^ T cell apoptosis (Supplemental Figure 2E). Expression of Th17 signature genes, including *Il17a*, *Il17f*, *Il22*, and *Il23r*, but not *Rorc* (RORγt), was inhibited by IMU-935 (Figure 2B), indicating IMU-935 inhibits the Th17 differentiation program and not just IL-17 production. Overexpression of RORγt stimulated Th17 differentiation in WT CD4^+^ T cells, which was also inhibited by IMU-935 (Supplemental Figure 2F). Furthermore, IMU-935 inhibited Th17 differentiation, but had no obvious effects on Th1 and Th2 differentiation, whereas Treg differentiation was slightly increased (Supplemental Figure 2G).

As a comparison, we tested several known RORγt inhibitors, including Cpd-1 (33) and MRL-871 (43), as well as a RORγt activator, cintirorgon (44). All these RORγt inhibitors, like IMU-935, suppressed, whereas the activator cintirorgon stimulated, Th17 differentiation, including both IL-17A^+^ (Supplemental Figure 2H, left) and IL-17F^+^ (Supplemental Figure 2H, right) cells. Therefore, IMU-935 potently inhibits RORγt function in Th17 differentiation.

Because Th17 cell–mediated immune responses are responsible for EAE (45), the effects of IMU-935 were tested in this mouse model of autoimmunity. As reported (46), Th17-polarized CD4^+^ T cells from *Tg^Tcr2D2^* mice, which recognize oligodendrocyte glycoprotein (MOG_35–55_), induced severe EAE after being adoptively transferred into *Rag1^–/–^* recipients (47) (Supplemental Figure 2I). However, *Tg^Tcr2D2^* CD4^+^ T cells polarized under Th17 conditions in the presence of IMU-935 induced less severe EAE. Consistently, the percentages and absolute numbers of pathogenic IL-17A^+^CD4^+^ and GM-CSF^+^CD4^+^ cells recovered from the CNS were statistically significantly reduced (Supplemental Figure 2, J and K, for gating strategy) of *Rag1^–/–^* recipients reconstituted with IMU-935–treated *Tg^Tcr2D2^* CD4^+^ T cells. Therefore, a brief in vitro treatment with IMU-935 is sufficient to statistically significantly impair CD4^+^ T cell–mediated EAE.

To test the in vivo efficacy of IMU-935, direct EAE was induced in WT mice and treated with vehicle control or different concentrations of IMU-935. Treatment with 30 and 80 mg/kg IMU-935 inhibited EAE development (Figure 2C). IMU-935 reduced CD4^+^ T cell infiltration into the CNS (Figure 2D), including pathogenic IL-17A– and IFN-γ–producing CD4^+^ T cells (Figure 2E), but not CD8^+^, CD19^+^, CD11b^+^ (see gating strategy in Supplemental Figure 2, L and M), IL-17A^+^CD8^+^, or IFN-γ^+^CD8^+^ (Supplemental Figure 2N) cells and Tregs (Supplemental Figure 2O), indicating reduced inflammation and consistency with reduced disease severity. Higher doses of IMU-935, 100 and 150 mg/kg, had a statistically significant therapeutic effect on EAE clinical score and lymphocyte infiltration, including CD8^+^ T and CD19^+^ B cells (Supplemental Figure 2L). At 150 mg/kg, IMU-935 almost completely prevented EAE and also reduced the number of CD8^+^IFN-γ^+^ cells (Supplemental Figure 2N) and Tregs, although the proportion of Tregs was increased in the CNS (Supplemental Figure 2O). A separate experiment with more mice (*n* = 14) also confirmed that 100 mg/kg IMU-935 impaired EAE development and incidence (Supplemental Figure 2P). Therefore, IMU-935 ameliorates Th17-mediated disease severity in EAE by inhibiting Th17 differentiation. This finding indicates its potential value in the clinical treatment of Th17-dependent autoimmune diseases.

### IMU-935 inhibits RORγt target genes critical for Th17 differentiation.

**Figure 3:**
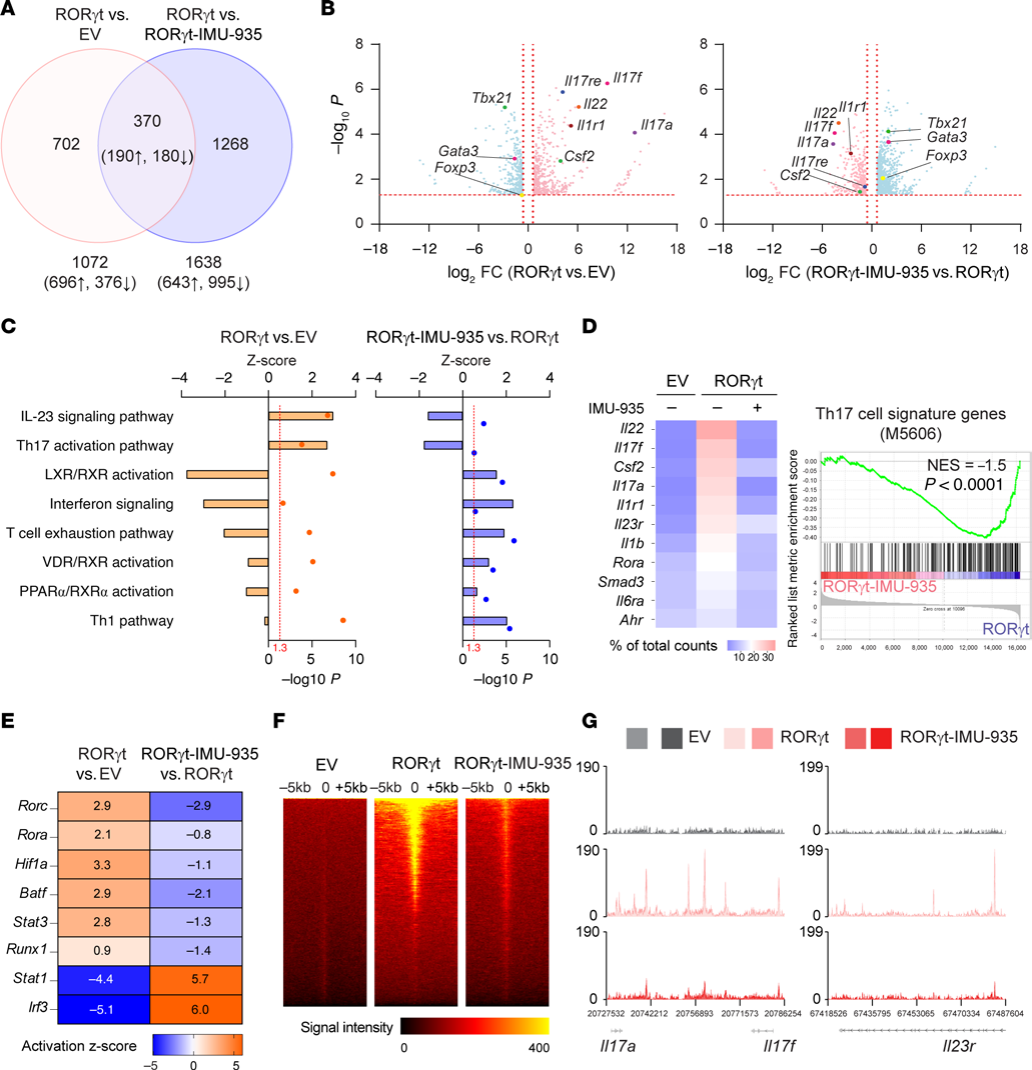
IMU-935 inhibits RORγt target genes critical for Th17 differentiation. (**A**) Venn diagram displaying the number of DEGs (1.5-fold, up or down; *P* < 0.05) identified by RNA-Seq assays in Th17-polarized RORγt-expressing *RORγt^–/–^* CD4^+^ T cells (RORγt throughout) compared with *RORγt^–/–^* cells (EV) and in IMU-935–treated (500 nM) compared with nontreated RORγt^+^ CD4^+^ T cells. (**B**) Volcano plots displaying the global gene expression changes. The horizontal dotted line marks *P* = 0.05; the vertical dotted lines mark fold change of ±1.5. (**C**) The horizontal bars denote pathways identified by IPA canonical signaling pathways analysis. Upregulation (*z* > 0) or downregulation (*z* < 0) of the pathway activity in RORγt^+^ versus *RORγt^–/–^* cells (orange) or IMU-935–treated compared with nontreated RORγt^+^ cells (blue). The vertical dotted lines mark *P* = 0.05. (**D**) Heatmap showing Th17 signature gene expression in *RORγt^–/–^* (EV) or RORγt^+^ CD4*^+^* cells treated with IMU-935 or not. Right: The gene set enrichment plot showing enrichment of Th17 genes. Gene expression was normalized by total counts of each gene. Specific Th gene sets were derived from the MSig database. (**E**) Heatmap depicting changes in the activity of the upstream regulators predicted by IPA. Activation *z* score indicated increased (orange; *z* > 0) or decreased (blue; *z* < 0) activity, all *z* score values are labeled within the corresponding cell. (**F**) Genome-wide RORγt-DNA binding signal intensity determined by ChIP-Seq assays near the TSS in *RORγt^–/–^* (EV) or IMU-935–nontreated (–) or treated (+) RORγt^+^ CD4*^+^* cells polarized under Th17 conditions for 3 days. (**G**) RORγt binding peaks at *Il17a*–*Il17f* (left) or *Il23r* (right) gene locus. The results are the representative of overlays from 2 separate experiments. NES, normalized enrichment score.

We next investigated how IMU-935 affects RORγt-regulated gene expression during Th17 differentiation. RNA-Seq was performed using 3 groups of Th17-polarized CD4^+^ T cells: (a) *RORγt^–/–^* CD4^+^ T cells (i.e., empty virus [EV]); (b) *RORγt^–/–^* CD4^+^ T cells retrovirally expressing RORγt (RORγt); and (c) *RORγt^–/–^* CD4^+^ T cells expressing RORγt in the presence of IMU-935 (RORγt–IMU-935). Principal component analysis confirmed distinct gene expression patterns within each group (Supplemental Figure 3A). Comparison of transcriptomes between *RORγt^–/–^* CD4^+^ T cells and *RORγt^–/–^* CD4^+^ T cells expressing RORγt identified 1072 differentially expressed genes (DEGs) (Figure 3A) as RORγt-regulated genes. Further comparison between *RORγt^–/–^* CD4^+^ T cells expressing RORγt with and without IMU-935 revealed 1638 DEGs as IMU-935–regulated genes. Cross-examining these DEGs, 370 genes were found to be regulated by both RORγt and IMU-935. Consistent with previous reports (48, 49), RORγt upregulated key genes critical for Th17 differentiation, such as *Il17a* and *Il17f* (Figure 3B), which were downregulated by IMU-935 (Figure 3B), supporting the idea that IMU-935 inhibits Th17 differentiation via targeting RORγt. Interestingly, RORγt expression downregulated genes critical for Th1 (*Tbx21*), Th2 (*Gata3*), and Treg (*Foxp3*) differentiation (Figure 3B), aligning with the reciprocal inhibition among T helper cell lineages (50). In contrast, addition of IMU-935 alongside RORγt expression upregulated *Tbx21*, *Gata3*, and *Foxp3* (Figure 3B), likely due to its inhibition of RORγt-dependent Th17 differentiation, reciprocally promoting the expression of Th1, Th2, and Treg lineage genes.

The 370 genes regulated by both RORγt and IMU-935 were then subjected to ingenuity pathway analysis (IPA). The RORγt-stimulated pathways, including IL-23 signaling and Th17 activation pathways, were inhibited by IMU-935 (Figure 3C). Additionally, IMU-935 suppressed the glycolysis pathway critical for Th17 differentiation (51) (Supplemental Figure 3B). Particularly, genes crucial for Th17 activation pathway were upregulated by RORγt expression, whereas they were downregulated in the case of concomitant IMU-935 administration (Figure 3D). Conversely, genes critical for the Th1/Th2 pathway were downregulated by RORγt expression and upregulated in the presence of IMU-935 (Supplemental Figure 3C). IMU-935 thus inhibits RORγt-dependent pathways essential for Th17 differentiation. Notably, a signaling gene network critical for Th17 differentiation was stimulated by RORγt expression (Supplemental Figure 3D, left) whereas this network was inhibited by IMU-935 (Supplemental Figure 3D, right).

Next, we subjected DEGs to IPA upstream regulator analysis to predict which transcription factors are responsible for altered gene expression patterns (Figure 3E). The top candidate was *Rorc* (RORγt), whose activity was greatly upregulated by RORγt expression in *RORγt^–/–^* CD4^+^ T cells and statistically significantly downregulated by IMU-935 treatment. Similarly, *Rora*, *Hif1a*, *Batf*, *Stat3*, and *Runx1*, known to stimulate Th17 differentiation (45, 48), were upregulated alongside with *Rorc* but inhibited by IMU-935. Conversely, *Stat1* and *Irf3*, which negatively regulate Th17 differentiation (52, 53), were inhibited by RORγt expression but stimulated by IMU-935. RNA-Seq data analysis thus supported the notion that IMU-935 inhibits RORγt-regulated pathways and critical gene networks required for Th17 differentiation.

To assess the impact of IMU-935 on RORγt-DNA binding activity, we conducted ChIP-Seq analysis to compare RORγt chromatin occupancy. In *RORγt^–/–^* CD4^+^ T cells, no discernible DNA binding peaks were observed, whereas RORγt-DNA binding peaks were readily detected in *RORγt^–/–^* CD4^+^ T cells expressing RORγt (Figure 3F). Most of the RORγt binding peaks were located near transcriptional starting sites (TSSs), with fewer detected in introns, 5′UTR, 3′UTR, and coding sequence (CDS) (Supplemental Figure 3E), confirming the function of RORγt in regulating gene expression. Notably, IMU-935 markedly reduced RORγt binding signals (Figure 3F), including *Il17a*, *Il17f*, and *Il23r* loci (Figure 3G); these findings were further validated by individual ChIP assays (Supplemental Figure 3F). The identified RORγt-DNA binding peaks at *Il17a*, *Il17f*, and *Il23r* loci overlapped with published data (48) (Supplemental Figure 3G), affirming the accuracy of our ChIP-Seq assays. Furthermore, the enhanced RORγt-DNA binding signals correlated with elevated expression of key genes crucial for Th17 differentiation, including *Il22, Il17a*, *Il17f,* and *Il23r* (Supplemental Figure 3H). Conversely, decreased expression of these Th17 genes due to IMU-935 treatment coincided with reduced RORγt-DNA binding at their loci. Therefore, IMU-935 inhibits RORγt-DNA binding activity at the critical Th17 gene loci, thereby suppressing their expression and ultimately inhibiting Th17 differentiation.

We then compared IMU-935–regulated gene expression patterns with publicly available datasets. The top matched dataset (Table 2) was Th17 versus Th0, which had a very high negative similarity score (*z* score = –66). This indicates that upregulated genes in Th17 cells were downregulated by IMU-935. The other 3 matches with a high negative similarity score were systematic lupus erythematosus, psoriasis, and multiple sclerosis versus the healthy condition, suggesting genes upregulated in these 3 Th17-mediated autoimmune diseases are inhibited by IMU-935. Additionally, datasets with high positive similarity include those for medically treated psoriasis and multiple sclerosis versus the healthy condition, indicating IMU-935 targets the same genes affected by the approved medical treatments for these autoimmune diseases. These findings support the clinical application of IMU-935 in treating Th17-mediated autoimmunity. Other matches with high positive similarity score included treated acute myeloid leukemia, clinically isolated syndrome, systematic lupus erythematosus, and type 1 diabetes versus the healthy condition, suggesting the potential of IMU-935 in treating these diseases.

### IMU-935 does not affect thymocyte development and survival.

**Figure 4:**
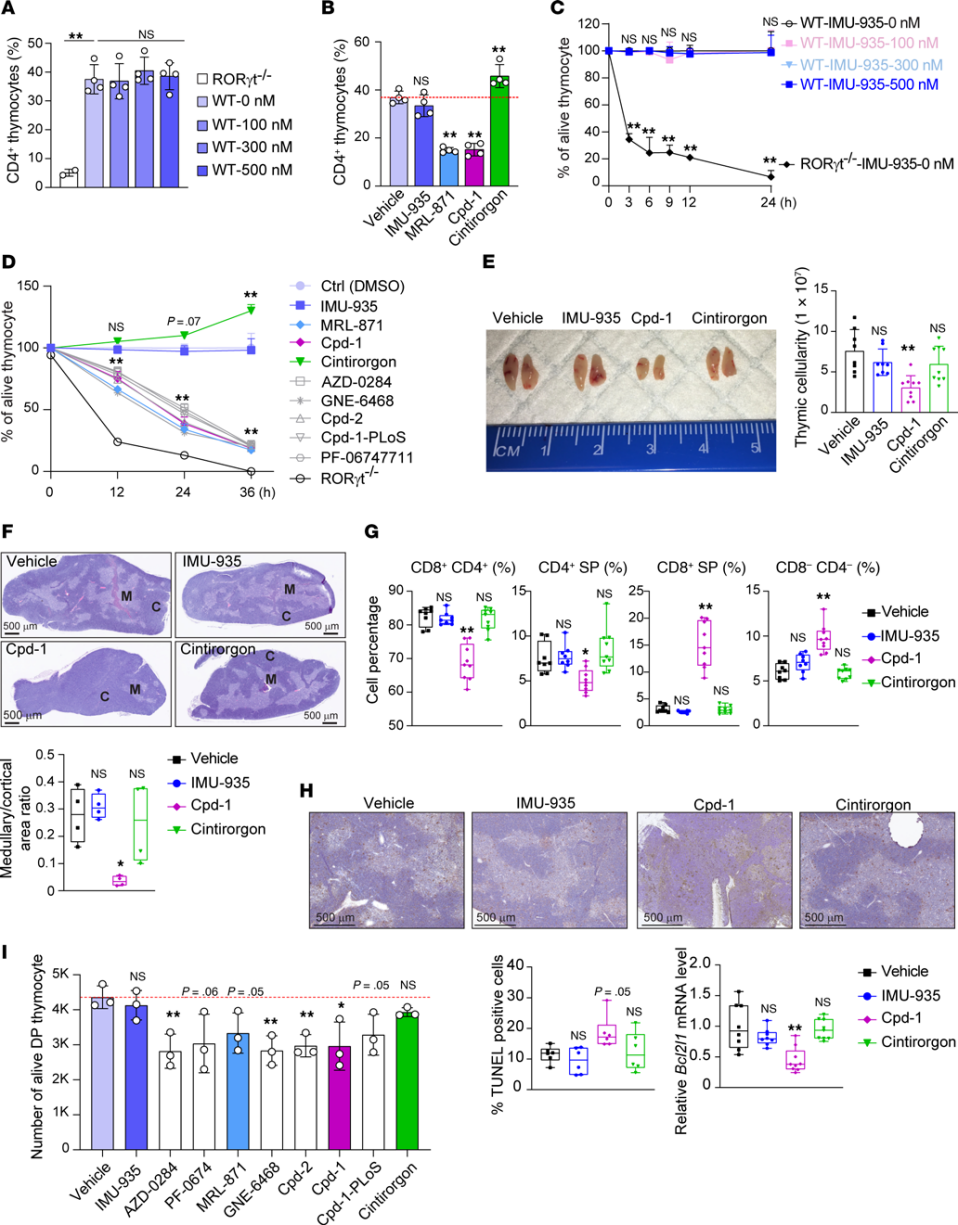
IMU-935 does not affect thymocyte development and survival. (**A** and **B**) Flow cytometric analysis of CD4^+^ and CD8^+^ thymocytes ex vivo developed for 3 days from sorted *RORγt^–/–^* CD4*^–^*CD8*^–^* or WT CD4*^–^*CD8*^–^* in the presence of different concentrations of IMU-935 (**A**) (*n* = 2–4/group, from 4 experiments) or RORγt modulators (**B**) (1 μM; *n* = 4/group, from 4 experiments). Percentages of CD4^+^ plus CD4^+^CD8^+^ T cells among live Thy1.2^+^ thymocytes are shown. (**C** and **D**) Percentages of live cells among CD4^+^CD8^+^ thymocytes relative to the vehicle control different times after culture in the presence of indicated concentrations of IMU-935 (**C**) or RORγt modulators (**D**) (1 μM; *n* = 3/group, from 1 experiment). Thymocytes from *RORγt^–/–^* mice were used as a control. (**E**) Picture and cellularity of the thymus from mice (*n* = 8–9/group; *n* = 8 for vehicle and IMU-935 groups, and *n* = 9 for Cpd-1 and cintirorgon groups) treated with vehicle or indicated RORγt inhibitors or cintirorgon (100 mg/kg orally, twice daily) for 28 days. (**F**) H&E staining (scale bar: 500 μm) of the thymus from mice treated as described in **E**. M, medullar; C, cortex. Bottom: The ratio of the medullary/cortical region. (**G**) Flow cytometric analysis of CD4 and CD8 in the thymocytes from mice treated as described in **E**. (**H**) TUNEL staining (top; scale bar: 500 μm) and percentage of TUNEL-positive apoptotic cells in thymus obtained from mouse treated as described in **E**. Bottom right: The qRT-PCR analysis of *Bcl2l1* mRNA. (**I**) Number of alive, human CD4^+^CD8^+^ thymocytes cultured in the presence of indicated RORγt inhibitors (1 μM) for 24 hours (thymocytes collected from 1 donor, *n* = 3 replicates/group). Data were assessed by 1-way ANOVA with Dunnett’s post hoc test (**A**–**H**) or 2-tailed Student’s *t* test (**I**). *P < 0.05; **P < 0.01.

We next investigated whether IMU-935 affects RORγt function in thymocytes first using an in vitro thymocyte differentiation system (54). In this system, CD4^–^CD8^–^ thymocytes from *RORγt^–/–^* mice failed to differentiate into the next developmental stages of CD4^+^CD8^+^ and CD4^+^ cells (Figure 4A and see Supplemental Figure 4A for representative flow cytometry plots), confirming the essential role of RORγt in thymocyte development. Importantly, CD4^–^CD8^–^ thymocytes from WT mice differentiated into CD4^+^CD8^+^ and CD4^+^ cells regardless of IMU-935 presence, indicating IMU-935 does not disrupt thymocyte development.

In contrast to IMU-935, RORγt inhibitors MRL-871 and Cpd-1, but not the RORγt agonist cintirorgon, inhibited differentiation of CD4^+^CD8^+^ and CD4^+^ cells from WT CD4^–^CD8^–^ thymocytes (Figure 4B and see Supplemental Figure 4B for representative flow cytometry plots). The requirement of RORγt for CD4^+^CD8^+^ thymocyte survival explains why CD4^–^CD8^–^ *RORγt^–/–^* thymocytes fail to differentiate (13, 24). CD4^+^CD8^+^ thymocytes from *RORγt^–/–^* mice undergo accelerated apoptosis (Figure 4C and see Supplemental Figure 4C for gating strategy). However, CD4^+^CD8^+^ thymocyte survival was not affected by varying concentrations of IMU-935 (Figure 4C). Notably, even at concentrations of 300 nM and 500 nM, which effectively inhibited RORγt-dependent Th17 differentiation (Figure 2A), IMU-935 did not induce thymocyte apoptosis. *RORγt^–/–^* thymocytes undergo apoptosis due to downregulated antiapoptotic gene *Bcl2l1* (Bcl-xL), and forced expression of *Bcl2l1* rescued *RORγt^–/–^* thymocytes from apoptosis (29). Consistently, *RORγt^–/–^* thymocytes had reduced levels of *Bcl2l1* but not Myc (Supplemental Figure 4D). Importantly, IMU-935 did not affect *Bcl2l1* expression. In contrast, other RORγt inhibitors, including Cpd-1 and MRL-871, statistically significantly increased thymocyte apoptosis, whereas the RORγt agonist cintirorgon enhanced thymocyte survival (Figure 4D). Consistently, Cpd-1 and MRL-871, but not IMU-935, reduced *Bcl2l1* expression, whereas cintirorgon increased it (Supplemental Figure 4E).

We then assessed the effects of IMU-935 on thymocytes in vivo using both short-term and long-term treatment models. In the short-term model, MRL-871 statistically significantly reduced the size (Supplemental Figure 4F, top) and cellularity (Supplemental Figure 4F, bottom) of the thymus after 3 days, as previously reported (43). In contrast, IMU-935 treatment did not notably affect thymic size and cellularity, similar to vehicle and cintirorgon treatments. Histological analysis of thymus indicated that thymus from MRL-871–treated mice, but not IMU-935–treated mice, had lost most medullar regions (Supplemental Figure 4G), resembling the phenotype observed in *RORγt^–/–^* mice (24). Thymocyte subset analysis showed that MRL-871, but not IMU-935 and cintirorgon, greatly reduced the percentage and number of CD4^+^CD8^+^ thymocytes in vivo (Supplemental Figure 4H), and decreased thymocyte survival in vitro (Supplemental Figure 4I). TUNEL staining confirmed increased numbers of apoptotic cells and decreased mRNA levels of antiapoptotic *Bcl2l1* in the thymus from MRL-871– but not from IMU-935– or cintirorgon-treated mice (Supplemental Figure 4J). Thymocytes from *RORγt^–/–^* mice had more cells with >2N DNA, indicating an increase in cells at the DNA synthesis phase of the cell cycle (24). Similarly, thymocytes from MRL-871–, but not IMU-935– or cintirorgon-treated mice, had substantially more cells with >2N DNA (Supplemental Figure 4K). Therefore, IMU-935 did not affect thymocyte development and survival in this short-term treatment model.

In the long-term treatment model, mice were treated twice daily with IMU-935 for 28 days. Long-term treatment with IMU-935 did not affect mouse body weight (Supplemental Figure 4L); thymic size (Figure 4E); thymic cellularity (Figure 4E); medullar/cortex ratio (Figure 4F); percentage (Figure 4G and Supplemental Figure 4M for representative flow cytometry plots) or number (Supplemental Figure 4N) of the CD4^+^CD8^+^ thymocytes; apoptosis (Figure 4H and Supplemental Figure 4O); or percentage of thymocytes with >2N DNA (Supplemental Figure 4P). In contrast, the control, Cpd-1, but not cintirorgon, markedly reduced the thymic size and cellularity (Figure 4E), decreased medullar/cortex regions (Figure 4F), reduced the percentage and number of CD4^+^CD8^+^ thymocytes (Figure 4G and Supplemental Figure 4N) through apoptosis (Figure 4H and Supplemental Figure 4O), and increased proportion of thymocytes with >2N DNA (Supplemental Figure 4P).

Finally, we evaluated effects of various RORγt inhibitors on human CD4^+^CD8^+^ thymocyte survival (Figure 4I). MRL-871 and Cpd-1, but not IMU-935, statistically significantly reduced the number of live human CD4^+^CD8^+^ thymocytes. Overall, IMU-935, which inhibits RORγt-dependent Th17 differentiation, does not affect RORγt-dependent thymocyte development and survival.

### IMU-935 does not affect RORγt target genes critical for thymocyte development and survival.

**Figure 5:**
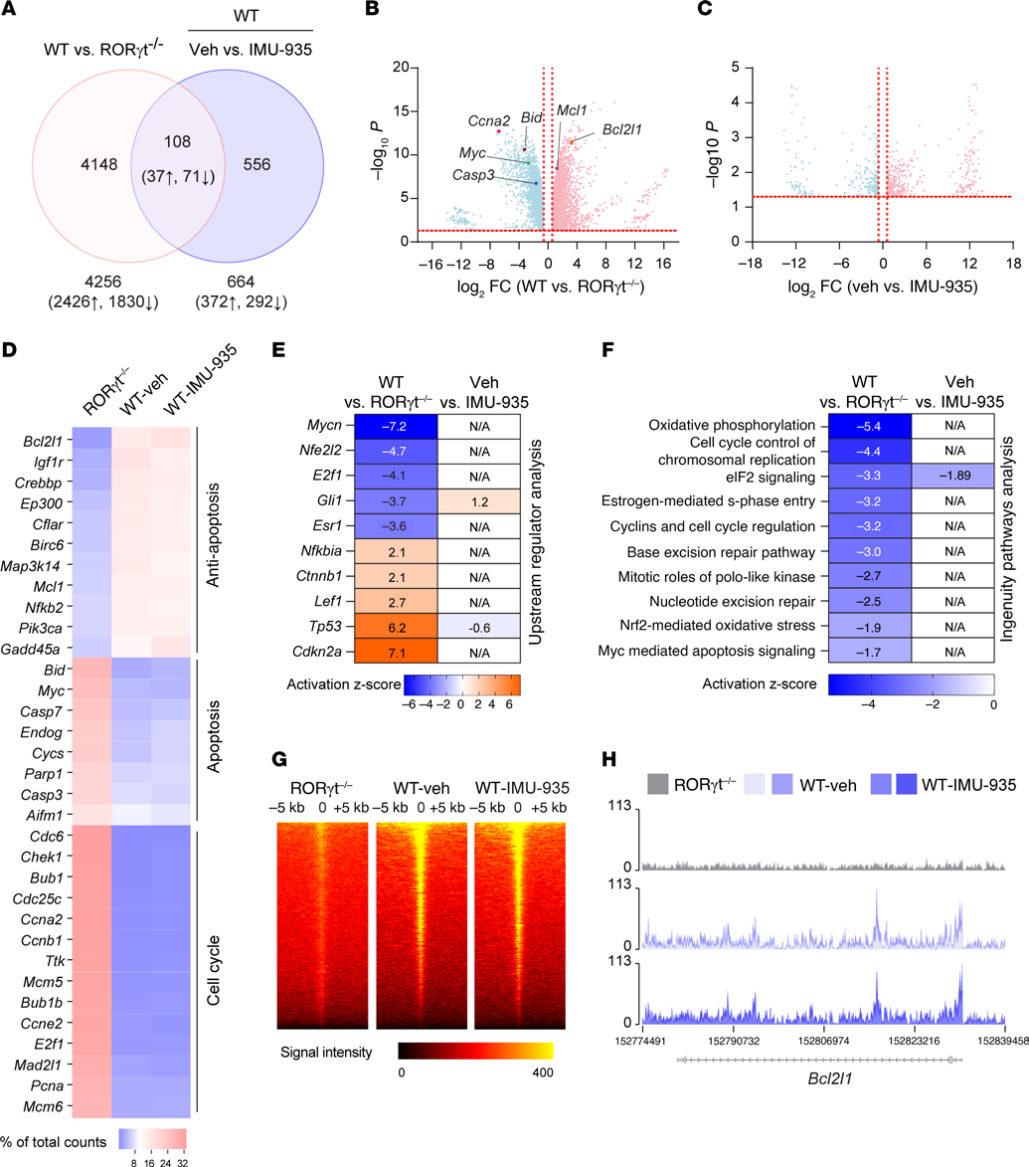
IMU-935 does not affect genes critical for thymocyte survival. (**A**) Venn diagram displaying the number of DEGs (1.5-fold, up or down; *P* < 0.05) identified by RNA-Seq assays in WT versus *RORγt^–/–^* thymocytes and in WT thymocytes treated with IMU-935 (1 μM) or not. (**B** and **C**) Volcano plots displaying the global gene expression. The horizontal dotted line marks *P* = 0.05, the vertical dotted lines mark fold change of ±1.5. (**D**) Heatmap showing antiapoptotic, apoptotic, and cell cycle gene expression in *RORγt^–/–^* or IMU-935–nontreated (WT-vehicle control) or treated (WT–IMU-935) thymocytes. Gene expression was normalized by total counts of each gene. (**E**) Heatmap depicting changes in the activity of upstream regulators predicted by IPA. Activation *z* score indicates increased (orange; *z* > 0) or decreased (blue; *z* < 0) activity. All *z* score values are labeled within the corresponding cell. Genes shown are known to regulate apoptosis (*Mycn*, *E2f1*, *Esr1*), survival (*Nfkbia*, *TP53*), cell cycle (*Gli1*, *Cdkn2a*), oxidative stress (*Nfe2l2*), and thymocyte development (*Ctnnb1*, *Lef1*). (**F**) Heatmap showing the activity of the pathways determined by IPA. Pathway activity was obtained by comparing WT and *RORγt^–/–^* thymocytes (left column) and IMU-935–nontreated versus -treated WT thymocytes (right column). N/A indicates pathway status is not predictable due to the small number of genes affected by IMU-935 treatment in thymocyte RNA-Seq. (**G**) Genome-wide RORγt-DNA binding signal intensity determined by ChIP-Seq assays near the TSS in *RORγt^–/–^* or WT (RORγt^+^) or WT–IMU-935 thymocytes. (**H**) RORγt binding peaks at the *Bcl2l1* locus. The results are the representative of overlays from 2 separate experiments. Veh, vehicle.

To understand the impact of IMU-935 on RORγt-dependent gene expression in thymocytes, we performed RNA-Seq analysis in following 3 groups: (a) *RORγt^–/–^* thymocytes; (b) WT thymocytes treated with vehicle control (WT-Veh); and (c) WT thymocytes treated with IMU-935 (WT–IMU-935). Based on principal component analysis, *RORγt^–/–^* group clearly separated from WT-Veh and WT–IMU-935 groups, which showed similar gene expression patterns (Supplemental Figure 5A). Comparing the transcriptomes between *RORγt^–/–^* and WT thymocytes, we identified 4256 DEGs (Figure 5A) considered to be RORγt-regulated genes. *RORγt^–/–^* thymocytes had greatly downregulated antiapoptotic genes, including *Bcl2l1* and *Mcl1* (Figure 5, B and D), whereas they upregulated apoptotic genes, such as *Bid* and *Casp3* (Figure 5, B and D), which is consistent with the apoptotic phenotype of *RORγt^–/–^* thymocytes. In the comparison of the transcriptomes of WT thymocytes with or without IMU-935, we identified 664 DEGs as IMU-935–regulated genes in thymocytes (Figure 5A). Cross-examining these DEGs, 108 genes were found to be regulated by both RORγt and IMU-935 (Supplemental Figure 5B). Pathway analysis showed that these 108 genes were not directly related to apoptotic function (Supplemental Figure 5C). Consistently, IMU-935 treatment did not affect the expression of anti-apoptotic and apoptotic genes regulated by RORγt, as illustrated by their equivalent expression in WT thymocytes in the presence and absence of IMU-935 (Figure 5C and 5D).

We then subjected RNA-Seq data from *RORγt^–/–^* thymocytes to IPA upstream regulator analysis (Figure 5E), pathway analysis (Figure 5F), and gene network analysis (Supplemental Figure 5D). These analyses identified RORγt-dependent upstream regulators, pathways, and gene networks critical (Supplemental Figure 5D, left) for cell survival, cell cycle, and thymocyte development, aligning with RORγt’s known functions in thymocytes. Importantly, the activity of these upstream regulators (Figure 5E), pathways (Figure 5F), and gene networks (Supplemental Figure 5D, right) were mostly unaffected by IMU-935, confirming that IMU-935 does not interfere with RORγt-dependent functions in thymocytes.

ChIP-Seq analysis was performed to determine RORγt chromatin occupancy. *RORγt^–/–^* thymocytes had no obvious RORγt binding peaks (Figure 5G, left), whereas strong DNA binding signals were detected in WT thymocytes (Figure 5G, middle). Most RORγt-DNA binding peaks were identified near TSS, whereas there were much fewer peaks in introns, 5′UTR, 3′UTR, and CDS (Supplemental Figure 5E). Unlike greatly reduced RORγt binding peak signals caused by IMU-935 in Th17 cells (Figure 3F), RORγt binding peak signals were apparently not affected by IMU-935 in thymocytes (Figure 5G, right). Notably, RORγt binding to the *Bcl2l1* locus was not impaired by IMU-935 (Figure 5H), which was further confirmed by individual ChIP assay (Supplemental Figure 5F). Among 864 genes that had RORγt binding peaks, 35 genes were regulated by IMU-935 (Supplemental Figure 5G). IPA indicated these 35 genes were not directly related to apoptosis (Supplemental Figure 5H), confirming that IMU-935 does not alter the expression of RORγt-dependent genes critical for cell survival. Furthermore, crossing examining RNA-Seq and ChIP-Seq data, we found that RORγt-stimulated expression of genes critical for cell survival correlated well with increased RORγt binding to those gene loci (Supplemental Figure 5I). Conversely, IMU-935 did not affect the expression of these cell survival genes, nor did it affect RORγt binding to the corresponding gene loci (Supplemental Figure 5I). In conclusion, IMU-935 does not alter overall RORγt-DNA binding activity or the expression of RORγt-dependent genes critical for survival and development of thymocytes.

### IMU-935 preferentially disrupts the interaction between RORγt and cofactor CBFβ in Th17 cells but not in thymocytes, due to different levels of RUNX1 competing with RORγt for binding to CBFβ in these cells.

**Figure 6:**
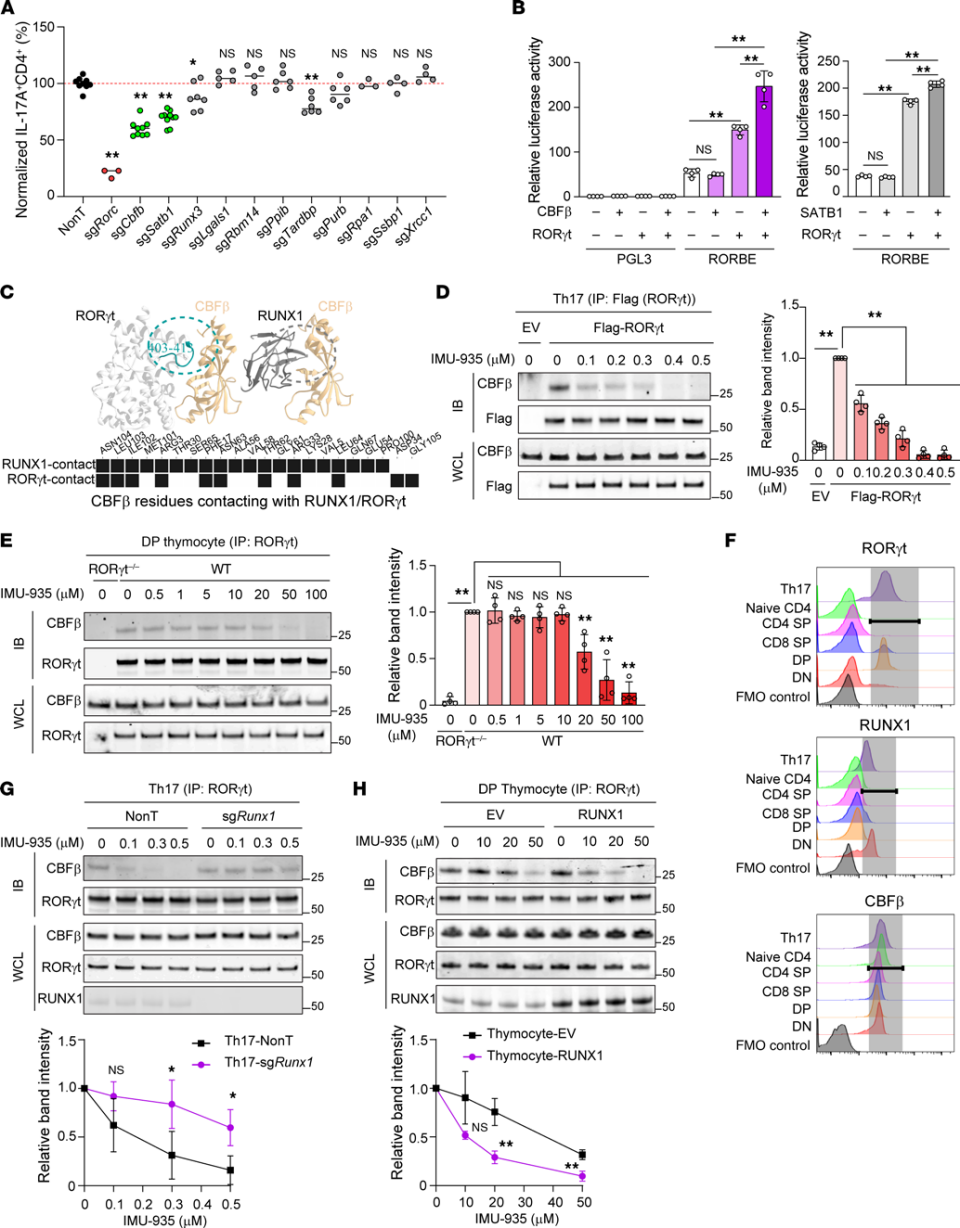
IMU-935 inhibits RORγt’s interaction with cofactor CBFβ in Th17 cells but not in thymocytes. (**A**) Changes in Th17 differentiation after knockout of indicated genes, relative to nontargeting group (NonT; 100%) in Cas9-expressing CD4^+^ T cells (*n* = 3–10/group from 3 pooled independent experiments). (**B**) Relative luciferase activity from a promoterless control (pGL3) or RORγt reporter (RORBE) in HEK293T cells transfected with indicated expression plasmids for 24 hours. (**C**) A visualization of the computer-predicted interaction between RORγt and CBFβ (top left) or RUNX1 and CBFβ (top right). Dashed circle highlights the interaction interface between indicated proteins. The cyan fragment indicates the RORγt amino acids 403–413 critical for binding to CBFβ. The black box in the bottom panel indicates the amino acids with >40% contact frequency with CBFβ; the white box indicates <40% contact frequency. (**D** and **E**) IP analysis of the RORγt-CBFβ interaction in *RORγt^–/–^* CD4^+^ T cells expressing GFP (EV) or with Flag-RORγt polarized in Th17 conditions (**D**) in *RORγt^–/–^* or WT thymocytes (**E**) and treated with indicated concentrations of IMU-935. IP with anti–Flag-RORγt (**D**) or anti-RORγt (**E**) antibody and immunoblot with anti-CBFβ antibody. Input was analyzed by Western blot (bottom 2 panels). Right: The relative intensity of immunoprecipitated CBFβ band. (**F**) Flow cytometric analysis of RORγt (top), RUNX1 (middle), and CBFβ (bottom) levels in differentiated Th17 cells, peripheral naive CD4^+^ T cells, and different subsets of thymocytes. (**G** and **H**) IP analysis of the RORγt-CBFβ interaction in control (NonT) or *Runx1*-deleted Th17 cells (sg*Runx1*) (**G**) or thymocytes expressing EV or RUNX1 (**H**) treated with indicated concentrations of IMU-935, similar to what is described in **D** and **E**. Data represent 2 (**F**), 3 (**H**), or 4 (**B**, **D**, **E**, and **G**) independent experiments. Data were assessed by 1-way ANOVA with Dunnett’s (**A**, **D**, and **E**), Tukey’s (**B**) post hoc test, or 2-tailed Student’s *t* test (**G** and **H**). *P < 0.05; **P < 0.01.

To understand how IMU-935 inhibits RORγt-mediated Th17 differentiation, we used mass spectrometry to analyze the proteins that coimmunoprecipitated with RORγt. We identified 296 RORγt-associated proteins in RORγt^+^ Th17 cells without IMU-935 treatment. Among these, we excluded 180 proteins that were also detected in *RORγt^–/–^* cells and/or IMU-935–treated RORγt^+^ Th17 cells (Supplemental Figure 6A, left), because these were either nonspecific RORγt binding proteins or their interactions with RORγt were not regulated by IMU-935. This left 116 proteins bound to RORγt in the absence but not in the presence of IMU-935. Given RORγt’s role as a transcription factor, we focused on 11 known transcriptional regulators (Supplemental Figure 6A, right). These 11 proteins were individually knocked out in CD4^+^ T cells obtained from Cas9-expressing mice (55). The deletion of *Cbfb*, *Satb1*, or *Rorc* (positive control) statistically significantly impaired Th17 differentiation (Figure 6A and see Supplemental Figure 6B for representative flow cytometry plots), highlighting their roles in this process. *Cbfb* encodes CBFβ, identified 3 decades ago as the RUNX1 binding protein (56–59). Because RUNX is the only known binding partner for CBFβ and is a non–DNA-binding protein (60), CBFβ is believed to regulate gene expression by interacting with RUNX. Indeed, CBFβ together with RUNX1 greatly stimulated a RUNX1-luciferase reporter gene compared with CBFβ alone (Supplemental Figure 6C). We tested whether CBFβ affects RORγt transcriptional activity. CBFβ together with RORγt, but not alone, greatly stimulated an RORγt-luciferase reporter gene (Figure 6B, left), and IMU-935 inhibited CBFβ-stimulated RORγt activity (Supplemental Figure 6D, left) but not RUNX1 activity (Supplemental Figure 6D, right), suggesting RORγt recruits CBFβ to stimulate transcription, which is inhibited by IMU-935. In contrast to CBFβ, SATB1 only had a minor effect on RORγt reporter activity (Figure 6B, right). ChIP assay detected that CBFβ and, to a much lesser extent, SATB1 bound to the same sites as RORγt at *Il17a* (Supplemental Figure 6E, left) and *Il23r* loci in vivo (Supplemental Figure 6E right), which was inhibited by IMU-935. This confirms that CBFβ, together with RORγt, binds and regulates the RORγt target genes critical for Th17 differentiation. Additionally, CBFβ-RORγt complexes were detected by IP in Th17 cells in the absence, but not presence, of 500 nM IMU-935 (Supplemental Figure 6F, left), confirming that CBFβ interacts with RORγt, and IMU-935 disrupts their interaction in Th17 cells. CBFβ-RORγt complexes were also detected in thymocytes (Supplemental Figure 6F, right), but their interaction in thymocytes was not affected by 500 nM IMU-935 that was sufficient to disrupt the CBFβ-RORγt interaction in Th17 cells. Our analysis of the RORγt-SATB1 interaction was inconclusive, partially due to high levels of background immunoprecipitated complexes, particularly in thymocytes (Supplemental Figure 6F, right). Given that SATB1 does not bind (Supplemental Figure 6F) and stimulate (Figure 6B) RORγt activity as strongly as CBFβ, we focused on the CBFβ-RORγt interaction in the following study.

Because RUNX1 also interacts with CBFβ, we wanted to determine whether RUNX1 competes with RORγt for binding to CBFβ. We first performed computational protein-protein docking simulation analysis between CBFβ and RORγt (Figure 6C, top left). The simulated CBFβ and RORγt interaction has striking similarity to the resolved crystal structure of CBFβ and RUNX1 complexes (Protein Data Bank identifier 3WTS) (Figure 6C, top right). Specifically, CBFβ uses the same interface to interact with both RUNX1 and RORγt. To further verify this, we performed molecular dynamics simulations to determine the contact frequency between CBFβ and RUNX1 or RORγt. Indeed, CBFβ uses many of the same amino acids to contact both RUNX1 and RORγt with high frequency (Figure 6C, bottom), strongly supporting the notion that both molecules physically compete for binding to CBFβ. Furthermore, increasing the amount of RUNX1 gradually decreased CBFβ-RORγt complexes detected by IP (Supplemental Figure 6G) and reduced CBFβ-stimulated RORγt transcription activity (Supplemental Figure 6H, left). Conversely, RORγt also inhibited RUNX1- and CBFβ-stimulated RUNX reporter activity (Supplemental Figure 6H, right), confirming the competitive interaction of CBFβ with both RUNX1 and RORγt. Lastly, in contrast to IMU-935, which disrupted the RORγt-CBFβ interaction in Th17 cells but not in thymocytes (Supplemental Figure 6F), the RORγt inhibitors Cpd-1 and MRL-871 disrupted the RORγt-CBFβ interaction in both Th17 cells and thymocytes (Supplemental Figure 6I). Therefore, as with Cpd-1 and MRL-871, IMU-935 disrupts the CBFβ-RORγt interaction and RORγt-dependent Th17 differentiation. In contrast to Cpd-1 and MRL-871, IMU-935 does not affect the CBFβ-RORγt interaction or impact thymocyte development and survival.

To understand the differential effect of IMU-935 on the CBFβ-RORγt interaction in Th17 cells and thymocytes, we tested a wide range of IMU-935 concentrations on the CBFβ-RORγt interaction in these 2 types of cells. Consistent with our previous findings, 100 nM (0.1 μM) IMU-935, which started to inhibit Th17 differentiation (Figure 2A), also impaired the RORγt-CBFβ interaction in Th17 cells (Figure 6D). And 0.5 μM IMU-935, which completely inhibited Th17 differentiation (Figure 2, A and B) and disrupted the RORγt-CBFβ interaction in Th17 cells (Supplemental Figure 6F), had no effect on this interaction in thymocytes (Figure 6E) or on thymocyte survival (Figure 4, A and C). Disruption of the RORγt-CBFβ interaction in thymocytes was only observed at much higher concentrations of IMU-935, obviously starting at 20 μM (Figure 6E) — 200-fold higher than that required in Th17 cells. These results suggest the RORγt-CBFβ interaction is cell-context dependent.

Given our evidence that RUNX1 competes with RORγt for binding to CBFβ, we next investigated whether the presence of competing RUNX1 influences the RORγt-CBFβ interaction. We first analyzed protein levels of RUNX1, RORγt, and CBFβ in both thymocytes and Th17 cells. Consistent with previous studies (12, 24, 61), RORγt expression was at the highest levels in DP thymocytes and Th17 cells (Figure 6F), where it regulates thymocyte survival and Th17 differentiation separately. RORγt levels were low in DN, CD4^+^, or CD8^+^ single-positive thymocytes and peripheral naive CD4^+^ T cells. However, RUNX1 levels in DP thymocytes was among the lowest, whereas the highest were in Th17 cells (Figure 6F). CBFβ levels remained relatively stable across all thymocyte stages and Th17 cells (Figure 6F). High levels of RUNX1 in Th17 cells compete with RORγt for binding to CBFβ, presumably weakening the RORγt-CBFβ interaction and thereby requiring a lower concentration of IMU-935 to disrupt the interaction. In contrast, low levels of competing RUNX1 in thymocytes likely favor RORγt-CBFβ interaction, thus requiring a much higher concentration of IMU-935 to achieve the disruption. Indeed, deletion of RUNX1 in Th17 cells substantially increased the IMU-935 concentration required to disrupt the RORγt-CBFβ interaction (Figure 6G). Conversely, overexpression of RUNX1 in thymocytes significantly reduced the IMU-935 concentration required to disrupt this interaction (Figure 6H). Altogether, the differential effects of IMU-935 in Th17 cells and thymocytes result from the markedly different concentrations needed to disrupt the RORγt-CBFβ interaction. Varying levels of RUNX1, which competes with RORγt for binding to CBFβ, contribute to the differential effects of IMU-935 on the RORγt-CBFβ interaction in these 2 types of cells.

### The interaction between RORγt and CBFβ is essential for Th17 differentiation and thymocyte development.

**Figure 7:**
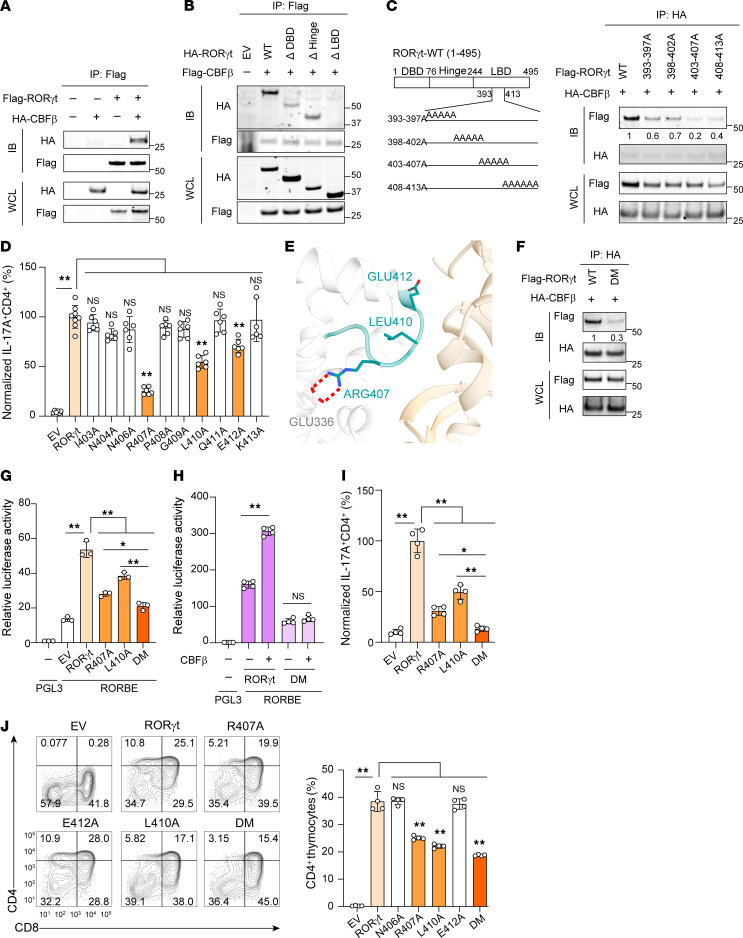
The interaction between RORγt and CBFβ is essential for Th17 differentiation and thymocyte development. (**A**–**C**) IP analysis of the RORγt-CBFβ interaction in HEK293T cells with indicated expression plasmids for WT (**A**), deletion mutation (**B**), or alanine scanning mutation (**C**) RORγt. ΔDBD, DNA binding domain-deleted RORγt; ΔHinge, hinge-deleted RORγt; ΔLBD, Lihand-binding domain-deleted RORγt. (**D**) The percentage of Th17 differentiation activity supported by the RORγt with the indicated point mutation expressed in *RORγt^–/–^* CD4^+^ T cells relative to those expressing WT RORγt (defined as 100%). *RORγt^–/–^* CD4^+^ T cells expressing GFP alone (EV) or with WT RORγt or RORγt with the indicated single amino acid mutated to alanine were polarized under Th17 conditions. (**E**) Visualization of computer-predicted interaction between RORγt and CBFβ. The cyan fragment indicates RORγt amino acids 403–413. R407, L410, and E412 of RORγt critical for binding to CBFβ are indicated. Red dashed lines indicate the ionic lock between R407 and E336. (**F**) IP analysis of CBFγ interaction with RORβt or R407A/L410A DM. (**G** and **H**) Relative luciferase activity from HEK293T cells transfected with pGL3 control or RORγt reporter (RORBE) together with plasmids expressing WT or RORγt with indicated mutation (**G**) with or without CBFβ (**H**). (**I**) The percentage of Th17 differentiation activity supported by the RORγt with indicated mutation expressed in *RORγt^–/–^* CD4^+^ T cells relative to those expressing WT RORγt (defined as 100%). (**J**) Flow cytometric analysis of CD4^+^ and CD8^+^ thymocytes ex vivo developed for 3 days from sorted *RORγt^–/–^* CD4^–^CD8^–^ transduced with WT or RORγt with indicated mutation. Right: The summary of the percentages of CD4^+^ plus CD4^+^CD8^+^ T cells among live Thy1.2^+^ cells. Data represent 3 (**D** and **G**) or 4 (**H**–**J**) independent experiments; representative Western blots are shown from 3 independent experiments (**A**–**C**, and **F**). Data were analyzed by 1-way ANOVA with Dunnett’s (**D**) or Tukey’s (**G**–**J**) post hoc test. *P < 0.05; **P < 0.01. WCL, whole-cell lysate.

To elucidate the function of the RORγt-CBFβ interaction in Th17 cells and thymocytes, we performed systematic mutagenesis of RORγt to identify RORγt mutants that cannot interact with CBFβ. Initially, the RORγt-CBFβ interaction was confirmed in overexpressing HEK293T cells (Figure 7A). Deletion of the LBD, but not the DNA binding domain or hinge domain, completely disrupted the RORγt-CBFβ interaction (Figure 7B). By creating a larger region deletion within LBD, we narrowed down the CBFβ binding region to amino acids 393–413 of RORγt (Supplemental Figure 7, A and B). Further alanine scanning analysis of every 5 amino acids within the 393–413 region identified critical binding sites for CBFβ as amino acids 403–413 of RORγt (Figure 7C), because substitution of 5 consecutive amino acids between 403–407 or 408–413 caused the greatest impairment of the RORγt-CBFβ interaction. Coincidently, amino acids 403–413 locate at the interface of RORγt predicted to interact with CBFβ (Figure 6C, top left). Subsequent alanine mutation of individual amino acids from 403 to 413 revealed that R407A, L410A, and E412A impaired RORγt function in stimulating the RORγt reporter (Supplemental Figure 7C), interacting with CBFβ (Supplemental Figure 7D) and supporting Th17 differentiation (Figure 7D). Consistently, these 3 point mutations also impaired CBFβ- and RORγt-stimulated RORγt reporter activity (Supplemental Figure 7E). Computer molecular dynamics simulations also predicted that both L410 and E412 maintain steady contact with RORγt, with high frequencies of 82% and 57%, respectively (Figure 7E), suggesting that mutation at these 2 amino acids would affect the RORγt-CBFβ interaction. In contrast to L410 and E412, R407 is not predicted to make direct contact with CBFβ. However, a strong ionic lock between R407 and E336 is crucial for maintaining the structure of the RORγt interface critical for binding to CBFβ (Figure 7E). Consequently, R407A mutation disrupts the ionic lock, altering the interface structure and thus disturbing the RORγt-CBFβ interaction. Among the 3 mutations, R407A impaired the CBFβ-RORγt interaction the most (Supplemental Figure 7D), correlating with the greatest reduction in stimulating RORγt reporter activity (Supplemental Figure 7, C and E) and supporting Th17 differentiation (Figure 7D). We then generated a double mutation (DM) with R407A and L410A, which further impaired RORγt function in binding to CBFβ (Figure 7F), stimulating RORγt reporter (Figure 7G and 7H) and supporting Th17 differentiation (Figure 7I) compared with the individual mutation, confirming the critical function of this identified motif for RORγt. Finally, we tested the function of RORγt mutants in supporting thymocyte development (Figure 7J). R407A, L410A, and DM impaired RORγt-dependent thymocyte development, with DM showing the greatest impairment. These results demonstrate that RORγt-CBFβ interaction is required for RORγt-mediated Th17 differentiation and thymocyte development.

### Mice expressing DM RORγt have defective Th17 differentiation and thymocyte development.

**Figure 8:**
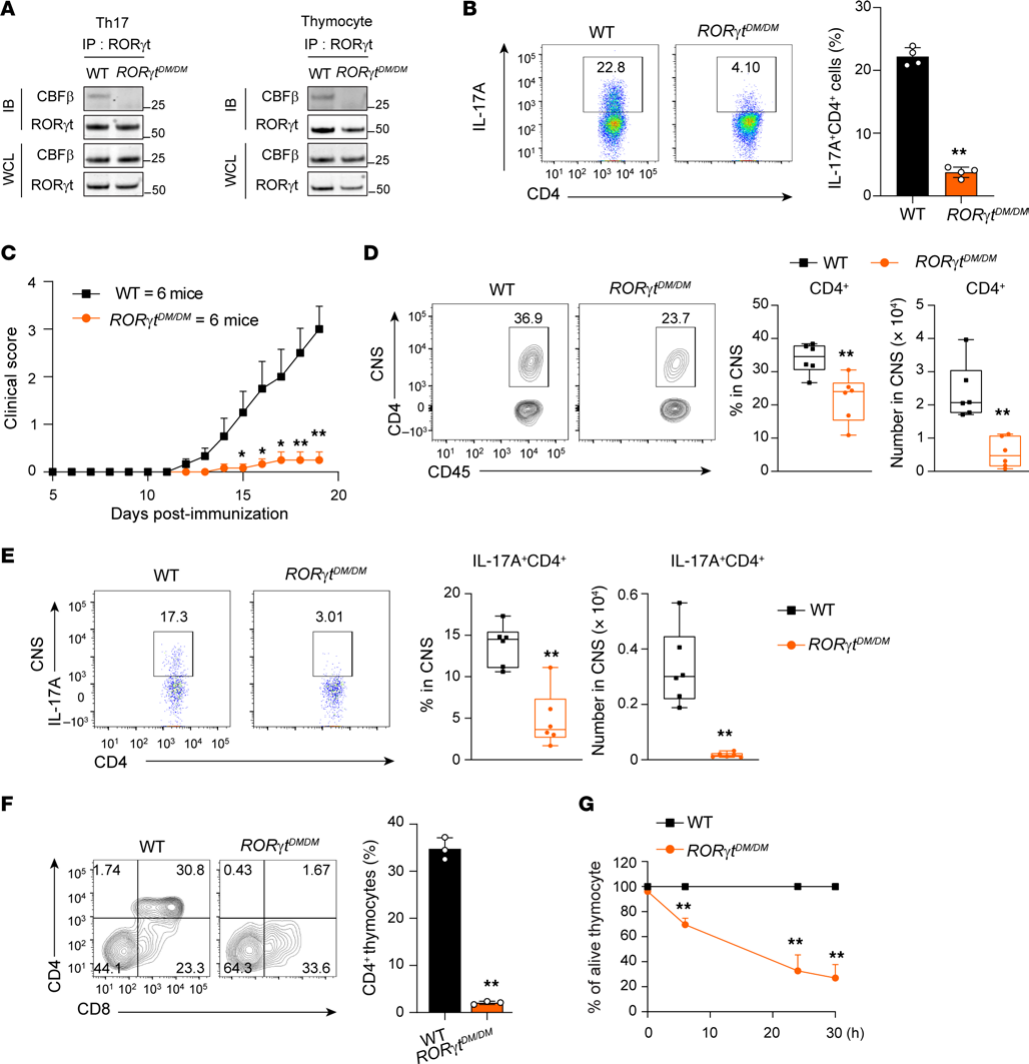
*RORγt^DM/DM^* mice have defective Th17 differentiation and thymocyte development. (**A**) IP analysis of the RORγt-CBFβ interaction in Th17 cells (left) or thymocytes (right) from indicated mice. Cell lysates from differentiated Th17 cells and thymocytes were subjected to IP with anti-RORγt antibody and immunoblotting with anti-CBFβ or (top) and anti-RORγt (bottom) antibody. Input CBFβ and RORγt were analyzed by Western blot (bottom 2 panels). Representative Western blots are shown from 3 independent experiments. (**B**) Flow cytometric analysis and percentage of IL-17A^+^ cells among CD4^+^ T cells from indicated mice polarized under Th17 conditions for 60 hours (*n* = 4 mice/group, from 1 experiment). (**C**) Clinical score of EAE among indicated mice (*n* = 6/group) different days after induction of disease by immunization with MOG_35–55_. (**D**) Flow cytometric analysis (left 2 panels), percentages (third panel), and numbers (right) of CD4^+^ T cells infiltrated into the CNS of EAE-induced mice shown in **C**. (**E**) Flow cytometric analysis (left 2 panels), percentages (third panel), and numbers (right) of CNS-infiltrated CD4^+^ T cells producing IL-17A in EAE-induced mice shown in **C**. (**F**) Flow cytometric analysis of CD4^+^ and CD8^+^ thymocytes ex vivo developed for 3 days from sorted WT or *RORγt^DM/DM^* CD4^–^CD8^–^ cells. Right: Summary of the percentage of CD4^+^ plus CD4^+^CD8^+^ T cells among live Thy1.2^+^ cells (*n* = 3 mice/group, from 1 experiment). (**G**) Percentage of live cells among thymocytes from indicated mice and cultured for different times in vitro (*n* = 4 mice/group, from 1 experiment). Data were analyzed by 2-tailed Student’s *t* test (**B**–**G**). *P < 0.05; **P < 0.01.

To determine the in vivo function of the RORγt-CBFβ interaction, we generated mice expressing DM RORγt (*RORγt^DM/DM^*). Sequencing analysis confirmed R407A and L410A mutations (Supplemental Figure 8A). An IP assay detected the RORγt-CBFβ interaction in WT but not *RORγt^DM/DM^* Th17 cells and thymocytes (Figure 8A), verifying that DM RORγt does not bind CBFβ. CD4^+^ T cells from *RORγt^DM/DM^* mice had greatly impaired Th17 differentiation (Figure 8B). Consistently, *RORγt^DM/DM^* mice were resistant to EAE induction (Figure 8C), which was associated with reduced numbers (Supplemental Figure 8B) and percentages (Figure 8D) of CNS-infiltrating lymphocytes, including CD4^+^ T cells that produced inflammatory IL-17A (Figure 8E) and GM-CSF (Supplemental Figure 8C). Additionally, Th17 cells in spleen of *RORγt^DM/DM^* mice were also reduced compared with the WT mice (Supplemental Figure 8D). These results demonstrate that disruption of the RORγt-CBFβ interaction in vivo prevents RORγt-dependent Th17 differentiation and Th17-mediated immune responses required for EAE development.

We next monitored RORγt-dependent thymocyte development in *RORγt^DM/DM^* mice. In an in vitro thymocyte differentiation system, CD4^–^CD8^–^ thymocytes from *RORγt^DM/DM^* mice did not differentiate into CD4^+^CD8^+^ cells (Figure 8F), similar to the CD4^–^CD8^–^ thymocytes from *RORγt^–/–^* mice (Figure 4A). Like the thymocytes in *RORγt^–/–^* mice, *RORγt^DM/DM^* mice had greatly reduced thymic cellularity (Supplemental Figure 8E) due to accelerated thymocyte apoptosis (Figure 8G), as reflected by the reduced percentage of CD4^+^CD8^+^ thymocytes (Supplemental Figure 8F). Furthermore, a markedly increased percentage of thymocytes with >2N DNA was observed in *RORγt^DM/DM^* mice (Supplemental Figure 8G), similar to *RORγt^–/–^* mice. Altogether, when the RORγt-CBFβ interaction is disrupted, RORγt-mediated functions in vivo in Th17 differentiation, Th17 immune responses, and thymocyte development are all greatly impaired. This explains why IMU-935, which preferentially disrupts the RORγt-CBFβ interaction in Th17 cells but not in thymocytes, selectively inhibits Th17 differentiation and Th17 immune responses without interfering with thymocyte development.

## Discussion

RORγt is an important drug target for treating various Th17-dependent autoimmune diseases (20, 34). However, germline deletion of RORγt leads to the development of thymic lymphoma due to disrupted thymocyte development (35), raising concerns that RORγt inhibitor–based treatments could induce lethal lymphoma. The adult human thymus has greatly decreased thymocyte development activity, known as involution, due to reduced influx of lymphoid progenitor cells (62, 63); thus, the induction of lymphoma by RORγt inhibitors may not be a relevant concern in adults. A recent study showed that the induced deletion of RORγt in adult mice leads to abnormal thymocyte development and lymphoma, similar to what is observed in mice with germline deletion of RORγt (32). Moreover, administration of an RORγt inhibitor not only prevented Th17 responses but also caused thymic changes and lymphoma in rats (33). These results discouraged pharmaceutical companies from further developing RORγt inhibitors for clinical applications (64). Our study identifies CBFβ as a previously unreported critical cofactor for RORγt in both Th17 cells and thymocytes. We further demonstrate that, in contrast to other RORγt inhibitors, IMU-935 preferentially disrupts the RORγt-CBFβ interaction in Th17 cells but not in thymocytes. As a result, IMU-935 effectively prevents Th17-mediated autoimmunity, such as in the EAE model, without affecting thymocyte development.

RORγt has 2 highly conserved domains (61, 65): a DNA binding domain with 2 zinc finger motifs responsible for DNA binding, and an LBD with a carboxyl terminal activation function 2 (AF2) motif that recruits steroid receptor coactivator (SRC) (28, 29). We have shown that both DNA binding activity and AF2 motif–mediated recruitment of SRC are required for RORγt-dependent Th17 differentiation and thymocyte development (14, 16, 17, 19, 28–30). Traditionally, inhibitors for nuclear receptors are identified by their ability to inhibit LBD-mediated transcription activation of a reporter gene by disrupting the interaction with SRC (66). Therefore, most RORγt inhibitors that disrupt overall RORγt-dependent transcription activity are expected to disable RORγt function in both Th17 cells and thymocytes. This is confirmed by our results showing that all the RORγt inhibitors we tested, except IMU-935, promote thymocyte apoptosis and Th17 differentiation. IMU-935 was identified by its ability to inhibit IL-17 production from activated human PBMCs. In addition, IMU-935 is highly specific for RORγt, even compared with RORα and RORβ, the 2 closest members of the ROR family of nuclear receptors. Our previous study suggested RORγt functions are separable in Th17 cells and thymocytes (14), indicating the possibility of developing an RORγt inhibitor that selectively inhibits RORγt function in Th17 cells but not in thymocytes. Indeed, IMU-935 is such a highly selective RORγt inhibitor, as demonstrated in the present study.

We identified a cofactor for RORγt, CBFβ, previously unreported to our knowledge, which is required for both RORγt-mediated Th17 differentiation and thymocyte development. CBFβ was identified 3 decades ago as a binding partner for RUNX transcription factors (67, 68). The only known function of CBFβ to date has been its interaction with RUNX to regulate gene expression, primarily in hematopoietic cells. However, differences in the expression patterns between CBFβ and RUNX across various tissues and development stages suggest CBFβ may have functions independent of RUNX. We identified CBFβ as a RORγt binding protein in Th17 cells, because the CBFβ-RORγt interaction is disrupted by IMU-935. Furthermore, mutations on RORγt that disrupt the CBFβ-RORγt interaction also impair RORγt function in Th17 differentiation and thymocyte development, indicating that the CBFβ-RORγt interaction is required for both processes. In contrast to its effects on Th17 cells, a much higher concentration of IMU-935 is required to disrupt the CBFβ-RORγt interaction in thymocytes, which explains the lack of the inhibitory effects of lower concentrations of IMU-935 on thymocyte development and survival. The vastly different concentrations needed to disrupt the RORγt-CBFβ interaction in Th17 cells and thymocytes are due to presence of different levels of RUNX1 that compete with RORγt for binding to CBFβ. Our results thus reveal that selective inhibition of RORγt by IMU-935 in Th17 differentiation, but not in thymocyte development, is due to preferential disruption of the CBFβ-RORγt interaction in Th17 cells but not in thymocytes. IMU-935, thus, is a superior inhibitor due to its selective inhibition of RORγt function in Th17 cells. Our study facilitates the development of new and selective small-molecule RORγt inhibitors for the treatment of Th17-mediated autoimmune diseases.

## Methods

### See the Supplemental Methods for additional information.

### Sex as a biological variable.

Our study examined male and female animals, and similar findings are reported for both sexes in all experiments.

### Mice.

C57BL/6J, *RORγt*^−/−^ (*Rorc^tm1Litt^;* stock 007571), *Rag1^–/–^* (*Rag1^tm1Mom^*; stock 002216), *Tg^TCR2D2^* (stock 006912), and CRISPR/Cas9-EGFP [Gt(ROSA)26Sor^em1.1(CAG-cas9*,-EGFP)Rsky^; stock 028555] mice were purchased from The Jackson Laboratory. *RORγt^DM/DM^* mice were designed and generated by GemPharmatech. The mice were housed under specific pathogen–free conditions, with food and water available ad libitum, and were acclimatized for 1 week before experimentation. For all experiments, littermates matched for age (6–12 weeks) were used.

### Statistics.

Data were analyzed with GraphPad Prism software and are reported as the mean ± SEM. One-way ANOVA or unpaired, 2-tailed Student’s *t* test was used to access statistical significance. A *P* value of less than 0.05 was considered significant.

### Study approval.

All mice studies were reviewed and approved by the Institutional Animal Care and Use Committee at the Beckman Research Institute of City of Hope (Duarte, California, USA). The human thymus sample was collected at the California Pacific Medical Center through the International Institute for the Advancement of Medicine (IIAM), with appropriate written consent and approval from the IIAM Authority Ethics Committee (Edison, New Jersey, USA).

### Data availability.

The RNA-Seq and ChIP-Seq data have been deposited in the NCBI Sequence Read Archive under accession number PRJNA1297911. Values for all data points in graphs are reported in the Supporting Data Values file.

## Author contributions

ZS and HW conceptualized the study. HW, XZ, NM, GW, GL, NV, ZH, WZ, YS, EP, CG, YG, IT, HHX, contributed to method development. HW, XZ, NM, YCY, and EP contributed to the investigation. HW, NM, TW, and CG contributed to data visualization. ZS, HK, and DV supervised the study. ZS and HW wrote the original draft of the manuscript. ZS, HW, EP, CG, and TW reviewed and edited the manuscript.

## Funding support

This work is the result of NIH funding, in whole or in part, and is subject to the NIH Public Access Policy. Through acceptance of this federal funding, the NIH has been given a right to make the work publicly available in PubMed Central.

- NIH grant R01-AI109644.
- City of Hope institutional pilot funding.
- The Jackie and Bruce Barrow Cancer Research Scholars’ Program.
- Arthur Riggs Diabetes & Metabolism Research Institute Innovative grant.
- Caltech–City of Hope Biomedical Research Initiative
- Research agreement with Immunic AG.
- NIH grant P30CA033572 (support for the animal, genomic, and flow cytometry cores used in this study).

## Supplementary Material

Supplemental data

Unedited blot and gel images

Supporting data values

## Figures and Tables

**Table 1:**
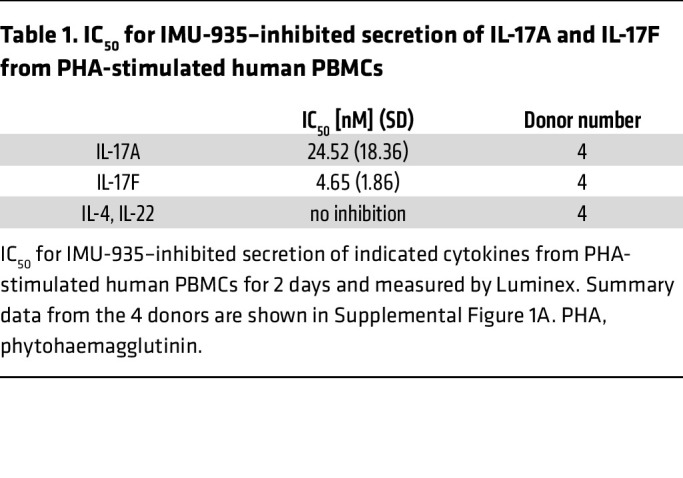
IC_50_ for IMU-935–inhibited secretion of IL-17A and IL-17F from PHA-stimulated human PBMCs

**Table 2:**
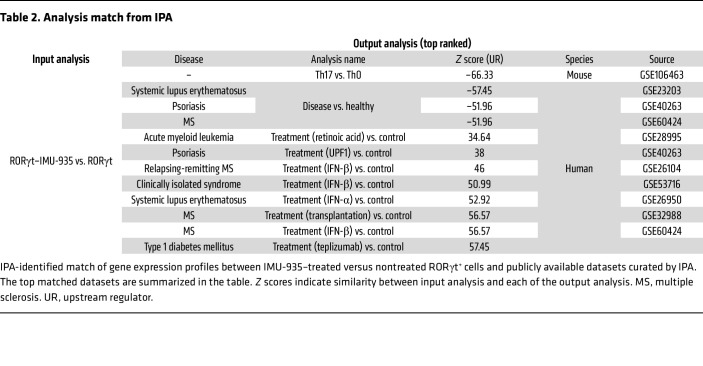
Analysis match from IPA

## Version 1 01/02/2026

Electronic publication
