# Supplementary material for: Selective disruption of RORγt-CBFβ interaction by IMU-935 prevents RORγt-dependent Th17 autoimmunity but not thymocyte development
Source: J Clin Invest. 2026 Jan 2;136(1):e185942. doi: 10.1172/JCI185942 (PMC12721887; doi:10.1172/JCI185942)
Supplement: Supplemental data [file jci-136-185942-s027.pdf]

Supplementary Materials

Selective disruption of RORγt-CBFβ interaction by IMU-935 prevents RORγt-dependent Th17 autoimmunity but not thymocyte development

Hongmin Wu[1,#], Xiancai Zhong[1,#], Ning Ma[2], Zhiheng He[1,&], Guanpeng Wang[1], Geming Lu[3], Yate-Ching Yuan[4], Wencan Zhang[1], Yun Shi[1], Nagarajan Vaidehi[2], Evelyn Peelen[5], Tanja Wulff[5], Christian Gege[5], Hella Kohlhof[5], Daniel Vitt[5], Yousang Gwack[6], Ichiro Taniuchi[7], Hai-Hui Xue[8], and Zuoming Sun[1,*]

[1] Department of Immunology & Theranostics, Arthur Riggs Diabetes & Metabolism Research Institute, Beckman Research Institute of the City of Hope, Duarte, CA, 91010.
[2] Department of Computational and Quantitative Medicine, Beckman Research Institute of the City of Hope, Duarte, CA, 91010.
[3] Department of Molecular & Cellular Endocrinology, Arthur Riggs Diabetes & Metabolism Research Institute, Beckman Research Institute of the City of Hope, Duarte, CA, 91010.
[4] Translational Bioinformatics, Department of Computational Quantitative Medicine, Beckman Research Institute of the City of Hope, Duarte, CA 91010
[5] Immunic AG, Lochhamer Schlag 21, 82166 Gräfelfing, Germany
[6] Department of Physiology, David Geffen School of Medicine, UCLA, Los Angeles, CA 90095
[7] Laboratory for Transcriptional Regulation, RIKEN Center for Integrative Medical Science, Yokohama, Kanagawa, 230-0045, Japan
[8] Center for Discovery and Innovation, Hackensack University Medical Center, Nutley, NJ 07110
[&] current address: Department of Molecular Microbiology and Immunology, Keck School of Medicine, University of Southern California, Los Angeles, CA 90033

[#] Those authors contributed equally to this work.
* Corresponding author

Address correspondence and reprint requests to: Zuoming Sun
Department of Immunology and Theranostics,
Arthur Riggs Diabetes & Metabolism Research Institute
Beckman Research Institute, City of Hope,
1500 East Duarte Road, Duarte, CA 91010
Phone: 626-256-4673
Fax: 626-256-6415
Email: zsun@coh.org

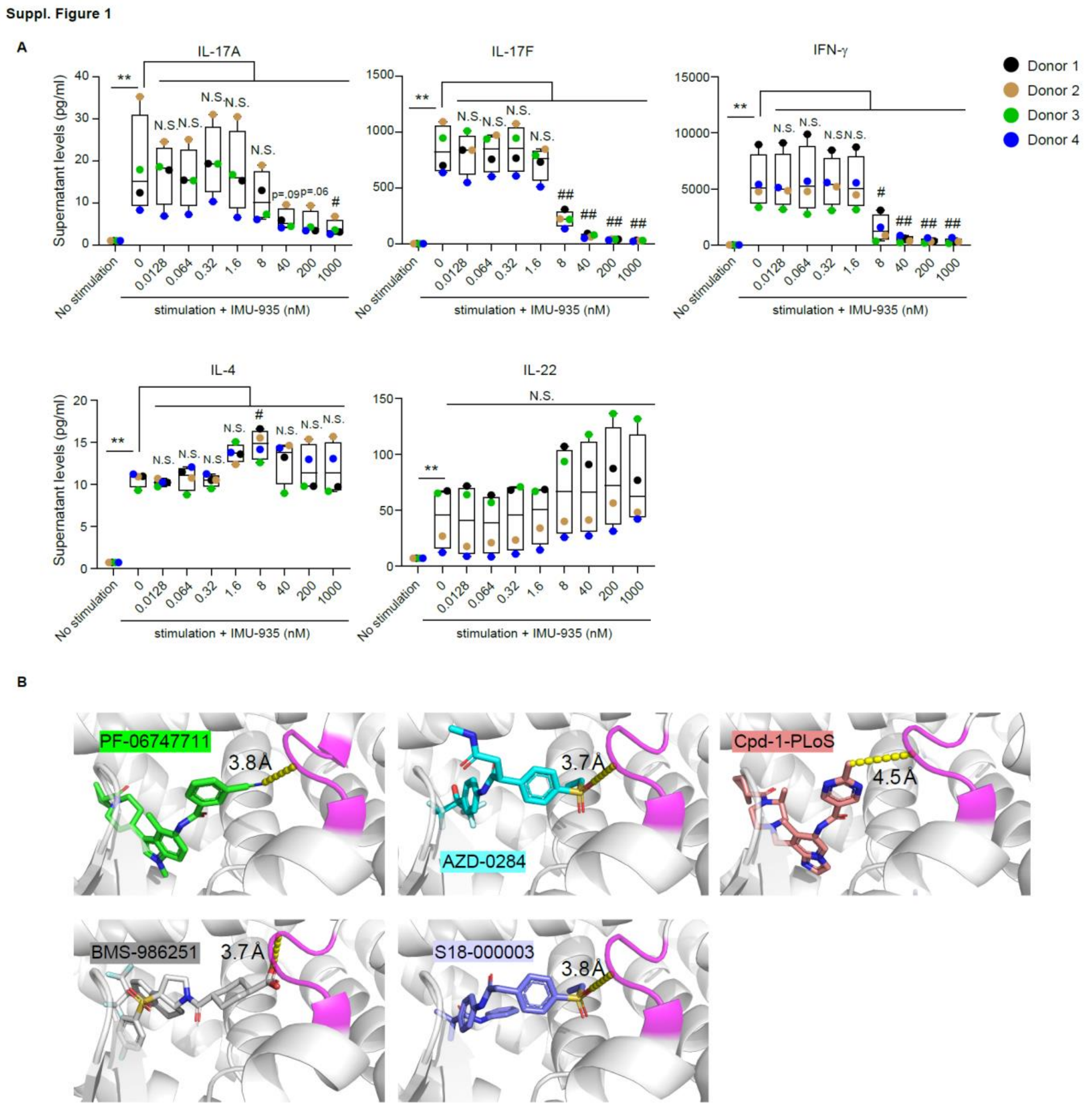

Supplementary Figure 1. IMU-935 potently inhibits IL-17 production from peripheral blood mononuclear cells (PBMC). (A) IL-17A, IL-17F, IFNγ, IL-4, and IL-22 produced from PHA-activated PBMC collected from individuals in the absence and presence of different concentrations of IMU-935, summarized in Table 1. (B) Visualization of indicated RORγt inhibitor in the binding-pocket of RORγt. The X-loop between H1 and H2 of RORγt-ligand-binding domain

is indicated by magenta. The number indicates the distance (yellow dashed line) between RORγt inhibitor and the X-loop.

Suppl. Figure 2

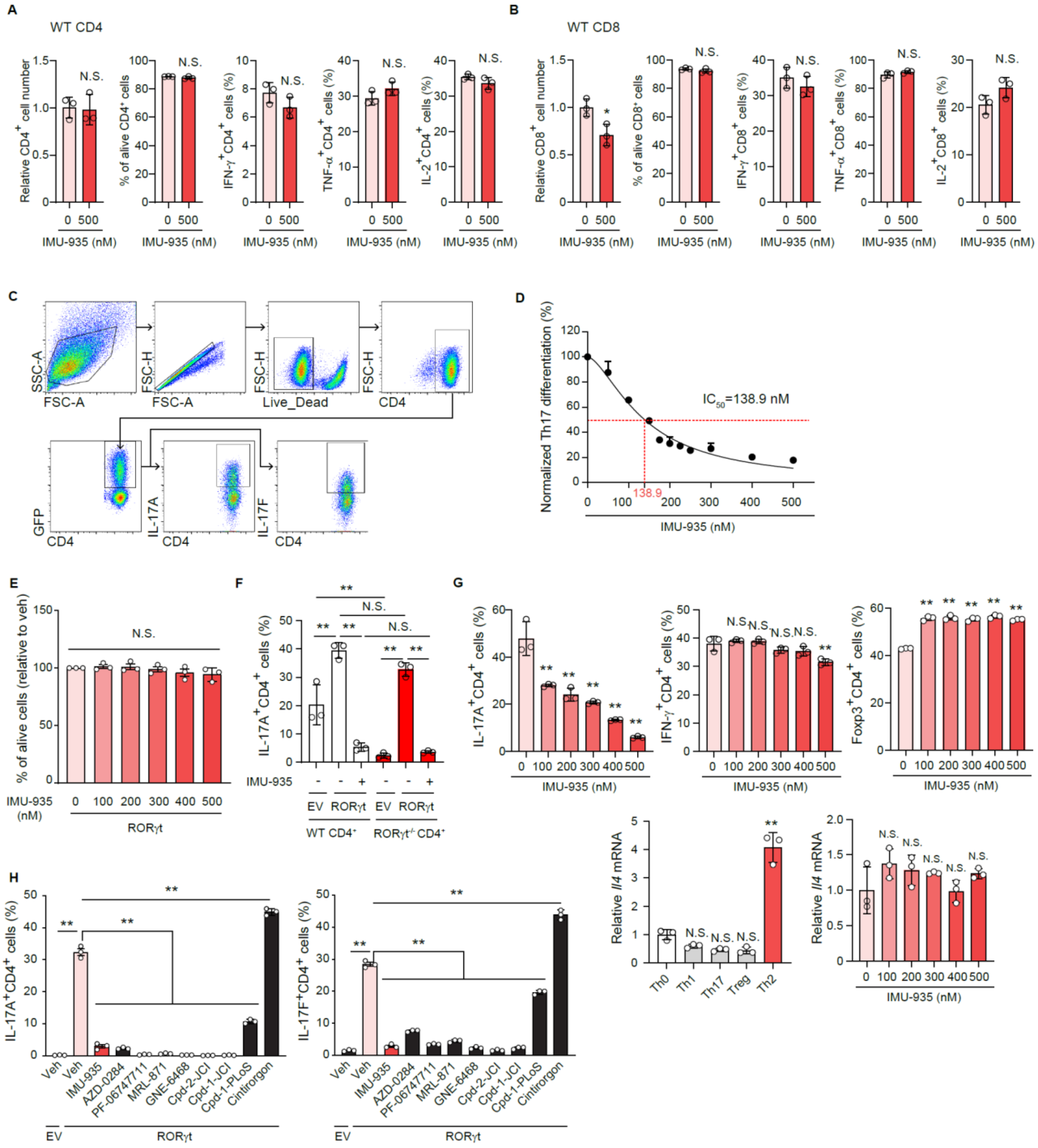

Suppl. Figure 2 continue

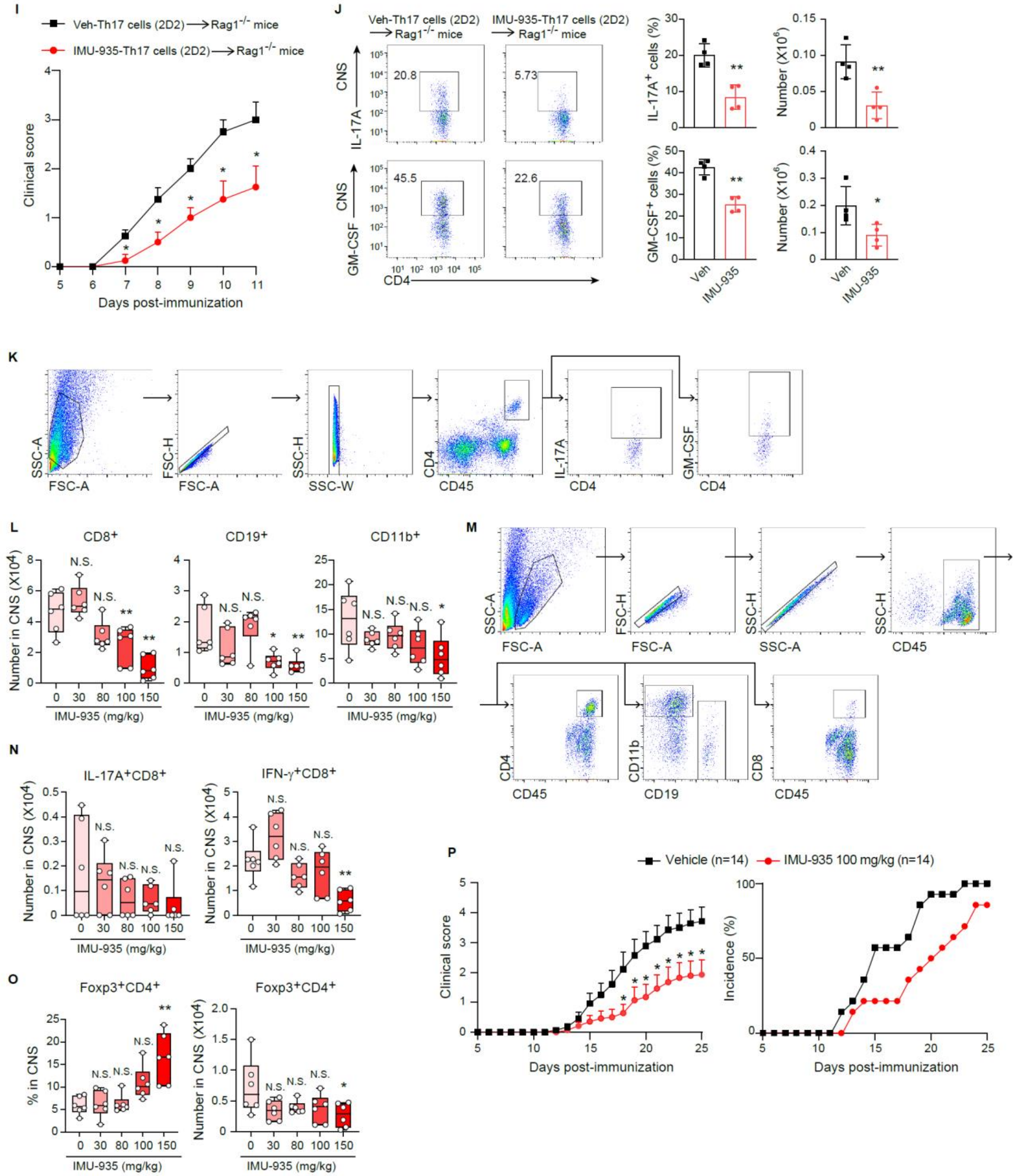

Supplementary Figure 2. IMU-935 prevents Th17-dependent EAE via inhibiting Th17 differentiation. (A-B) Number, percentage of live, and percentage of IFNγ$^+$, TNFα$^+$ and IL-2$^+$ CD4$^+$ (A) or CD8$^+$ (B) cells 48 hrs after CD3/CD28 stimulation in the absence and presence of

500 nM IMU-935 (added at 24 hrs after stimulation). (C) Gating strategy for *in vitro* Th17 differentiation for Figure 2A. (D) The measure of IC_{50} of IMU-935 in the inhibition of Th17 differentiation from WT $CD4^+$ T cells (n = 3/group). (E) Percentage of alive cell during Th17 differentiation in the presence of different concentrations of IMU-935 shown in Figure 2A. Apoptotic cells were determined by flow cytometric analysis of Annexin V and the membrane-impermeable amine-reactive Live_Dead dye. (F) Percentage of IL-$17A^+$ cells among WT $CD4^+$ or *RORγt*$^{-/-}$ $CD4^+$ T cells retrovirally expressing GFP alone (EV, empty virus) or with RORγt in the absence (-) or presence (+) of IMU-935 (500 nM, n = 3/group). (G) Percentage of IL-$17A^+CD4^+$, $IFNγ^+CD4^+$, and $Foxp3^+CD4^+$ cells among $CD4^+$ T cells 48 hrs after polarized under Th17, Th1 and Treg conditions respectively (top three panels). Bottom panels are the *Il4* mRNA levels in differentiated indicated $CD4^+$ T helpers (fourth panel) and in Th2 differentiated cells in the presence of different concentrations of IMU-935 (fifth panel). (H) Percentage of IL-$17A^+$ (left panel) and IL-$17F^+$ (right panel) cells among *RORγt*$^{-/-}$ $CD4^+$ T cells retrovirally expressing GFP alone (EV, empty virus) or with RORγt in the absence (-) or presence (+) of indicated RORγt inhibitors (n = 3/group, 500 nM). The percentage of IL-$17A^+CD4^+$ and IL-$17F^+CD4^+$ T cells were monitored by flow cytometry. (I) Clinical score of *Rag1*$^{-/-}$ recipients (n = 4/group) adoptively transferred with *Tg*^{Tcr2D2} $CD4^+$ T cells polarized under Th17 conditions $\pm$ 500 nM IMU-935 for 48 hrs. (J) Flow cytometric analysis, percentage and number of pathogenic IL-$17A^+$ and GM-CSF^+ among $CD4^+$ T cells that infiltrated the CNS of the recipients described in I. (K) Gating strategy for cytokine expression in adoptive and active EAE model described in Figure 2E. (L) Absolute number of indicated lymphocytes recovered from central nervous system (CNS) of the mice described in Figure 2C. (M) Gating strategy for lymphocytes obtained from of the active EAE model described in L. (N) Number of $CD8^+$ T cells producing indicated cytokines in CNS of the mice described in Figure 2C. (O) Percentage and number of $Foxp3^+$ Tregs in CNS of the mice described in Figure 2C. (P) Clinical score (left) and incidence (right) of EAE among mice (n = 14/group) immunized with MOG_{35-55} for EAE induction and treated with vehicle control or 100 mg/kg IMU-935 for the

duration of the experiment. Statistical difference was determined by two-tailed *t*-test (A-B, I-J, and P) or one-way ANOVA (E-H, L, and N-O). *P<0.05; **P<0.01. Abbreviation: N.S., not significant; Veh, vehicle.

Suppl. Figure 3

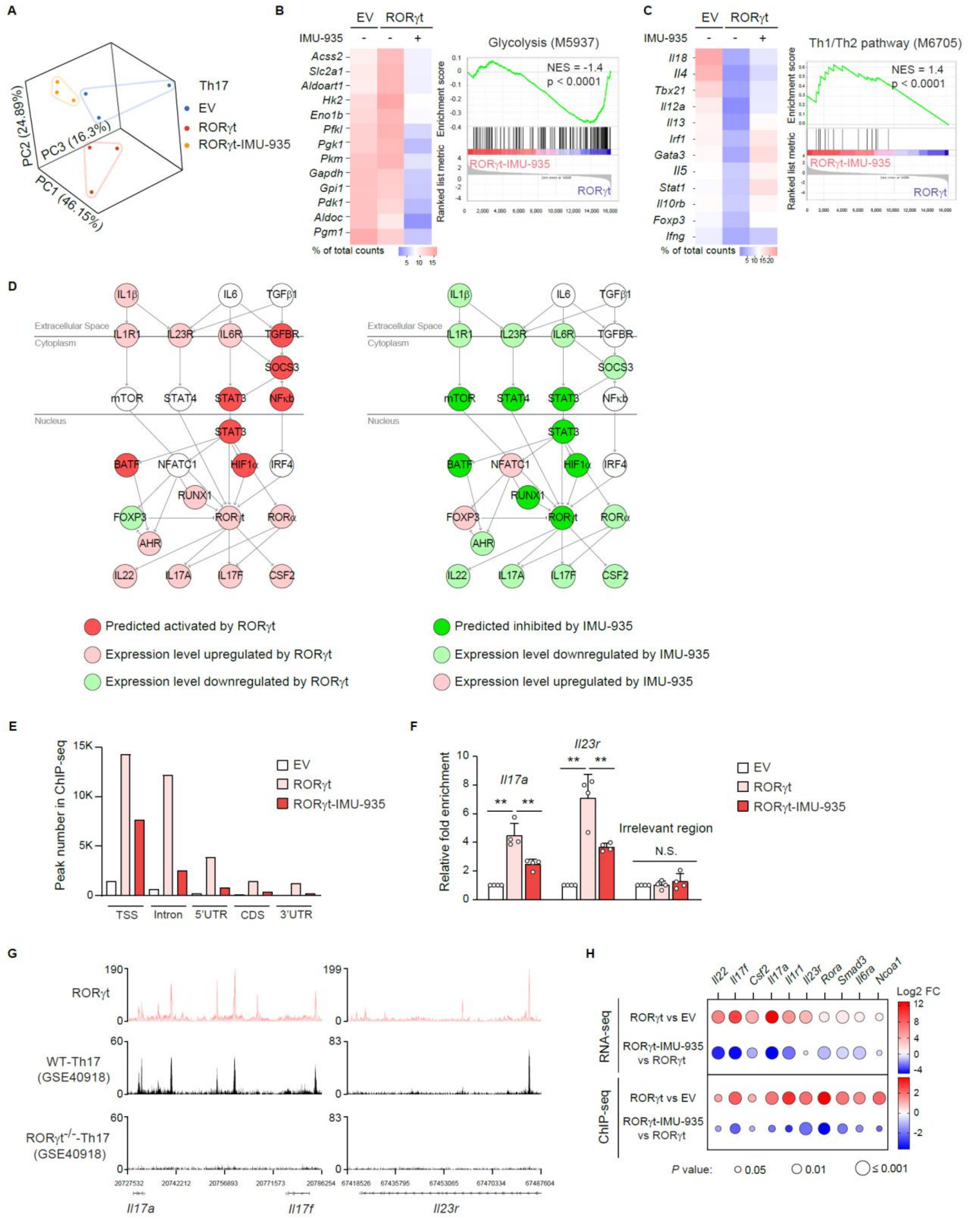

Supplementary Figure 3. IMU-935 inhibits RORγt target genes critical for Th17

differentiation. (A) Principal component analysis of RNA-sequencing data indicates similar gene expression patterns within each of the three indicated groups. Three biological replicates from each group are shown. (B) Heatmap showing expression of genes critical for glycolytic enzymes in *RORγt*$^{-/-}$ (EV) or IMU-935-nontreated (-) or treated (+) RORγt^{+} CD4^{+} T cells polarized under T_H17 conditions for three days. Right panel is the gene set enrichment plot showing enrichment of glycolysis genes between IMU-935-treated vs nontreated RORγt^{+} cells. Glycolysis gene set was derived from MSig database. (C) Heatmap showing Th1/Th2/Treg signature gene expression in *RORγt*$^{-/-}$ (EV) or $\pm$ IMU-935-treated RORγt^{+} CD4^{+} cells. Right is the gene set enrichment plot showing enrichment of Th1/Th2 genes. Gene expression was normalized by total counts of each gene. Specific Th gene sets were derived from MSig database. (D) Activity of a network of genes critical for Th17 differentiation between RORγt^{+} vs RORγt$^{-/-}$ cells (left panel) and IMU-935-treated vs nontreated RORγt^{+} cells (right panel). Red or green indicate regulator's up or down-regulated activity. Pink or light green indicate genes' up or down-regulated expression by RORγt (left panel) or by IMU-935 (right panel), respectively. Genes critical for Th17 activation pathway were customized based on IPA curated pathways. (E) The number of RORγt-binding peaks identified by ChIP-seq assays in indicated regions in *RORγt*$^{-/-}$ CD4^{+} T cells retrovirally expressing GFP alone (EV, blank columns) or with RORγt in the absence (pink columns) or presence (red columns) of IMU-935, polarized under T_H17 conditions for 72 hrs. Abbreviation: TSS, regions around transcription start site; CDS, coding sequence exon regions; UTR, untranslated regions. The peaks were identified by MACS2. (F) ChIP-qPCR analysis of RORγt-binding to the *Il17a* and *Il23r* loci. A non-RORγt-binding (irrelevant) region was included as a negative control. Data represents mean ± SEM from four separate experiments by one-way ANOVA analysis. **$P<0.01$; N.S., not statistically significant. (G) RORγt-DNA-binding signals at *Il17a* and *Il17f* loci (left panel) and *Il23r* gene locus (right panel, identified by our current study (top row) and published references in WT Th17 cells (middle row) and *RORγt*$^{-/-}$ cells (bottom row). Referenced data were extracted from Ciofani M *et al* (2012). (H) Correlation of expression of the Th17 signature genes with RORγt-

DNA binding signals to the gene loci. Upper panel shows the fold change of indicated Th17 genes (RNA-seq) and bottom panel shows the fold change of RORγt-DNA binding signals (ChIP-seq) at the indicated gene loci in $ROR\gamma t^{+}$ vs *RORγt*$^{-/-}$ (EV) cells (top row of the panel) or between ± IMU-935-treated $ROR\gamma t^{+}$ $CD4^{+}$ cells (bottom row of the panel).

Suppl. Figure 4

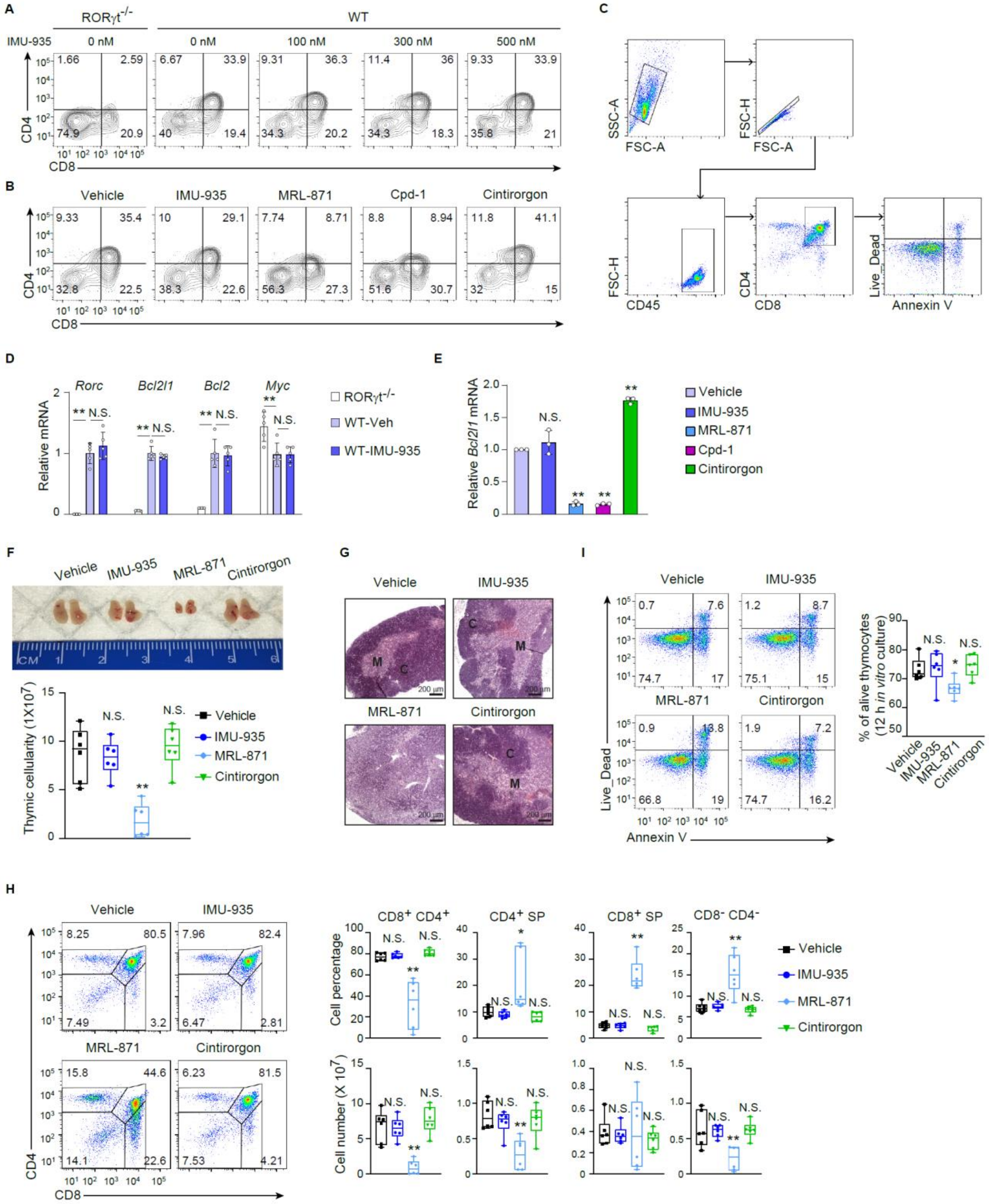

Suppl. Figure 4 continue

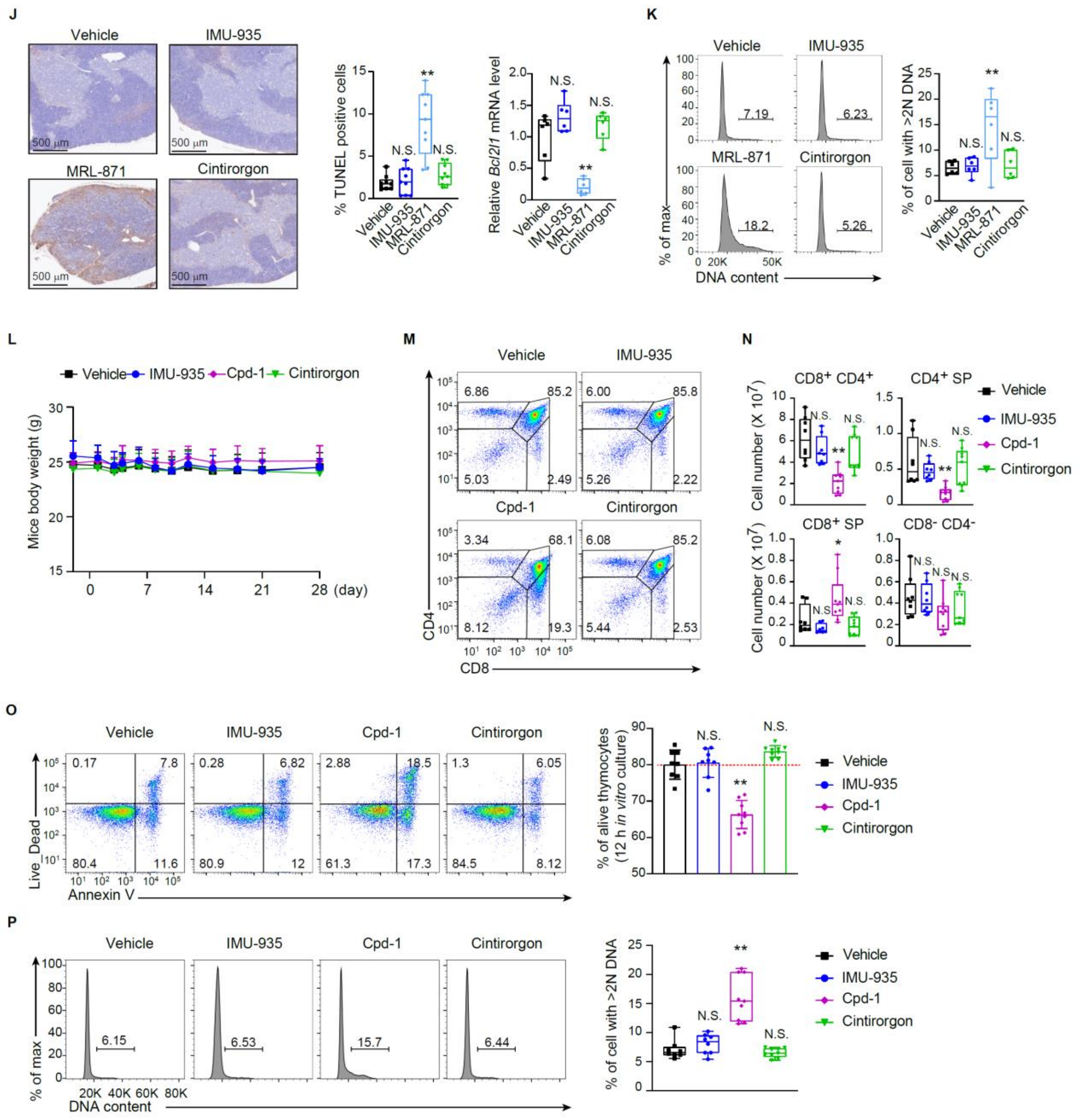

Supplementary Figure 4. IMU-935 does not affect thymocyte development and survival.

(A-B) Representative flow cytometric analysis of $CD4^+$ and $CD8^+$ thymocytes *ex vivo* developed for three days from sorted *RORγt*$^{-/-}$$CD4^-CD8^-$ or WT $CD4^-CD8^-$ in the presence of different concentrations of IMU-935 (A) or RORγt modulators (B) (1 μM, n = 4/group). (C) Gating strategy for mouse thymocyte viability test shown in Figure 4C. (D-E) qPCR analysis of indicated mRNA levels in thymocytes treated with vehicle control or 500 nM IMU-935 (D) or RORγt modulators (E)

for 24 hrs, and *RORγt*$^{-/-}$ thymocytes were used as control. (F) Pictures (top panel) and cellularity (bottom panel) of the thymus from mice (n = 6/group) treated with vehicle, indicated RORγt inhibitors or cintirorgon (p.o., b.i.d., 100 mg/kg) for three days. (G) H&E staining section (scale bar = 200 μM) of the thymus shown in F. M indicates medullar, and C indicates cortex. (H) Representative flow cytometric analysis of CD4 and CD8 of the thymocytes (left panels) and the percentage (top right panels) and absolute cell number (bottom right panels) of indicated subsets of thymocytes from mice treated shown in F. (I) Representative flow cytometry analysis (left panels) and percentage (right panel) of live thymocytes from mice shown in F using Annexin V and Live_Dead dye. Thymocytes were cultured for 12 hrs *in vitro*. (J) TUNEL staining apoptotic cells (left panels, scale bar = 500 μM) and percentage of apoptotic cells in thymus (middle) from mice shown in F. Right panel is the qRT-PCR analysis of anti-apoptotic *Bcl2l1* mRNA in thymus. (K) Representative flow cytometric analysis of the DNA content (PI staining) in thymocytes from thymus shown in F. Numbers above bracketed lines indicate percentage of cells with >2N DNA. Right panel is the summary of the percentage of cells with >2N DNA shown on left. (L) Body weight of the mice treated with indicated RORγt inhibitors and cintirorgon for indicated days as described in Figure 4E. (M) Flow cytometric analysis of CD4 and CD8 of the thymocytes from mice treated as described in Figure 4E. (N) Absolute cell number of indicated subsets of thymocytes from mice shown in Figure 4E. (O) Flow cytometry analysis (left panel) and quantification (right panel) of apoptotic thymocytes in mice described in Figure 4E. Thymocytes were cultured for 12 hrs. (P) Flow cytometric analysis of the DNA content (PI staining) of thymocytes obtained from the mice described in Figure 4E. Numbers above bracketed lines (left panels) indicate percentage of cells with >2N DNA. Right panel is the summary of the percentage of cells with >2N DNA shown on left. Statistical difference was determined by one-way ANOVA. *P<0.05; **P<0.01; N.S., not significant.

Suppl. Figure 5

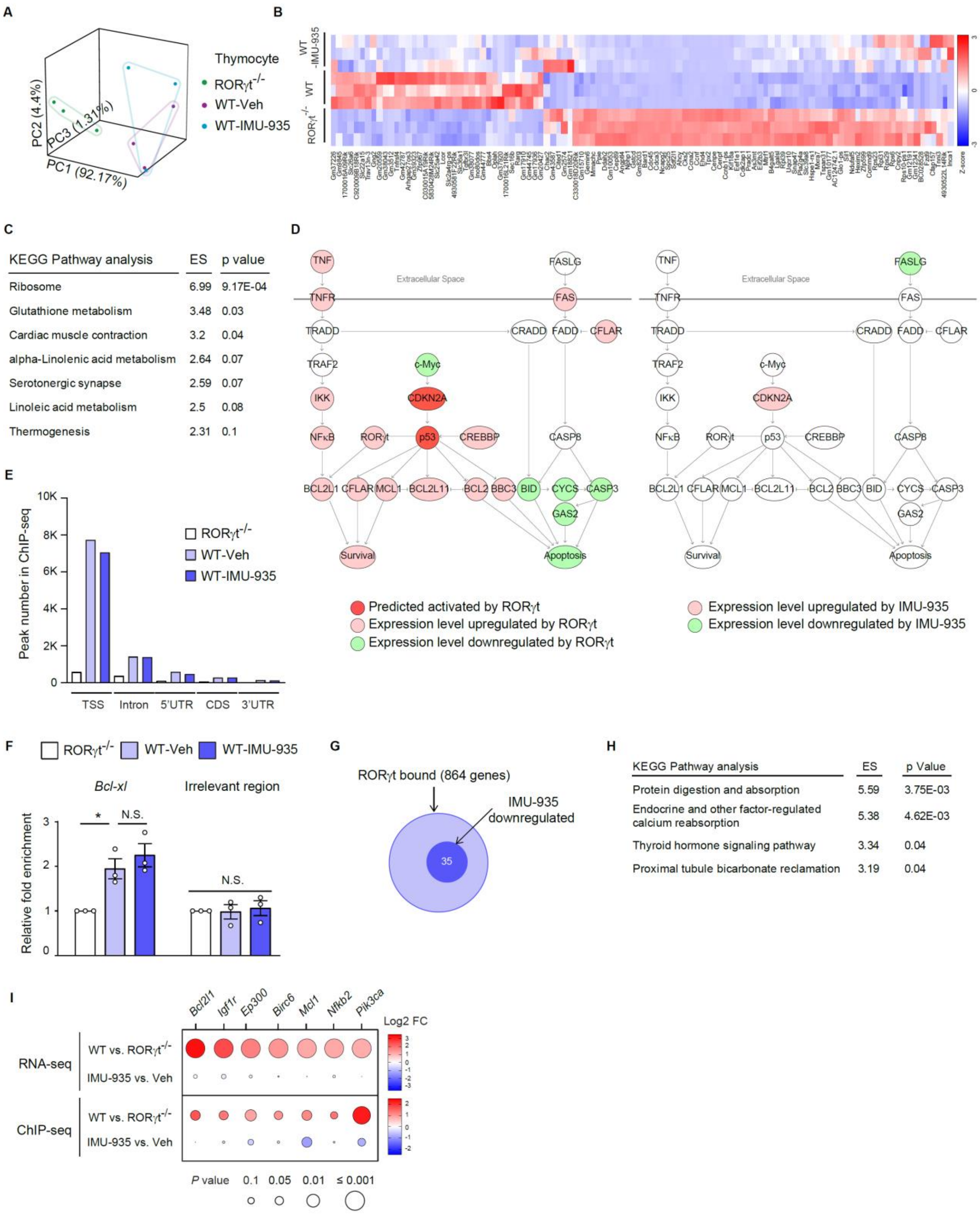

Supplementary Figure 5. IMU-935 does not affect RORγt target genes critical for thymocyte development and survival. (A) Principal component analysis of RNA-sequencing data indicates

similar gene expression patterns within each of the three indicated groups. Three biological replicates from each group are shown. (B) Heatmap of differentially expressed genes by RORγt and IMU-935 treatment in thymocytes (37 genes upregulated by RORγt and downregulated by IMU-935; 71 genes downregulated by RORγt and upregulated by IMU-935), data corresponds to Figure 5A. (C) KEGG pathway analysis of differentially expressed genes by RORγt and IMU-935 treatment in thymocytes (37 genes upregulated by RORγt and downregulated by IMU-935; 71 genes downregulated by RORγt and upregulated by IMU-935), data corresponds to Figure 5A. These genes are not involved in survival of the thymocytes. (D) Activity of a network of genes critical for thymocyte survival between WT vs *RORγt*$^{-/-}$ cells (left) and IMU-935-treated vs nontreated RORγt^{+} cells (right). Red or green indicate regulator's up or down-regulated activity. Pink or light green indicate genes' up or down-regulated expression by RORγt (left) or by IMU-935 (right), respectively. Genes critical for thymocyte survival were customized based on IPA curated pathways. (E) The number of RORγt-binding peaks identified by ChIP-seq assays in indicated regions in *RORγt*$^{-/-}$ thymocytes (blank columns) or WT thymocytes nontreated (light blue columns) or treated (dark blue columns) with IMU-935 inhibitor. Abbreviation: TSS, regions around transcription start site; CDS, coding sequence exon regions; UTR, untranslated regions. The peaks were identified by MACS2. (F) ChIP-qPCR analysis of RORγt-binding at the *Bcl2l1* locus. A non-RORγt-binding (irrelevant) region was included as a negative control. Data represents mean ± SEM from four separate experiments (one-way ANOVA analysis). *P<0.5; N.S., not statistically significant. (G) The number of genes bound by RORγt (light blue, 864 genes) and the number of genes with decreased RORγt-binding signal by IMU-935 (dark blue, 35 genes) among RORγt bound genes. (H) KEGG pathway analysis for the 35 genes whose RORγt-binding signals were downregulated by IMU-935 in thymocyte ChIP-seq assays. (I) Correlation of expression of the apoptotic genes with RORγt DNA-binding signals to the gene loci. Upper panel shows the fold change in the expression of indicated apoptotic genes (RNA-seq) and lower panel shows the fold change of RORγt DNA-binding signals (ChIP-seq) at the indicated gene loci

between WT and *RORγt*$^{-/-}$ (top row of the panel) or IMU-935-treated and nontreated WT (bottom row of the panel) thymocytes.

Suppl. Figure 6

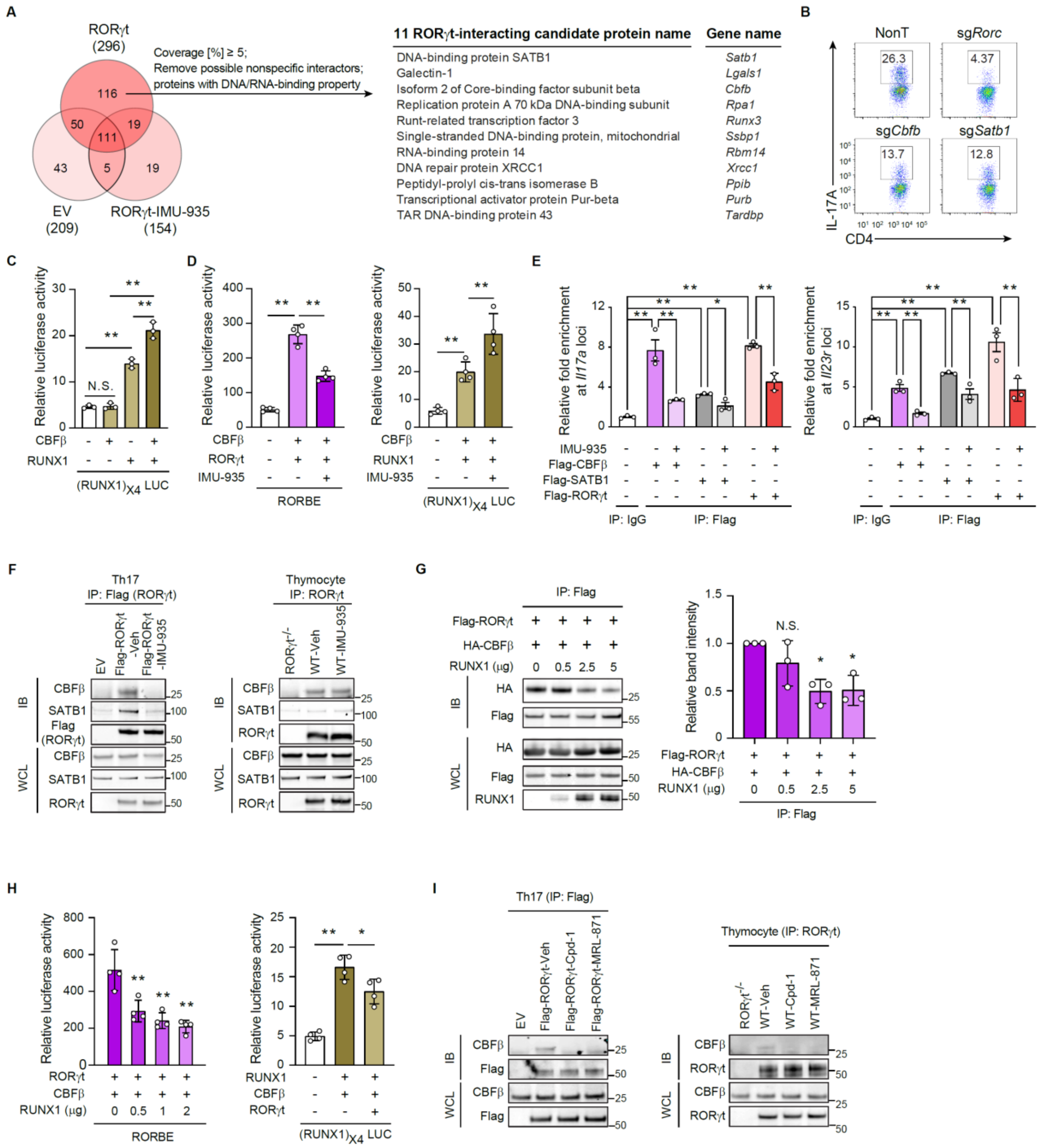

Supplementary Figure 6. IMU-935 inhibits the RORγt interaction with co-factor CBFβ in Th17 cells but not in thymocytes. (A) Identification IMU-935-regulated RORγt interacting proteins using mass spectrometry. *RORγt*$^{-/-}$ CD4^{+} T cells were transduced with retrovirus expressing empty vector (EV) or wild-type RORγt (RORγt, Flag-tagged), followed by polarizing to

Th17 for 60 hrs in the presence or absence of IMU-935 (500 nM). Lysates of differentiated Th17 cells were immunoprecipitated with anti-Flag antibody, which then subjected to mass spectrometry (MS) analysis. MS identified 296 RORγt-interacting proteins. After excluding nonspecific-binding proteins that were also detected in *RORγt*$^{-/-}$ cells and in the presence of IMU-935 (interactions with RORγt are not affected by IMU-935), the remaining 116 proteins were only detected in the absence but not presence of IMU-935. Among the 116 proteins whose interactions with RORγt were regulated by IMU-935, 11 were considered as transcription regulators (listed on right panel), which were then selected for CRISPR/Cas9-mediated deletion analysis to determine their function in Th17 differentiation shown in Figure 6A. (B) Representative flow cytometry plots corresponding to Figure 6A. (C) Relative luciferase activity from HEK293T cells transfected with a RUNX1 luciferase reporter containing four RUNX1-binding sites ((RUNX1)X4 LUC) and indicated expression plasmids for 24 hours. (D) Relative luciferase activity from HEK293T cells transfected with RORγt reporter (RORBE, left panel) or RUNX1 reporter (RUNX1)X4 LUC (right panel) and indicated expression plasmids in the presence and absence of IMU-935 for 24 hours. (E) ChIP-qPCR analysis of RORγt-binding signals at *Il17a* (top panel) or *Il23r* (bottom panel) locus in WT CD4^{+} cells retrovirally expressing GFP alone (first column) or CBFβ (2nd and 3rd columns) or SATB1 (4th and 5th columns) or RORγt (6th and 7th columns) ± IMU-935 and differentiated under Th17 polarization conditions. (F) Immunoprecipitation (IP) analysis of RORγt-CBFβ (top panel) or RORγt-SATB1 (second panel) interaction in Th17 cells derived from Flag-RORγt expressing *RORγt*$^{-/-}$ CD4^{+} T cells (left panels) or thymocytes (right panels). Cell lysates from differentiated Th17 cells and thymocytes were subjected to IP with anti-Flag (for Th17 cells) or anti-RORγt (for thymocytes) antibody and immunoblot (IB) with anti-CBFβ or anti-SATB1 antibody. Input CBFβ, SATB1 or RORγt was analyzed by western blot analysis (WB) of whole-cell lysates (WCL) (bottom three panels). (G) Immunoprecipitation analysis of RORγt-CBFβ interaction. HEK293T cells transfected with consistent amount of expression plasmids for Flag-RORγt and HA-CBFβ with increasing amount of expression plasmid for RUNX1. Cell lysates were immunoprecipitated with

anti-Flag (RORγt) antibody and immunoblot with anti-HA (CBFβ, top panel) or anti-Flag (RORγt, second panel) antibody. Input proteins were immunoblot with anti-HA, anti-Flag, or anti- RUNX1 antibody (bottom three panels). Right panel is the quantification for HA (CBFβ) band from 3 repeated experiments. (H) Relative luciferase activity from HEK293T cells transfected with RORγt reporter (RORBE) (left panel) or RUNX1 reporter (RUNX1)X4 LUC (right panel) and indicated expression plasmids including increasing amount expression plasmids for RUNX1 (left panel) for 24 hours. (I) Immunoprecipitation (IP) analysis of RORγt-CBFβ interaction in Th17 cells derived from Flag-RORγt expressing $RORγt^{-/-}$ CD4 T cells (left panels) or thymocytes (right panels) in the absence (Veh) or presence of indicated RORγt inhibitors. Whole-cell lysates from differentiated Th17 cells and thymocytes were subjected to immunoprecipitation with anti-Flag (for Th17 cells) or anti-RORγt (for thymocytes) antibody and immunoblot with anti-CBFβ (top panel) or anti-Flag (RORγt, second panel). Input CBFβ and RORγt were analyzed by western blot analysis (WCL) (bottom two panels). Statistical difference was determined by one-way ANOVA. *P<0.05; **P<0.01. Abbreviation: N.S., not significant.

Suppl. Figure 7

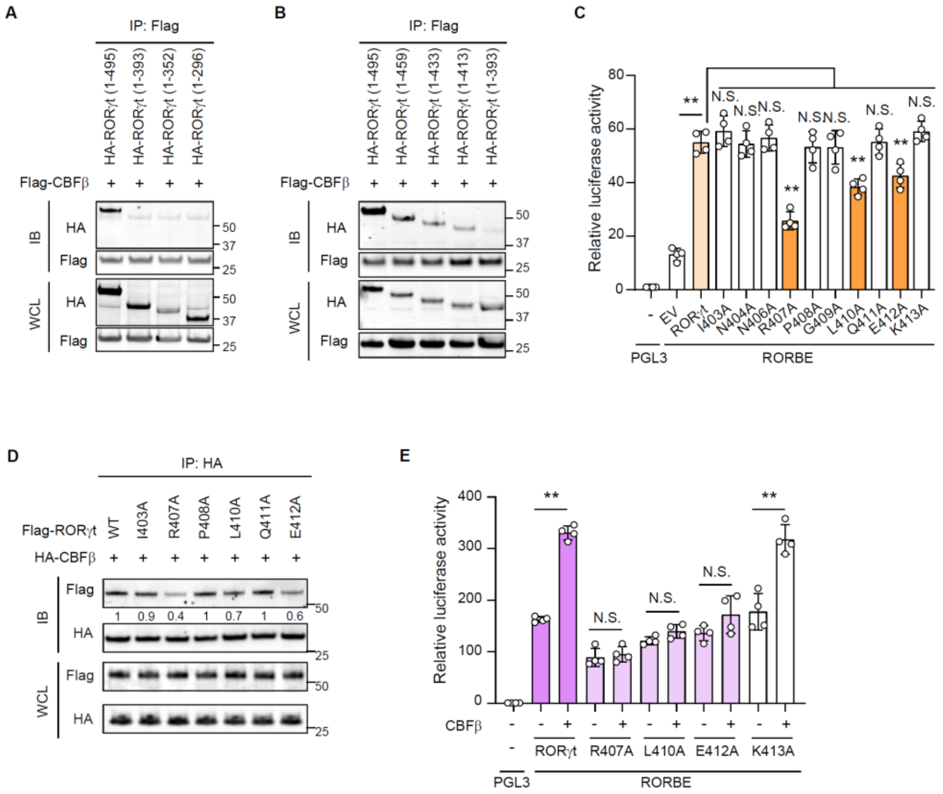

Supplementary Figure 7. The interaction between RORγt and CBFβ is essential for Th17 differentiation and thymocyte development. (A-B) Immunoprecipitation analysis of RORγt-CBFβ interaction in HEK293T cells transfected with plasmids expressing Flag-CBFβ and HA-WT or a series of RORγt deletion mutants between amino acid 296-495. The cell lysates were immunoprecipitated with Flag (CBFβ) antibody and immunoblot with anti-HA (RORγt) (top panel) or ani-Flag (CBFβ) (second panel). RORγt (1-495), full length mouse RORγt; RORγt (1-393), RORγt with amino acids 394-495 deletion; RORγt (1-352), RORγt with amino acids 353-495 deletion; RORγt (1-296), RORγt with 297-495 deletion; RORγt (1-459), RORγt with 460-495 deletion; RORγt (1-433), RORγt with 444-495 deletion; RORγt (1-413), RORγt with 414-495 deletion; RORγt (1-292), RORγt with 394-495 deletion. Input proteins were immunoblot with anti-HA or anti-Flag antibody (bottom two panels). (C) Relative luciferase activity from HEK293T cells transfected with promoterless pGL3 control luciferase reporter (pGL3) or a RORγt reporter (RORBE) together with plasmids expressing WT or RORγt with indicated point mutation. (D) IP

analysis of RORγt-CBFβ interaction in HEK293T cells transfected with plasmids expressing HA-CBFβ and WT Flag-RORγt or RORγt with indicated point mutation. (E) Relative luciferase activity from HEK293T cells transfected with control pGL3 or RORγt reporter (RORBE) together with plasmids expressing WT or RORγt with indicated point mutation ± CBFβ expression plasmid. Statistical difference was determined by one-way ANOVA. **P<0.01. Abbreviation: N.S., not significant.

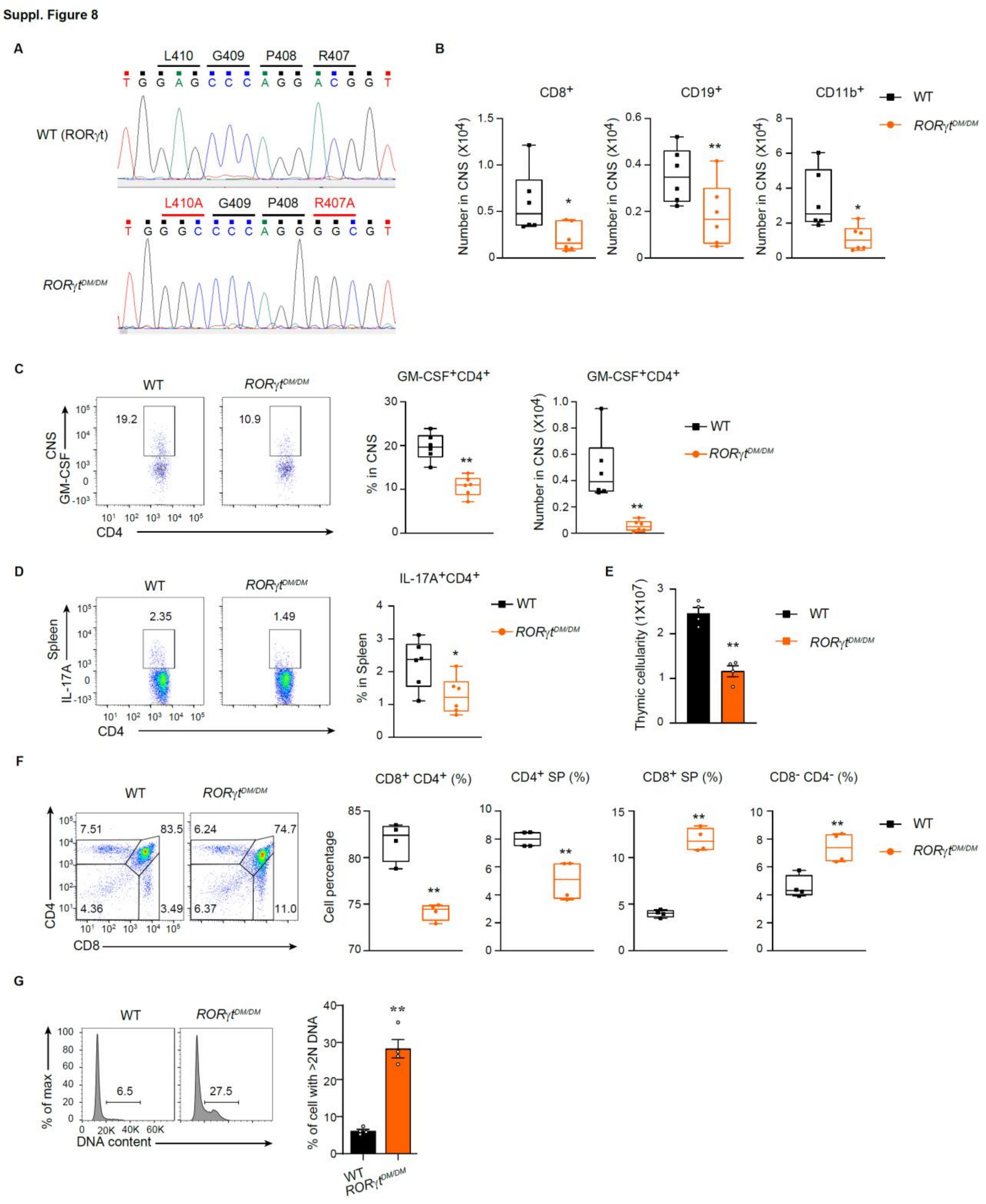

Supplementary Figure 8. (A) Sequencing analysis of WT (R407/L410) and mutated (R407A/L410R) allele. (B) Number of CD8$^+$, CD19$^+$ and CD11b$^+$ lymphocytes infiltrated into the CNS of the indicated EAE-induced mice shown in Fig. 8C. (C) Flow cytometric analysis (left),

percentage (middle) and number (right) of CNS-infiltrated $CD4^+$ T cells that producing GM-CSF in EAE-induced mice shown in Figure 8C. (D) Flow cytometric analysis and percentage of IL-17A$^+$ cells among $CD4^+$ T cells in spleen of EAE-induced mice shown in Figure 8C. (E) Total thymocyte number of indicated mice (n = 4/group). (F) Representative flow cytometric analysis of CD4 and CD8 of the thymocytes from indicated mice. Right four panels are the percentage of $CD4^+CD8^+$ double positive, $CD4^+$ single positive, $CD8^+$ single positive and $CD4^-CD8^-$ double negative thymocytes from indicated mice based on the analysis shown on left (n = 4/group). (G) Flow cytometric analysis of the DNA content (PI staining) of thymocytes obtained from indicated mice. Numbers above bracketed lines (left two panels) indicate percentage of cells with >2N DNA. Right panel is the summary of the percentage of cells with >2N DNA shown on left (n = 4/group). Statistical difference was determined by two-tailed *t*-test. *P<0.05; **P<0.01.

Supporting TABLE S1. Information on Antibodies, Cytokines, or Other Reagents Used

		Supplier	Catalog No.	Clone No.
FACS antibody	PE/Cyanine7 anti-mouse IL-17A Antibody	Invitrogen	25-7177-82	eBio17B7
	PE anti-mouse IL-17F Antibody	BioLegend	517007	9D3.1C8
	BV605 nti-mouse CD4 Antibody	BioLegend	100548	RM4-5
	PE anti-mouse GM-CSF Antibody	BioLegend	505406	MP1-22E9
	FITC anti-mouse CD45 Antibody	BioLegend	103108	30-F11
	APC/Cyanine7 anti-mouse CD45 Antibody	BioLegend	103116	30-F11
	APC anti-mouse CD45 Antibody	BioLegend	103112	30-F11
	FITC anti-mouse CD8a Antibody	BioLegend	100804	5H10-1
	APC anti-mouse CD19 Antibody	BioLegend	152410	1D3/CD19
	PE anti-mouse CD11b Antibody	ThermoFisher	12-0112-82	M1/70
	PE Rat Anti-Mouse CD90.2 (Thy1.2)	BD Biosciences	553014	30-H12
	APC Mouse Anti-Human CD45	BD Biosciences	555485	HI30
	FITC anti-human CD4 Antibody	BioLegend	300506	RPA-T4
	PE/Cyanine7 anti-human CD8a Antibody	BioLegend	300914	HIT8a
	LIVE/DEAD™ Fixable Near-IR Dead Cell Stain Kit	ThermoFisher	L34976	N/A
	PE Annexin V	BD Biosciences	556421	N/A
IP/WB antibody	Monoclonal Anti-FLAG Antibody	Sigma-Aldrich	F1804	M2
	HA-Tag Rabbit mAb	Cell Signaling	3724	C29F4
	Anti-CBFβ Antibody	Abcam	ab125191	N/A
	Recombinant Anti-SATB1 Antibody	Abcam	ab109122	EPR3951
	ROR gamma (t) Monoclonal Antibody	Invitrogen	14-6988-82	AFKJS-9

	Anti-RUNX1 Antibody	Santa Cruz	sc-365644	A-2
	IRDye 680RD Detection Reagent	LI-COR	926-69100	N/A
Antibodies and cytokines for T cell culture	Anti-mouse CD3ε Antibody	BioLegend	100359	145-2C11
	Anti-mouse CD28 Antibody	BioLegend	102121	37.51
	Anti-mouse IL-4 Antibody	BioLegend	504135	11B11
	Anti-mouse IFN-γ Antibody	BioLegend	505710	R4-6A2
	Human TGF-b1	Miltenyi	130-095-066	N/A
	Mouse IL-6	Miltenyi	130-096-685	N/A
	Mouse IL-23	Miltenyi	130-096-677	N/A

Supporting TABLE S2. The Sequences of Primers

	Gene symbols	Forward	Reverse
Primers for mouse mRNAs	*Il17a*	TTTAACTCCCTTGGCGCAAAA	CTTTCCCTCCGACTTGACAC
	Il17f	TGCTACTGTTGATGTTGGGAC	AATGCCCTGGTTTTGGTTGAA
	Il22	ATGAGTTTTTCCCTTATGGGGAC	GCTGGAAGTTGGACACCTCAA
	Il23r	TTCAGATGGGCATGAATGTTTCT	CCAAATCCGAGCTGTTGTTCTAT
	Rorc	GGGACAAGTCATCTGGGAT	CAATGCTGGCATCGGTT
	Bcl2l1	GACAAGGAGATGCAGGTATTGG	TCCCGTAGAGATCCACAAAAGT
	Bcl2	ATGCCTTTGTGGAACTATATGGC	GGTATGCACCCAGAGTGATGC
	Myc	AGTGCTGCATGAGGAGACAC	GGTTTGCCTCTTCTCCACAG
ChIP primers	*Il17a*	AAAGAGCTCAGATCAAAGGG	TATGGGCATGAGCAAAGTG
	Il23r	CTGCCAGGCAAGAATTTAC	GCTTTCGAGAACACCACA
	Bcl2l1	CAGGAGGCACAGCAAAGAAG	TCCCCAGAGTAGTTCCATGTG
	Irrelevant region-1 (Hbb)	GCTCTGGGTACTCCCTCTGA	GCAAATGTGTTGCCAAAAAG
	Irrelevant region-2 (Negative)	GGGCGAGCTGGATATCTTATT	CCTCATGCACAGACTGAAAGA
sgRNA primers	*sgCbfb*	ACCGCCTTGCAGATTAAGTACAC	AAACGTGTACTTAATCTGCAAGG
	sgLgals1	ACCGATCCTCGCTTCAATGCCCA	AAACTGGGCATTGAAGCGAGGAT
	sgPpib	ACCGTCTTATCGTTGGCCACGGA	AAACTCCGTGGCCAACGATAAGA
	sgPurb	ACCGCAAGCTCATCGACGACTAC	AAACGTAGTCGTCGATGAGCTTG
	sgRbm14	ACCGTGCGGTCGCGACCAAAGAA	AAACTTCTTTGGTCGCGACCGCA
	sgRorc	ACCGCGGGGTTATCACCTGTGAG	AAACCTCACAGGTGATAACCCCG
	sgRpa1	ACCGACTCACTTGGACTGGTACG	AAACCGTACCAGTCCAAGTGAGT
	sgRunx1	ACCGCTTACTTCGGGGTTCTCGG	AAACCCGAGAACCCCGAAGTAAG
	sgRunx3	ACCGCTAAGCGCGCAGGCAACCG	AAACCGGTTGCCTGCGCGCTTAG
	sgSatb1	ACCGCTAAGCGCGCAGGCAACCG	AAACGCTGAGGACTGATCGGTGT
	sgSsbp1	ACCGTTACTTGGACGAGTAGGTC	AAACGACCTACTCGTCCAAGTAA
	sgTardp	ACCGACATATGATAGATGGGCGA	AAACTCGCCCATCTATCATATGT
	sgXrcc1	ACCGCCAAAAATGCGAACACGGT	AAACACCGTGTTCGCATTTTTGG

	NoNT sgRNA	ACCGAAACTCGCCCGCGTCATAT	AAACATATGACGCGGGCGAGTTT

Materials & Methods

Identification of IMU-935. IMU-935 was developed and patented by Immunic Therapeutics, which also provided us with the compound for research purposes. The company has conducted and passed preclinical toxicity study and phase I clinical trial, enabling the progression of IMU-935 into Phase II clinical trial. Here is a brief description of how IMU-935 was identified, as provided by the company. *In silico* screening against RORγ was performed at 4SC AG (a German biotech company) with a library of around 25 millions virtual compounds. The 300 most promising compounds were used for docking studies and then further investigated by testing their activity against IL-17A and IL-17F release in stimulated human PBMCs. A selection of the most promising compounds was further tested against RORγ in a cellular reporter assay from Indigo biosciences. Active compounds were further modified by use of medicinal chemistry and tested for their SAR (structure activity relation) in both IL-17A and IL-17F release and RORγ reporter assay. Around 750 molecules were even further characterized for activity and drug like properties and are patented by Immunic Therapeutics that provides the compound for this study. IMU-935 has the best combination of drug properties including affinity, toxicity and metabolism for further drug development.

Toxicity of IMU-935. IMU-935 was developed and patented by Immunic Therapeutics. The company has performed extensive toxicity studies including clinical trial. I list those studies and cite references, which is provided by the company: 1) In a published human clinical trial study (1), IMU-935 demonstrated a favorable safety and tolerability profile, with no significant adverse effects observed at single doses ranging from 25 to 400 mg or at multiple daily doses of 150 mg, compared to placebo. It is thus well tolerated across a range of concentrations and dosing

regimens. In this clinical trial study, IMU-935 did not cause adverse effects on participants' vital signs, including electrocardiogram (ECG), fecal calprotectin (intestinal inflammation indicator), or liver safety biomarkers. The conclusion of this clinical trial study is that IMU-935 was safe with no-dose-limiting toxicities and had a PK profile that support once-daily dosing; 2) A published 7-day tolerability study in preclinical animal (mice) study using a deuterated IMU-935 at 100 mg/kg revealed no significant changes in body weight, hematology, or clinical chemistry parameters (2)*;* 3) In our own thymocyte function study, oral administration of IMU-935 (100 mg/kg, b.i.d.) for 28 days did not affect body weight in mice as shown in Supplementary Fig. 4H; 4) Our *in vitro* study showed that IMU-935 does not affect $CD4^+$ T cell proliferation or survival. It has a slight effect on $CD8^+$ T cell proliferation, but no impact on $CD8^+$ T survival. These results are presented in the new Supplementary Fig. 2A-2B; 5) Comprehensive in vitro safety assessments of IMU-935 were conducted to ensure it met key toxicity criteria:
a) SafetyScreen44 panel – This assay screens for 44 potential off-targets known to cause toxicity if unintentionally modulated by a drug (3); b) Kinase panel – This test evaluates potential toxicity by examining the compound's interactions with a broad range of kinases; c) Ames test – Used to rule out mutagenic potential of the compound (4); d) Micronucleus test – Performed to exclude genotoxic potential by detecting the formation of micronuclei (5).

Specificity of IMU-935 for RORγt. Immunic Therapeutics conducted and provided us the following analyses to demonstrate the specificity of IMU-935: 1) In a nuclear hormone receptor panel from DiscoverX, IMU-935 showed no activity against 19 nuclear receptors at a concentration of 3 μM, which is more than 75-fold higher than its IC_{50} for RORγt; 2) Two additional nuclear receptors—RORα and RORβ, the closest homologs of RORγt—were also tested. IMU-935 demonstrated over 75-fold selectivity for RORγt compared to RORα and RORβ 3) In the KINOMEscan platform, which includes over 450 kinases and disease-relevant mutant

variants, IMU-935 showed no interaction with any kinases at 3 μM, again more than 75-fold higher than its IC_{50} for RORγt ; 4) In the SafetyScreen44 panel, which includes 44 receptors, ion channels, and transporters, IMU-935 (at 3 μM) showed no significant binding except for monoamine oxidase A (at 52.1%) and kappa opioid receptor (at 25%). However, since these effects were observed only at a concentration 75 times higher than the IC_{50} for RORγt, the activity was not considered significant enough to warrant further investigation.

Modulators of RORγt. IMU-935, MRL-871, cintirorgon, Cpd 1-JCI, AZD-0284, PF-06747711, GNE-6468, Cpd2, Cpd 1-PLoS are prepared or purchased by Immunic Therapeutics.

Antibodies and cytokines. Information for antibodies and cytokines is provided in Supporting Table S1.

Protein-protein docking protocol. The RORγt-CBFβ docking simulation was executed using the ZDOCK website (10.1093/bioinformatics/btu097). The RORγt structure was procured from the Alpha-Fold model associated with the UniProt entry A0A0G2JGZ6, while the CBFβ structure was derived from the crystallographic data of structure 3WTS (https://doi.org/10.1016/j.jmb.2014.07.020). During the docking process, RORγt residues L410, E412, and R407 were specifically designated as part of the RORγt binding interface. No specific inclusion or exclusion was stipulated for CBFβ residues, nor for any other residues in RORγt. The ZDOCK website generated the top 10 optimal docking poses, from which we selected the best pose for subsequent Molecular Dynamics (MD) simulation.

Molecular dynamics simulation. We constructed two MD simulation systems: one involved the RORγt-CBFβ complex, derived from the ZDOCK docking described earlier, and the other consisted of the RUNX1-CBFβ complex, extracted directly from the crystal structure 3WTS. Both simulation systems were established via CHARMM-GUI (10.1002/jcc.20945; 10.1021/acs.jctc.5b00935). Neutral patches were added to the N-terminal and C-terminal of the proteins, and all Histidine residues were assigned as HSD. The protein complexes were parameterized and ensconced in a cubic box of TIP3 water, maintaining a minimum margin of 10 Å from the protein surface. This water-filled simulation system was subsequently neutralized with 0.15 M KCl. The simulation systems underwent energy minimization via the GROMACS package (10.1016/0010-4655(95)00042-E), leveraging the CHARMM36m force field (10.1038/nmeth.4067). Restraints of 10 kcal/mol-Å^2 were applied on all backbone heavy atoms. Following minimization, the systems were gradually heated from 0 K to 310 K in 0.2 ns, under the NVT ensemble and the Nosé-Hoover thermostat (10.1063/1.449071). After the heating phase, a 60 ns equilibration simulation was conducted using the NPT ensemble. This process applied a harmonic positional restraint on the proteins and ligand heavy atoms, initially setting the constraints force constant at 10 kcal/mol-Å^2. This was subsequently reduced to 5 kcal/mol-Å^2 and then incrementally decreased to 0 kcal/mol-Å^2 over a 5 ns window for each 1 kcal/mol-Å^2 reduction. Pressure regulation was achieved using the Parrinello-Rahman method (10.1063/1.328693), coupling the simulation systems to a 1 bar pressure bath. The final frame from the equilibrium process was selected as the initial conformation for 5 separate, unrestrained NPT simulations at 310 K. Each of these simulations ran for 1000 ns. The LINCS algorithm was implemented in all simulations to constrain all bonds and angles of water molecules, with a 2 fs timestep used for integration. A cutoff of 12 Å was assigned for non-bonded interactions, and the Particle Mesh Ewald method (10.1063/1.464397) was used to manage long-range Lennard-Jones interactions. MD snapshots were recorded at 20 ps intervals.

Contact frequency analysis. The computation of residue contacts between RORγt/RUNX1 and CBFβ was accomplished using the tool get_contact (get_contact.io). We employed the tool's default parameters to delineate these contacts, and all contact types were taken into consideration. The frequency of these contacts was subsequently evaluated using the get_contact_frequency.py module.

Human Thymus Sample Acquisition and Processing. Human thymic tissue was collected in California Pacific Medical Center from a female donor (age 21) by International Institute for the Advancement of Medicine (https://iiam.org/) with appropriate written consent and approval from the IIAM Authority Ethics Committee. Fresh thymus specimens were cut into <1 mm^3 segments and transferred to a 50 mL conical tube. Thymus were digested with 1.6 mg/mL collagenase type IV (Worthington) in RPMI 1640 medium (supplemented with 10% FBS, 1% penicillin-streptomycin, and 2 mM L-glutamine) for 30 minutes at 37°C with intermittent shaking. Digested tissue was passed through a 100 µm filter, and cells were collected by centrifugation (500 g for 5 minutes at 4°C). Cells were treated with 1X red blood cell lysis buffer for 5 minutes at room temperature and washed once with PBS containing 5% FBS and 2 mM EDTA prior to inhibitors treatment.

Human peripheral blood mononuclear cells (PBMC). PBMC were isolated from healthy volunteers using density centrifugation with the filter containing blood separation tubes (Greiner) and Pancoll (Pan-Biotech) human according to the manufacturer’s manual. The remaining red blood cells were lysed using erythrocyte lysis buffer (8.29 g NH_4Cl, 1 g $KHCO_3$, 0.037 g EDTA in 1 L H_2O). After red cell removal, cells were stimulated with 2 µg/mL PHA (Sigma) and cultured

in RPMI 1640 stable Glutamine (Pan-Biotech) with 10% dialyzed FCS EU (Pan-Biotech) and 100 U penicillin/ 100 µg/mL streptomycin (PAA laboratories) in the absence or presence of IMU-935 in a 5-fold serial dilution from (0.0128 to 1000 nM) for 2 days.

Luminex analysis of cytokines in human PBMC. Supernatant was used to assess the level of secreted cytokines. The Luminex panel (Bio-rad) consisted out of IL-17A, IL-17F, IFNγ, IL-4 and IL-22. The assay was performed according to manufacturer's manual. Acquisition was performed on the BioPlex200 (Bio-Rad) and data was analyzed using the corresponding software.

RORγt luciferase reporter assay in human PBMC. Human RORγt luciferase reporter assay (Indigo Biosciences) was performed according to manufacturer's manual. In short, cells were rested for 4-6 hours after plating. Next, they were either treated with different concentration of IMU-935 in a serial dilution or with vehicle (DMSO). After 22-24 hours medium was discarded and Luciferase detection reagent (Indigo Biosciences) was added for 5 minutes. Next, the intensity of light emission (Relative Luciferase Units) from each assay well was quantified using a plate-reading luminometer, Genios Pro 96/384 Multifunction Microplate Reader (Tecan).

Luciferase assay. HEK293T cells (ATCC) were cultured in Dulbecco's modified Eagle's medium supplemented with 10% FBS, 2 mM glutamine, penicillin (100 U/mL), and streptomycin (100 mg/mL). A total of 2×10^5 cells were seeded to each well of a six-well plate and transfected with the reporter vectors (400 ng), pSV40-Renilla luciferase vector (200 ng), and expression vectors (2 µg) using BioT transfection reagent (Bioland Scientific, Paramount, CA). The same amount of plasmid DNA was used by adjusting with an empty vector. Luciferase

activity was measured using the Dual-Luciferase Reporter Assay System (Promega, Madison, WI) per the manufacturer's instruction and normalized against Renilla luciferase activities. "Relative luciferase activities" were plotted with further normalization of luciferase activities of each group to the pGL3-basic reporter vector plus the empty vector group. RORγt luciferase reporter was prepared by inserting of 3 RORγt-binding element (RORBE) upstream of a mini TK promoter into pGL3-basic vector (Promega). RUNX1 luciferase reporter containing 4 RUNX1 consensus sites positioned upstream of a mini TK promoter was a gift from Alan D. Friedman.

Cloning. Murine *Rorc* transcript variant 2, *Cbfb* transcript variant 1 and *Satb1* transcript variant 1 were PCR amplified from the cDNA of WT $CD4^+$ T cells. The coding sequences were inserted into a MIGR1 vector (gift from Warren S. Pear). Mutated or truncated RORγt constructs were generated using a Q5 site-directed mutagenesis kit (New England Biolabs, catalog #: E0554S) with PCR primers designed using the online NEBaseChanger™ tool. Additional 3xFlag- or HA-tag DNA sequences were fused to the 3' end of *Rorc2* or 5' end of *Cbfb* and *Satb1* coding sequences for some immunoprecipitation experiments. These fusions were incorporated into the MIGR1 or pcDNA3 vectors. The IRES-EGFP from MIGR1 and the RUNX1-IRES-EGFP from MSCV-RUNX1 (a gift from Ichiro Taniuchi, RIKEN Center for Integrative Medical Sciences, Japan) were digested to replace the GFP sequence in the Vsg-GFP lentiviral vector (a gift from Inder M. Verma, Salk Institute for Biological Studies).

CRISPR/Cas9 mediated genomic DNA deletion. To achieve genomic deletion of target genes, single-guide RNA (sgRNA) sequences were selected from a library (Addgene, library 67988) and cloned to pMSCV-U6sgRNA(BbsI)-PGKpuro2ABFP vector (Addgene, 102796). sgRNA primers are listed in Supporting Table S2.

Virus preparation. Retroviral expression plasmids were transfected into Platinum-Eco packaging cells using BioT transfection reagent (Bioland Scientific), fresh medium was changed 24 hours after transfection. Viral supernatants were collected 48 and 72 hours later, passed through 0.4 μm filters (Millipore), and were used immediately transduce T cells or stored at −80 °C until use. Lentivirus plasmids, including the expression vector (Vsg-IRES-EGFP or Vsg-RUNX1-IRES-EGFP), packaging vector (gag-pol), and VSV-g envelope vector, were co-transfected into HEK-293T cells at 3:2:1 ratio using BioT transfection reagent (Bioland Scientific). Fresh medium was replaced 10 hours after transfection. Viral supernatants were collected 24 and 48 hours post-transfection, passed through 0.45 μm filters (Millipore). The viral supernatant was supplemented with 20% FBS and 5 ng/mL recombinant mouse IL-7 (PeproTech) and then used immediately to transduce thymocytes.

***In vitro* Th17-differentiation and transductions of retroviral vectors**. Naive $CD4^+$ T cells were purified from mice spleen by using MojoSort™ Mouse CD4 T Cell Isolation Kit (Biolegend). Suspensions of 4×10^5 cells per mL RPMI 1640 medium containing 10% FBS, 1% penicillin-streptomycin, 2 mM l-glutamine and 50 μM β-mecaptoethanol, and were cultured in 24-well plates pre-coated with 0.2 mg/mL rabbit anti-hamster (MP Biomedicals). The cells were activated with anti-CD3 (0.25 μg/mL) and anti-CD28 (1 μg/mL) overnight, followed by spin-infection with retrovirus supernatants at 2,500 rpm for 2 hours at 30°C, in the presence of 8 μg/mL polybrene (Sigma-Aldrich). After spin infection, viral supernatant was replaced by culture medium with Th17 polarizing cytokines (2 ng/mL TGF-β, 20 ng/mL IL-6, 20 ng/mL IL-23, 5 μg/mL anti-IL-4 and 5 μg/mL anti-IFN-γ) for *in vitro* differentiation. 12 hours after differentiation, IMU-935 was additionally added to the medium and incubated for 48 hours.

***In vitro* T cell-development assay.** Double-negative (Thy1.2^{+}CD4^{-}CD8^{-}) thymocytes were sorted out and cultured at 5 × 10^5 cells per mL overnight on an 80% confluent OP9-DL4 monolayer in flat-bottomed 24-well culture plates with αMEM medium supplemented with 20% FBS, 1% penicillin-streptomycin, 2 mM L-glutamine, and 5 ng/mL recombinant mouse IL-7 (PeproTech), with or without drug treatment for 72 hours, co-cultures were harvested for flow cytometry analysis.

Thymocyte apoptosis assay. Thymocytes were freshly isolated from wild-type, *Rorγt*$^{-/-}$ mice and were suspended in RPMI-1640 medium supplemented with 10% FBS, 1% penicillin-streptomycin and 2 mM l-glutamine at 1 × 10^6 cells per mL with or without reagents treatments. For apoptosis staining, thymocytes were incubated with anti-Thy1.2 antibody and a fixable live/dead near-infrared dye (Thermo Fisher Scientific) for 15 minutes, followed by 2 washes. Then the cells were stained with Annexin V in Annexin V binding buffer for 15 minutes.

Flow cytometry. For surface staining, cells were stained with antibodies in PBS with 2% FBS and 1 mM EDTA at 4°C for 15 minutes. For cytokine staining, cells were pre-stimulated with phorbol 12-myristate 13-acetate (50 ng/mL, Sigma-Aldrich) and ionomycin (0.75 μg/mL, Sigma-Aldrich) in the presence of Golgistop™ (BD Bioscience) for 3 hours ahead of staining. Cells were stained with surface markers and/or fixable LIVE/DEAE™ dye (Thermo Fisher Scientific) at 4°C for 15 minutes. Cells were fixed and permeabilized with CytoFix/CytoPerm buffer (BD Bioscience) for 20 minutes followed by staining for cytokines in the Perm/Wash buffer (BD Bioscience) for 15 minutes. Subsequent analysis was performed in the BD LSRFortessa flow cytometer. Antibodies used for flow cytometric assay were summarized in Supporting Table S1.

Immunoprecipitation. For immunoprecipitation in Th17 cells, CD4 T cells were isolated from *Rorγt*$^{-/-}$ mice or CRISPR/Cas9-EGFP mice spleen using MojoSort™ Mouse CD4 T Cell Isolation Kit (Biolegend). The cells were activated with anti-CD3 (0.25 μg/mL) and anti-CD28 (1 μg/mL) overnight, followed by spin-infection with retrovirus supernatants at 2,500 rpm for 2 hours at 30°C, in the presence of 8 μg/mL polybrene (Sigma-Aldrich). After spin infection, viral supernatant was replaced by culture medium with Th17 polarizing cytokines (2 ng/mL TGF-β, 20 ng/mL IL-6, 20 ng/mL IL-23, 5 μg/mL anti-IL-4 and 5 μg/mL anti-IFN-γ) for *in vitro* differentiation. 12 hours after differentiation, IMU-935, Cpd-1, or MRL-871 were additionally added to the medium and incubated for 48 hours. For immunoprecipitation in thymocytes, the thymocyte were isolated from 4–6-week-old C57BL/6 mice and cultured at a density of 2×10^6 cells per mL RPMI-1640 medium supplemented with 10% FBS, 1% penicillin-streptomycin and 2 mM l-glutamine at 2×10^6 cells per mL with or without drug treatments for 24 hours. For some experiments, double-positive (DP) thymocytes (Thy1.2$^+$CD4$^+$CD8$^+$) were sorted out and cultured at a density of 2×10^6 cells per mL on an 80% confluent OP9-DL4 monolayer, which had been plated one day prior in flat-bottomed 24-well culture plates. Co-culture was incubated with αMEM medium supplemented with 20% FBS, 1% penicillin-streptomycin, 2 mM L-glutamine, and 5 ng/mL recombinant mouse IL-7 (PeproTech) and allowed to stabilize for 4 hours before treatment or lentiviral transduction. Lentiviral transduction was performed by spin infection. Lentiviral supernatants were supplemented with 8 μg/mL polybrene (Sigma-Aldrich) and added directly to the co-culture. Plates were centrifuged at 2,500 rpm for 2 hours at 30°C. After spin infection, viral supernatants were replaced with fresh αMEM complete medium containing 5 ng/mL recombinant mouse IL-7 (PeproTech) and incubated overnight, followed by treatment with IMU-935 at the indicated concentration for 24 hours. After drug treatment, viable DP thymocytes were enriched using EasySep™ Dead Cell Removal Kit (STEMCELL

Technologies). Purified live cells were counted, a total of 5 × 10^6 cells were used for whole-cell lysate preparation. As a negative control group, unsorted thymocytes or sorted DP thymocytes were isolated from 4–6-week-old *Rorγt*$^{-/-}$ mice freshly and used for whole-cell lysate preparation. Cells were lysed in Pierce® IP lysis buffer (25 mM Tris-HCl pH 7.4, 150 mM NaCl, 1% NP-40, 1 mM EDTA, 5% glycerol). Protein from cell extracts was incubated overnight with relevant antibodies and protein A/G plus-agarose (Santa Cruz) for 4 hours at 4°C. After incubation, beads were washed four times. Eluted protein was resolved using SDS-PAGE and transferred to Nitrocellulose Membrane (LI-COR), and immunoblot analysis was performed using relevant primary antibodies and secondary antibodies.

Immunohistochemistry. Hematoxylin and eosin (H&E) staining and terminal deoxynucleotidyl transferase-mediated deoxyuridine triphosphate nick-end labeling (TUNEL) staining were carried out by the Solid Tumor Pathology Core at the City of Hope. TUNEL staining were performed using ApopTAG® Plus Peroxidase *In Situ* Apoptosis Kit (Sigma Aldrich) according to the manufacturer's instructions. For the counting of TUNEL-positive cells, three fields/slide at ×100 magnification were randomly selected, and percentage of TUNEL-positive staining was assessed using ImageJ (National Institutes of Health) software. Data were obtained from two to three samples per condition.

Quantitative real-time PCR (qRT-PCR). Total RNA was isolated using RNeasy® mini kit (Qiagen) and reverse-transcribed to obtain complementary DNAs (Tetro cDNA synthesis kit, Bioline). qRT-PCR was performed using PowerUp™ SYBR™ Green Master Mix (Thermo Fisher Scientific) in a QuantStudio™ 3 Real-Time PCR System (Thermo Fisher Scientific). The relative messenger RNA (mRNA) levels were normalized to the control gene encoding β-actin or Gapdh

by calculating with 2^ΔCt method. The primers used in qRT-PCR assays are listed in Supporting Table S2.

Induction and assessment of EAE. Active EAE was induced and assessed as previously described (Zhiheng He, NI). In brief, 9-week-old female C57BL/6 mice were immunized with 200 mg $MOG_{35–55}$ (Hooke Laboratories) in Complete Freund's adjuvant (CFA) by subcutaneous injection at two dorsal sites at day 0, followed by two intraperitoneal injections of 80 ng pertussis toxin (PTX) at day 0 and 1. IMU-935 was dissolved in PEG400 and administered by oral gavage (p.o.) throughout the experiment at 100 mg/kg twice per day (b.i.d). For passive EAE, *Rag1*$^{-/-}$ mice were adoptively transferred with 1 x 10^5 TCR^{MOG}-expressing (Tg^{Tcr2D2}) Th17 cells that were differentiated under Th17 polarization condition with or without IMU-935, followed by an immunization with MOG at 7 days post-transfer. All polarized cells were sorted for CD4 and GFP expression prior to adoptive transfer into mice. Severity of EAE was monitored and a clinical score from 0 to 5 was assigned: 0=no disease; 0.5=partially limp tail; 1=paralyzed tail; 2=hind limb weakness; 3=hind limb paralysis; 4=hind and fore limb paralysis; 5=moribundity and death.

Mice treatment for thymus function test. 8-week-old C57BL/6 male mice were treated with vehicle control, IMU-935 (100 mg/kg, Immunic Therapeutics), Cpd1-JCI (40 mg/kg, Immunic Therapeutics) or cintirorgon (30 mg/kg, Synnovator) by oral gavage twice a day (b.i.d) for 4 weeks. In a short-term treatment model, mice were treated with IMU-935 (100 mg/kg), MRL-871 (100 mg/kg, 1ClickChem), or cintirorgon (30 mg/kg) by oral gavage twice a day (b.i.d) for 3 days. Whole thymuses were collected and subjected to immunohistochemistry, mRNA preparation, and flow cytometry analysis.

RNA-Sequencing and analysis. RNA samples were prepared using RNAeasy mini kit (Qiagen). Quality control, library preparation, and sequencing were performed at Novogene. Analysis was performed through Partek Flow Genomic Analysis Software. Briefly, the sequence reads were aligned to mouse whole genome (GRCm38) with validation of quality through prealignment and postalignment quality assurance/quality control (QA/QC). Aligned reads were further subjected to quantification using the Partek expectation/maximization (E/M) algorithm and normalization to counts per million with 0.001 added to each. The identification of differentially expressed features was performed through the Partek gene specific analysis (GSA) algorithm that applies multiple statistical models to each gene. Genes with total counts over 30 were considered to be statistically expressed in the cells. The expression values of pathogenic genes were extracted and subjected to ingenuity pathway analysis (IPA), gene set enrichment analysis (GSEA), and network analysis.

ChIP-Sequencing and analysis. ChIP samples were prepared using ChIP-IT Express Enzymatic kit (Active Motif). Briefly, cells were fixed in 1% formaldehyde at room temperature for 10 minutes to cross-link proteins with chromatin. The reaction was stopped with incubation in glycine for 5 minutes. Genomic DNA was fragmented with enzyme cocktail, reaction was stopped by adding 0.5 M EDTA. Sheared chromatin samples were centrifuged for 10 minutes at 15,000 rpm, and supernatant was incubated with anti-FLAG (M2, Sigma-Aldrich) overnight, followed by precipitation with protein G agarose beads. Beads complexed with DNA fragments were extensively washed, and DNA was eluted followed by reverse cross-linking. Recovered DNA was subjected to NovaSeq with 51-base pair (bp) paired-end sequencing length. Analysis was performed through Partek Flow Genomic Analysis Software. Total reads were aligned to mouse whole genome (GRCm38) using the Burrow-Wheeler aligner (BWA). Peaks were

identified with the model-based analysis of ChIP-seq 2 (MACS2) tool (version 2.1.1) and quantified with a minimum region size of 50 bp.

Pathway Analysis. The fold change and p value of differentially expressed genes (DEGs) extracted from RNA-sequencing analysis were uploaded to the core analysis in Ingenuity Pathway Analysis (IPA) platform (Qiagen). The canonical pathway analysis was used to predict the direction of the enriched pathway activity, and the upstream regulator analysis was used to identify the upstream regulators that predicted responsible for gene expression changes. IPA match analysis was used to compare our datasets with other publicly available datasets curated by IPA.

Gene set enrichment analysis (GSEA). Preranked Gene Set Enrichment Analysis was conducted for functional enrichment analysis with Gene Ontology (GO) resource obtained from mouse Molecular Signatures Database (MSigDB) collections v2022.1. Genes from edgeR results were ranked by the sign of logFC in combination with -log10 of the p-value for this analysis.

Mass spectrometry. Proteins were separated on 4-12% Bis-Tris NuPAGE gel and silver stained using Pierce Silver Stain for Mass Spectrometry kit (Thermo Fisher Scientific), followed by excision of gel bands and tryptic digestion. The digested samples were quenched and extracted with 50% ACN, 5% formic acid, followed by SpeedVac drying, and then processed with standard c18 stage-tip method. Sample was reconstituted with mobile phase A. 10% of each sample was loaded onto the column with a 1 hour gradient nano LC-MS/MS system (BGI

Americas). MS Data was searched against most updated Uniprot mouse database with Sequest analysis workflow to reveal basic protein profiling information.

References

1. Polasek, T. M. et al. Safety, Tolerability, and Pharmacokinetics of IMU-935, a Novel Inverse Agonist of Retinoic Acid Receptor-Related Orphan Nuclear Receptor gammat: Results From a Double-Blind, Placebo-Controlled, First-in-Human Phase 1 Study. Clin Pharmacol Drug Dev 12, 525-534, doi:10.1002/cpdd.1243 (2023).

2. Herrmann, A. et al. Orally bioavailable RORgamma/DHODH dual host-targeting small molecules with broad-spectrum antiviral activity. Antiviral Res 231, 106008, doi:10.1016/j.antiviral.2024.106008 (2024).

3. Bowes, J. et al. Reducing safety-related drug attrition: the use of in vitro pharmacological profiling. Nat Rev Drug Discov 11, 909-922, doi:10.1038/nrd3845 (2012).

4. Ames, B. N., Lee, F. D. & Durston, W. E. An improved bacterial test system for the detection and classification of mutagens and carcinogens. Proc Natl Acad Sci U S A 70, 782-786, doi:10.1073/pnas.70.3.782 (1973).

5. Fenech, M. The in vitro micronucleus technique. Mutat Res 455, 81-95, doi:10.1016/s0027-5107(00)00065-8 (2000).
