# Supplementary material for: Selective disruption of RORγt-CBFβ interaction by IMU-935 prevents RORγt-dependent Th17 autoimmunity but not thymocyte development
Source: J Clin Invest. 2026 Jan 2;136(1):e185942. doi: 10.1172/JCI185942 (PMC12721887; doi:10.1172/JCI185942)

Full unedited blot for Figure 6

Figure 6D

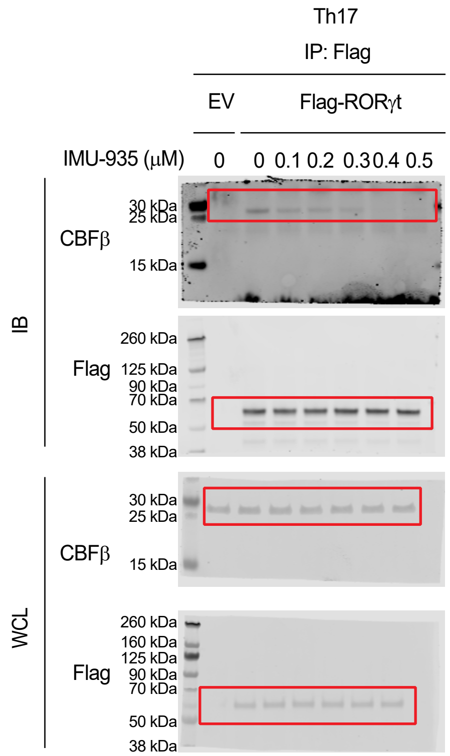

Figure 6E

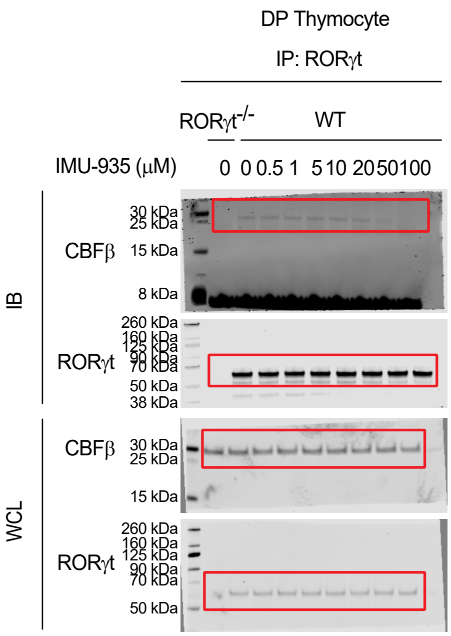

Figure 6G

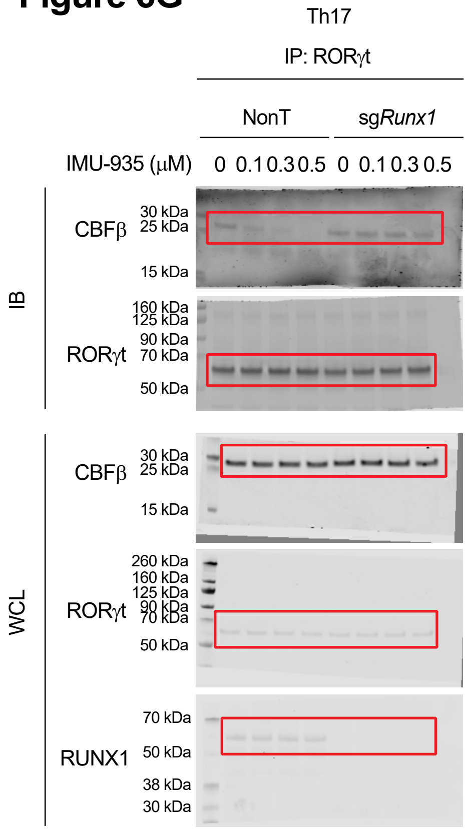

Figure 6H

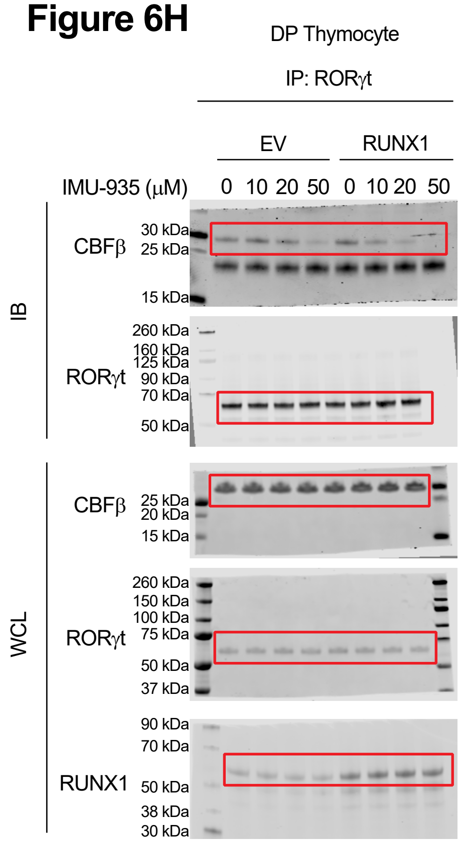

Full unedited blot for Figure 7

Figure 7A

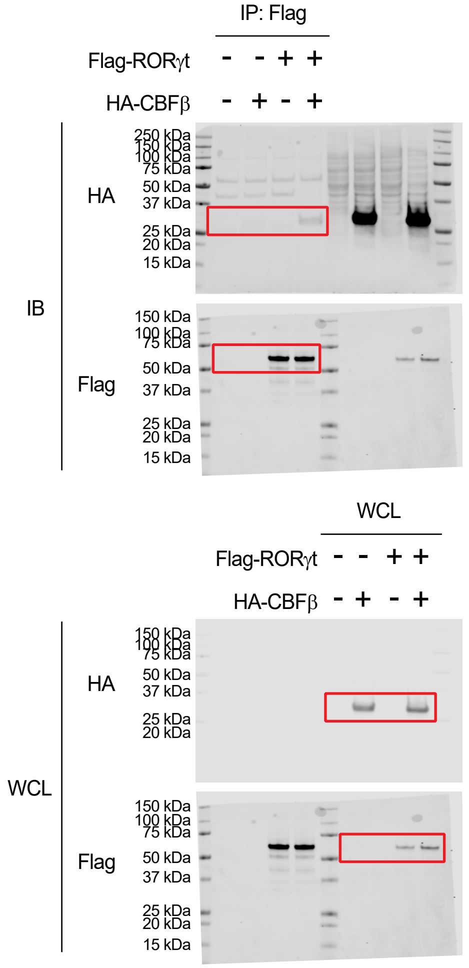

Figure 7B

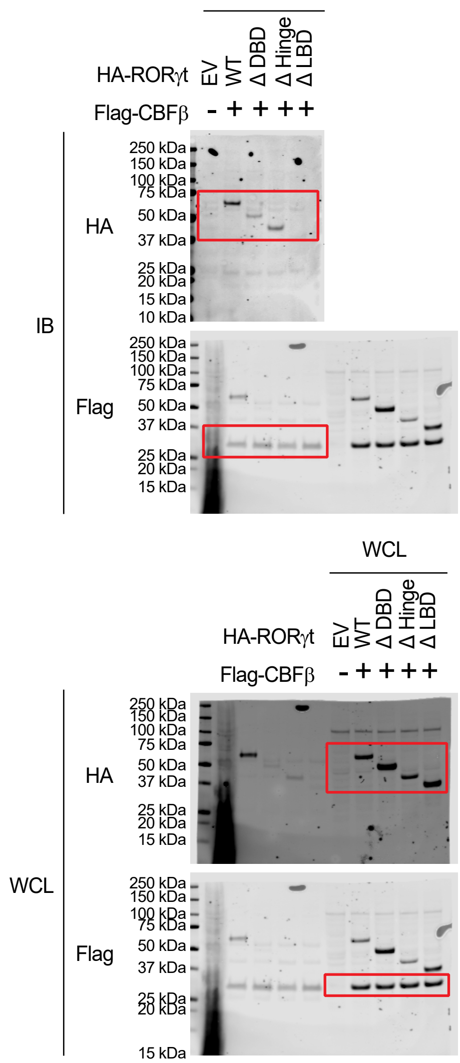

Figure 7C

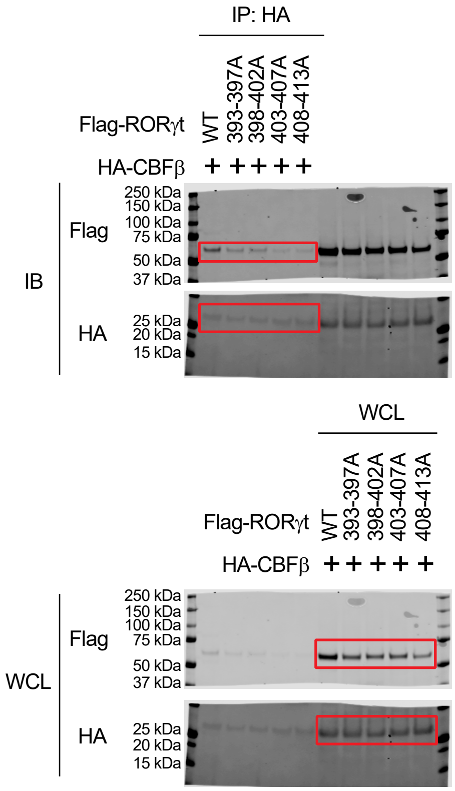

Figure 7F

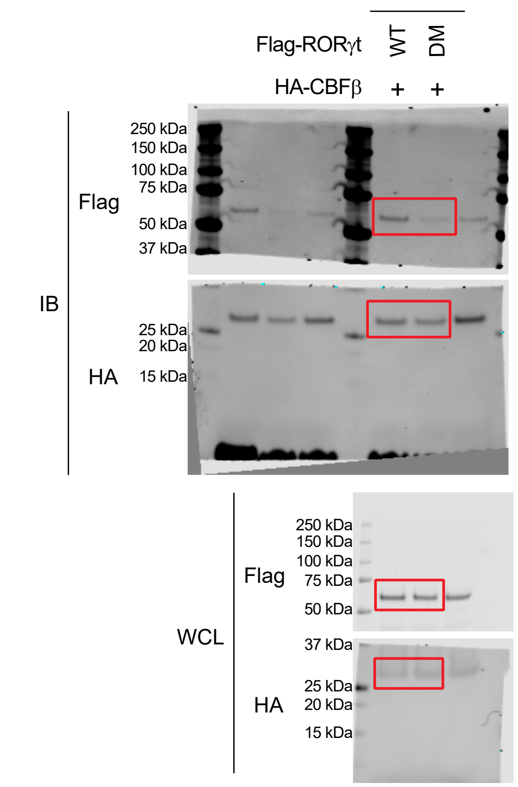

Full unedited blot for Figure 8

Figure 8A

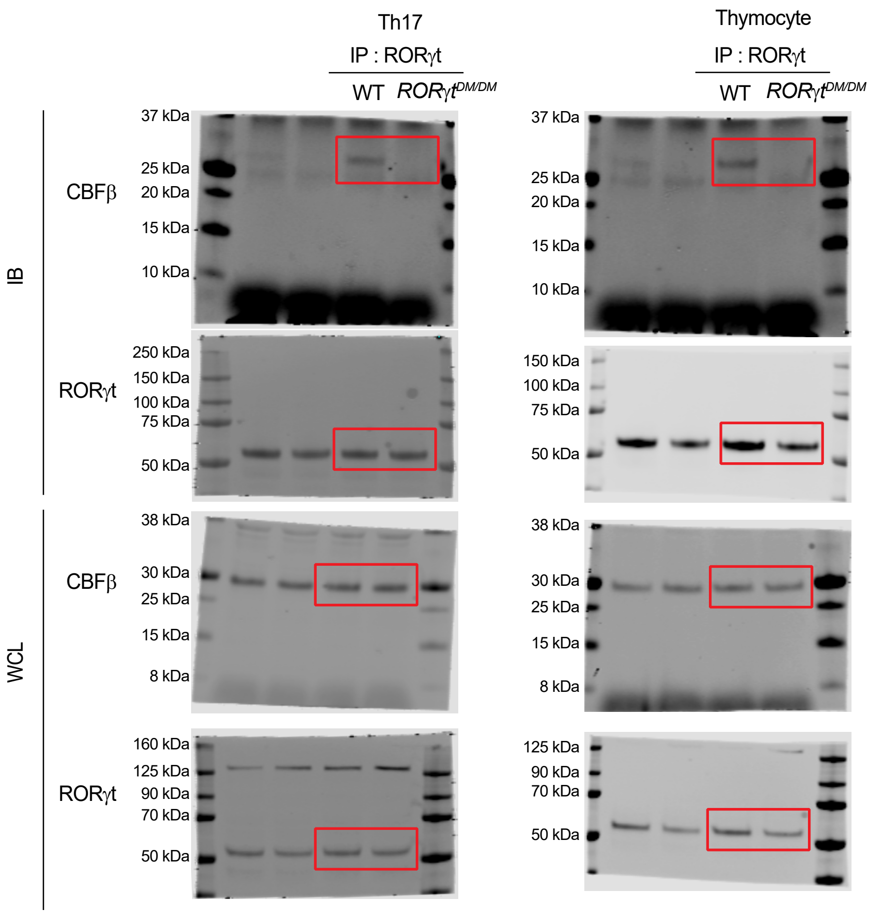

Full unedited blot for Suppl. Figure 6

Suppl. Figure 6F

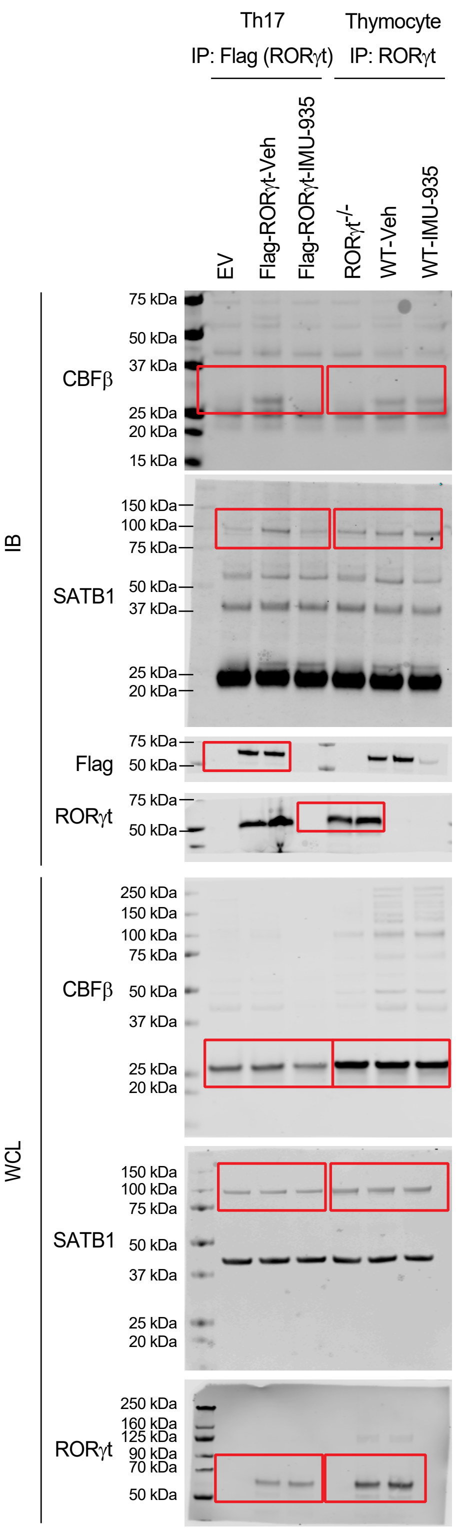

Suppl. Figure 6G

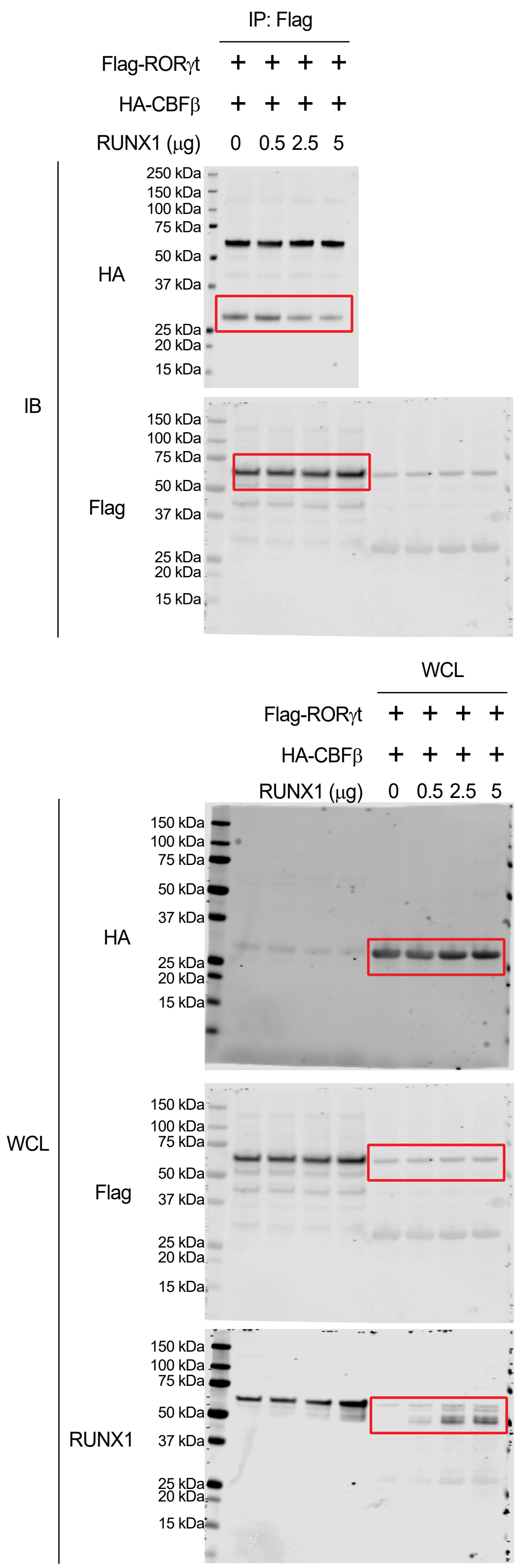

Suppl. Figure 6I

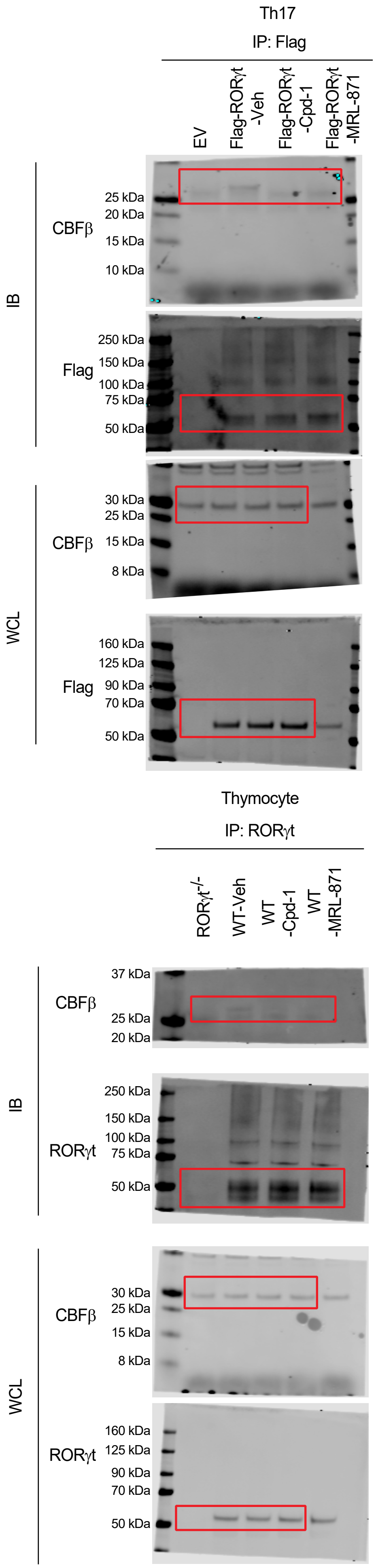

Full unedited blot for Suppl. Figure 7

Suppl. Figure 7A

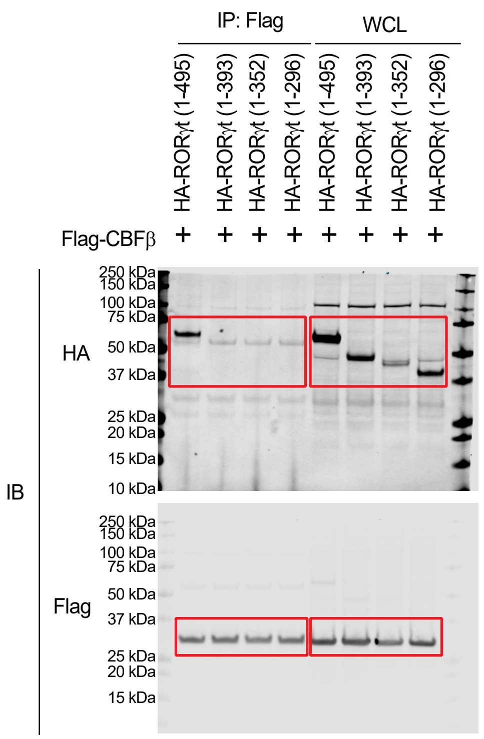

Suppl. Figure 7B

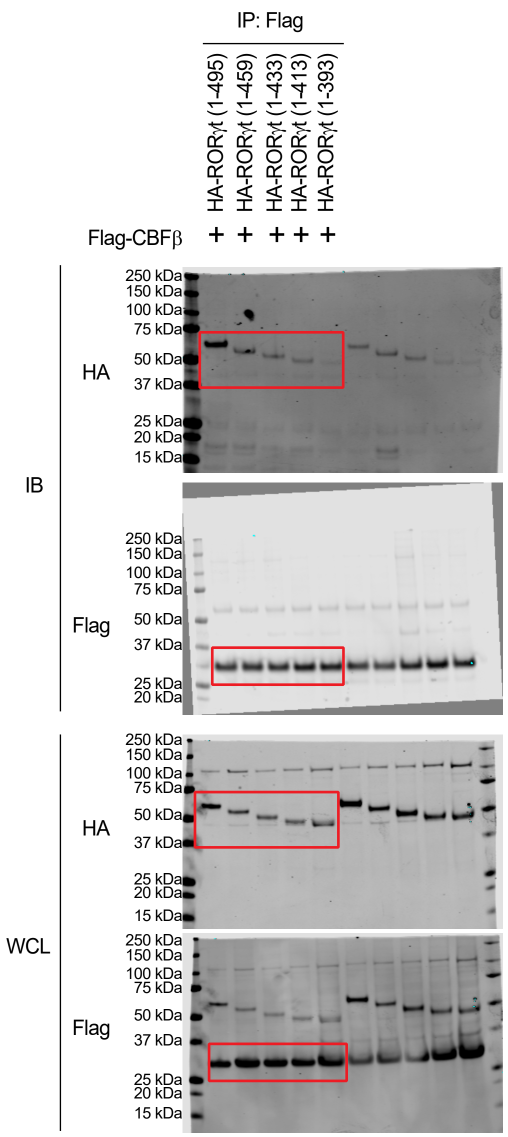

Suppl. Figure 7D

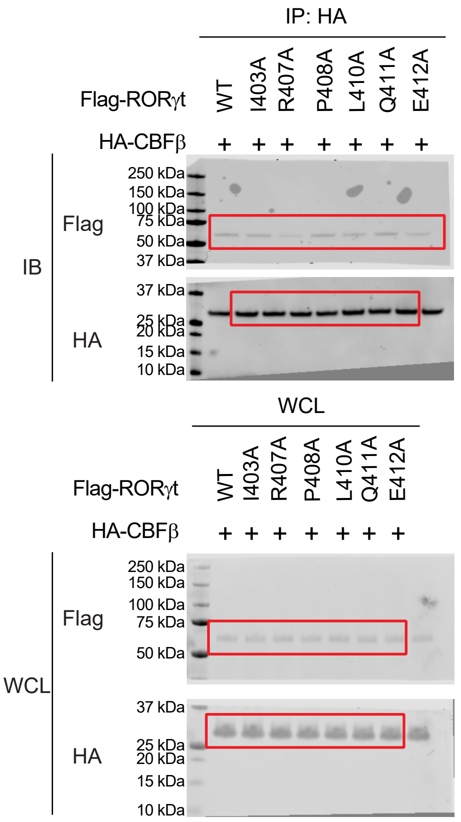

Supplement: Unedited blot and gel images [file jci-136-185942-s028.pdf]
